# Preconceptual administration of doxycycline in women with recurrent miscarriage and chronic endometritis: protocol for the Chronic Endometritis and Recurrent Miscarriage (CERM) trial, a multicentre, double-blind, placebo-controlled, adaptive randomised trial with an embedded translational substudy

**DOI:** 10.1136/bmjopen-2023-081470

**Published:** 2023-12-01

**Authors:** Joshua Odendaal, Naomi Black, Georgios Bouliotis, Jonathan Guck, Martin Underwood, Joanne Fisher, Siobhan Quenby

**Affiliations:** 1 University Hospitals Coventry and Warwickshire NHS Trust, Coventry, UK; 2 Division of Biomedical Sciences, Warwick Medical School, University of Warwick, Coventry, UK; 3 Warwick Clinical Trials Unit, Warwick Medical School, University of Warwick, Coventry, UK

**Keywords:** Reproductive medicine, Randomized Controlled Trial, Clinical Trial

## Abstract

**Introduction:**

Recurrent miscarriage is a common condition with a substantial associated morbidity. A hypothesised cause of recurrent miscarriage is chronic endometritis (CE). The aetiology of CE remains uncertain. An association between CE and recurrent miscarriage has been shown. This study will aim to determine if preconceptual administration of doxycycline, in women with recurrent miscarriages, and CE, reduces first trimester miscarriages, increasing live births.

**Methods and analysis:**

Chronic Endometritis and Recurrent Miscarriage is a multicentre, double-blind adaptive trial with an embedded translational substudy. Women with a history of two or more consecutive first trimester losses with evidence of CE on endometrial biopsy (defined as ≥5 CD138 positive cells per 10 mm^2^) will be randomised to oral doxycycline or placebo for 14 days. A subset will be recruited to a mechanistic substudy in which microbial swabs and preintervention/postintervention endometrial samples will be collected. Up to 3062 women recruited from 29 National Health Service (NHS) hospital sites across the UK are expected to be screened with up to 1500 women randomised in a 1:1 ratio. Women with a negative endometrial biopsy (defined as <5 CD138 positive cells per 10 mm^2^) will also be followed up to test validity of the tool. The primary outcome is live births plus pregnancies ≥24 + 0 weeks gestation at the end of the trial, in the first or subsequent pregnancy. Secondary clinical outcomes will also be assessed. Exploratory outcomes will assess the effect of doxycycline treatment on the endometrial microbiota, the differentiation capacity of the endometrium and the senescent profile of the endometrium with CE.

**Ethics and dissemination:**

Ethical approval has been obtained from the NHS Research Ethics Committee Northwest-Haydock (19/NW/0462). Written informed consent will be gained from all participants. The results will be published in an open-access peer-reviewed journal and reported in the National Institute for Health and Care Research journals library.

**Trial registration number:**

ISRCTN23947730.

**STRENGTHS AND LIMITATIONS OF THIS STUDY**

- This will be the largest randomised controlled trial assessing the role of antibiotics in improving live birth rates in women with a history of recurrent miscarriage.
- An integrated mechanistic design allows elucidation of both clinical and mechanistic outcomes.
- Generation of a bank of data-linked samples for further chronic endometritis (CE) research.
- Recruitment may be difficult given the use of CE testing, relative low cost of doxycycline and treatment in the private sector and the limited equipoise within the specialist population.
- A large number of secondary outcomes with reduced power for detection in this model, but balanced by the increased benefits of an integrated design.

## Introduction

Recurrent miscarriage is an important cause of reproductive associated morbidity. Defined as the loss of two or more pregnancies, it has a population prevalence of up to 3%.^1^ It is research priority within reproductive health.^2 3^ A *Lancet* review series highlighted the ongoing uncertainty around the aetiology of recurrent miscarriage.^1^ It is hypothesised that chronic endometritis (CE) is a cause of recurrent miscarriage.^4^ In CE, there is asymptomatic inflammation of the endometrium mediated by infiltration of the endometrial stroma by plasma cells.^5^ CE remains of uncertain aetiology being differentially attributed to dysbiosis of the endometrial microbiota and the presence of pathological species within the endometrium.^6–8^ There is not consensus around the diagnosis of CE.^9^ Traditionally, CE has been diagnosed on histology through the identification of infiltrating endometrial plasma cells.^10^ Recently, this has been augmented using CD138 immunohistochemistry.^11^ While this has resulted in improved sensitivity, the diagnostic thresholds remain variable and the impact on therapeutic outcome varies dependent on the CD138 threshold set.^9^ Liu *et al* evaluated different diagnostic methodologies demonstrating a technique based on whole section density to have the lowest intraobserver and interobserver variability.^11^ The study also identified a density threshold based on the 95% CI with a differential prevalence of 22.2% in those with reproductive failure in comparison to 5% in fertile controls (p<0.01). McQueen *et al* assessed the effect of antibiotic treatment in a cohort of women presenting with recurrent miscarriage or loss of a single fetus>10 weeks gestation.^12^ Following treatment with antibiotics, a similar cumulative live birth rate (LBR) was seen in the CE group in comparison to controls (88% treated CE vs 74% without CE; p=0.215). A significant increase in per pregnancy LBR pre-treatment and post-treatment for CE was also seen (7% pre-treatment LBR vs 56% post-treatment LBR; p<0.001). Similarly, a retrospective cohort study assessing for the presence of CE demonstrated a marked improvement in LBR in those with CE resolution following antibiotic therapy (78.4% LBR in resolved CE vs 17.5% LBR persistent CE; p<0.001).^13^ Doxycycline is the most commonly reported antibiotic therapy used in the treatment of CE. A single course has previously been reported to achieve histological resolution in 92% of cases.^14^ These findings, however, remain limited by sample size and methodological concerns including lack of randomisation and lack of untreated controls. Despite this however, commercial testing is now available within the private sector.^15^ Given the combined limitations in pathophysiological and clinical understanding of the condition, a novel translation approach is required. In view of this, the Chronic Endometritis and Recurrent Miscarriage (CERM) trial was developed as a novel translation approach to better determine the role of CE in recurrent miscarriage and the potential for therapeutic intervention.

### Study objectives

1. To determine if doxycycline administered prior to conception improves pregnancy outcome in women with recurrent miscarriage associated with CE.
2. To explore the mechanisms by which it could prevent miscarriage.
3. To determine if CD138 values predict miscarriage.

#### Primary outcome

The primary outcome is total live births plus ongoing pregnancies ≥24 + 0 weeks gestation at the end of the trial, in the first or subsequent pregnancy. This measure was chosen as the most meaningful outcome to patients.

#### Secondary outcomes

Given aetiological uncertainty, a cluster of both clinical and pathophysiological secondary outcomes have been selected. These include: ongoing pregnancy at 12 weeks gestation, time to first conception, anticipated time to first live birth, the proportion with livebirth ≥24 weeks gestation in the first pregnancy, pregnancy complications, type of miscarriage, ongoing pregnancy rates, LBRs in those excluded from randomisation as CE negative, the predictive value of CD138+ cell density on pregnancy outcome, the impact of intervention on endometrial CD138^+^ cell density and termination for social reasons.

#### Exploratory outcomes

Given the translational nature of the trial, a cluster of exploratory outcomes will also be assessed. These include an assessment of the effect of doxycycline treatment on the endometrial microbiota, the differentiation capacity of the endometrium and the senescent profile of the endometrium with CE.

## Methods and analysis

The protocol (V.8.3 22 June 2023) was designed in accordance with Standard Protocol Items: Recommendations for Interventional Trials guidelines.^16^ The trial has been prospectively registered on the ISRCTN registry (ISRCTN23947730).

### Study design

**Figure 1:**
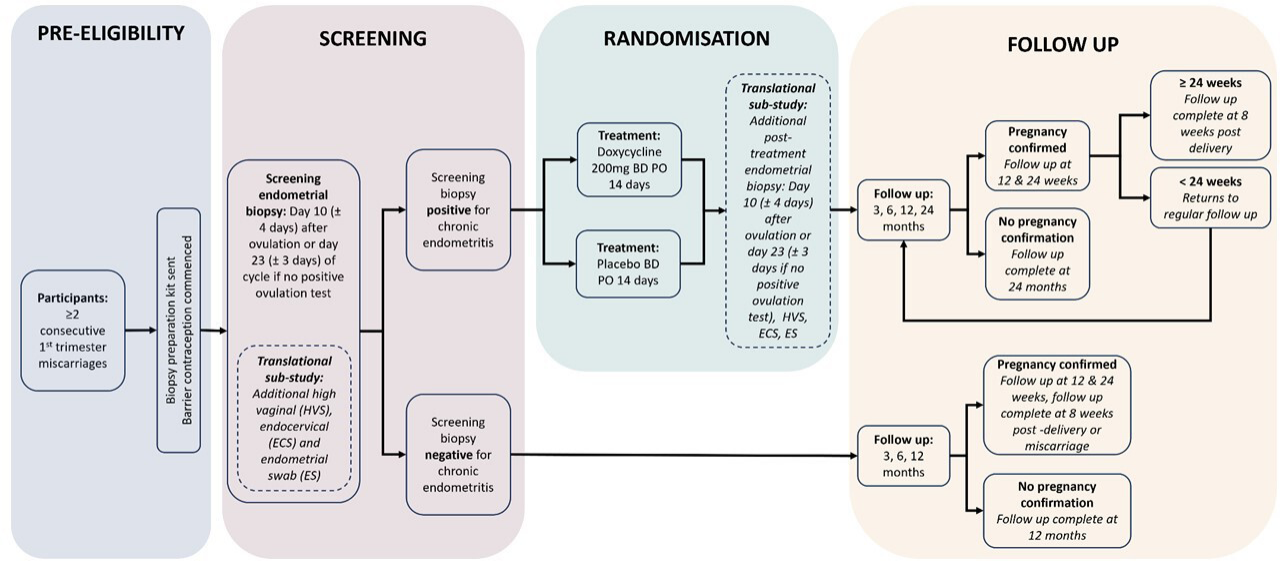
Study participant flow chart.

The CERM trial is a multicentre, double blind adaptive trial. It is a multiphased trial with a preeligibility phase, screening phase, randomisation phase and two separate follow-up pathways dependent on-screen status. These are summarised in figure 1. This is a trial of adaptive design with three prespecified interim stops to assess for futility at 100, 300 and 900 participants. Based on the unblinding results, the trial may stop after Data Monitoring Committee (DMC) recommendation, if there is low or negligible treatment efficacy. In addition, the use of ongoing pregnancy as a surrogate marker will also increase the trial flexibility while maintaining evidential rigour. The trial also has a parallel translational substudy assessing the endometrial and microbial effects of a diagnosis of CE and the impact of treatment on this. Using the clinical trial to develop a sample cohort will allow assessment of both the exploratory outcomes and allow further basic science assessment of the impact of CE with the aim of identifying the pathophysiological sequence of the condition alongside the potential mechanism of treatment effect.

The clinical trial is conducted and managed by the Warwick Clinical Trials Unit (WCTU). The trial has been sponsored by University Hospitals Coventry and Warwickshire (UHCW). The trial is funded by the Efficacy and Mechanism Evaluation Programme, a Medical Research Council (MRC) and National Institute for Health and Care Research (NIHR) partnership, project number 17/60/22. Standard Protocol Items: Recommendations for Interventional Trials reporting guidelines were used for this protocol.^16^

### Trial setting

The initial aim of the trial was to recruit up to 3062 women to the screening phase with an expected up to 1500 women randomised from 10 tertiary recurrent miscarriage centres. Given the decentralisation of recurrent miscarriage care however, a decision was taken to widen participation allowing trial set-up across all National Health Service (NHS) trusts with a working recurrent miscarriage service. Participants will therefore be recruited from 29 NHS hospital sites located across the UK including England, Scotland and Wales.

### Translational substudy

Women recruited at the sponsor site will be entered into the optional translational substudy. These women will undertake a dual consent process for both the clinical trial and for samples and associated clinical data to be banked within a reproductive health biobank allowing both flexibility and regulatory oversight of additional scientific exploratory work that emerges from endometrial assessment.

Women entered into the translational substudy will undergo sampling of the microbiota in addition to endometrial histological sampling. This will be achieved through the concomitant collection of high vaginal swabs, endocervical swabs and uterine swabs alongside an outpatient endometrial biopsy. Consent to the substudy will be undertaken by the research team at the sponsor site.

Environmental and negative controls will be collected across each collection day to adjust for potential contamination effects. Women who are screen positive will be offered repeat endometrial sampling in the intervention cycle to ascertain the endometrial effects of treatment. Overall, up to 200 paired samples will be obtained. A random sample of high vaginal and cervical swabs will be analysed to ascertain risk of contamination; the remainder will be stored. All endometrial swabs will be analysed.

### Participants and recruitment

Eligibility for inclusion is dependent on the phase of the trial. The key entry point and assessment of eligibility is within the screening phase. These inclusion and exclusion criteria are as below.

#### Inclusion criteria

Women aged ≥18 to <42 who have experienced two or more consecutive first trimester miscarriages, with first trimester miscarriage defined as an intrauterine pregnancy loss at ≤14 weeks gestation. Women who have suffered an ectopic pregnancy, a molar pregnancy or second trimester miscarriage between or after their first trimester miscarriages will still be eligible given the differing pathology of loss.

#### Exclusion criteria

Women will be excluded if they experienced a live birth, stillbirth or termination of pregnancy between or after their first trimester miscarriage. In addition, women with a known treatable cause of RM will be excluded including antiphospholipid syndrome or uncontrolled thyroid disease. Women with a menstrual cycle <21 or >42 days will be excluded due to difficulties in timing the sampling and/or treatment. Those with systemic inflammatory conditions including systemic lupus erythematous and myasthenia gravis will be excluded. Finally, those on long-term antibiotics, having undertaken antibiotic therapy in the screening menstrual cycle or with a contraindication to doxycycline, will be excluded.

### Trial procedures

#### Pre-eligibility phase

Women eligible as above will be advised to avoid pregnancy and provided with ovulation tests. They will be verbally consented to the study by a clinician, trained research nurse or midwife. In a subsequent menstrual cycle, during which any intercourse involved use of barrier contraception, they will undertake ovulation testing and contact the research unit at the point of ovulation to book the screening biopsy.

#### Screening for CE

A medical doctor will confirm eligibility and obtain consent. Women will undergo endometrial screening on day 10±4 following ovulation. Where ovulation testing is inconclusive, screening will be performed on day 23±3. An outpatient endometrial biopsy is undertaken following a negative pregnancy test. Additional samples are undertaken as above in those included within the translational substudy. Samples will be collected by those who do so as part of their job role. Where insufficient samples are obtained the participant has the option for a repeat attempt at collection in the same or next menstrual cycle. Alternatively, they can be referred to the lead site for sample collection. A maximum of two biopsies will be attempted.

Samples will be processed in a central laboratory. A standard protocol for analysis will be followed. A minimum section size of 2 mm^2^ should be present. CD138 immunohistochemistry will be undertaken, and a diagnosis of CE will be made in the presence of≥5 CD138 positive cells per 10 mm^2^ of endometrial section based on the existing literature.^11^ Samples will be reported as positive, or negative, but additional stratification information will be used for analysis. Samples will be stratified into four categories to further aid prognostic analysis: negative (<5 CD138+/10 mm^2^), mild (5–20 CD138+/10 mm^2^), moderate (21–200 CD138+/10 mm^2^) and severe (>200 CD138+/10 mm^2^). In addition, the pattern of CD138 staining will be recorded and the presence of morphological appearance of plasma cells. Borderline samples will be assessed by two trained observers.

#### Additional samples

Microbial samples collected as described previously are immediately placed on ice and transferred to a −80°C freezer. Samples are transferred to a specialist laboratory where RNA extraction will be performed and 16S sequencing conducted. Sequenced data will be passed down a predesigned data pipeline to allow profiling of the microbiota. These will be analysed in conjunction with trial data and CE status. Additionally, remnant tissue at the lead site will be split between 2mls RNA later and stored at −80°C for RNA-related exploratory studies and 4 mL 10% dextran-coated charcoal-treated foetal bovine serum supplemented with DMEM-F12 for culture studies.

#### Randomisation

Following a screen positive result for CE, women are eligible for randomisation provided they remain eligible based on the previous entry criteria, have not subsequently received a course of antibiotics, or had a delay of longer than 3 months since the biopsy was undertaken. Consent is further confirmed by a medical doctor, and randomisation undertaken by a member of the site research team using an online web application hosted by the WCTU. Block randomisation is performed in a 1:1 ratio, with Minimisation by age (<35 vs ≥35 years), number of previous miscarriages (≤3 vs >3) and site.

#### Intervention

Preconceptual doxycycline or placebo overencapsulated for the same appearance will be administered orally at 100 mg two times a day for 14 days. Women are advised to commence the intervention on day 1 of the subsequent menstrual cycle. Those entered into the substudy will undergo ovulation testing within this cycle with repeat biopsy timed as per the screening phase biopsy. Women will be asked to self-record compliance with the intervention.

#### Safety mechanisms

Doxycycline is contraindicated in pregnancy as its use has been reported as teratogenic.^17 18^ Nevertheless, a review by Cross *et al* reported an absence of teratogenic effects and advised the use of doxycycline where clinical circumstances indicate.^19^ Safety mechanisms have, however, been built into the trial to mitigate the risk of inadvertent use in pregnancy. These include the use of a pregnancy test prior to commencement of doxycycline, commencement on the first day of the menstrual period hence ensuring course completion prior to ovulation and finally women are advised to use barrier contraception throughout the remainder of the cycle. Any pregnancies occurring within 4 weeks of the intervention will be reported and thorough assessment performed to assess for evidence of congenital abnormalities. Emergency unblinding is possible via a 24-hour code breaking telephone line. This will be performed for clinical indications.

#### Follow-up

Two separate follow-up pathways exist dependent on CE screen status. Women who screen negative will be followed-up to assess subsequent pregnancy outcome at 3, 6 and 12 months post biopsy or to the first complete pregnancy outcome, whichever comes first. Women who screen positive will be followed up at 3, 6, 12 and 24 months from the point of intervention to the first pregnancy outcome after 24-week gestation, 24 months of follow-up or the end of the trial, whichever comes first.

#### End of trial

Given the adaptive nature of the trial the end can be determined by one of several factors. First, following recruitment and completion of 14 months of subsequent follow-up of 1500 women. Second, where interim analysis demonstrates either efficacy or futility. Third, where mandated by the ethics committee. Fourth, where recruitment falls substantially below target for a period including but not limited to ≤68 women randomised in the first 6 months. Finally, where trial funding ceases.

### Data collection

Pseudoanonymised data attributed to trial ID will be collected and inputted at sites into a dedicated secure trial database. WCTU and UHCW (sponsor) act as joint data controllers for this trial.

Data collected at baseline includes date of birth, ethnicity, body mass index, smoking history, medical history, obstetric/pregnancy history, concomitant medication, pregnancy status, a contraception review and a review of the participant’s menstrual cycle. Further information on the timing of the biopsy and any use of antibiotics in the menstrual cycle will be collected at the screening visit. At the randomisation visit, information on concomitant medication, pregnancy status, use of contraception and adverse events (AEs) will be collected. After the anticipated date of treatment completion, data will be collected on treatment compliance, ongoing contraceptive use and any AEs. Follow-up will occur at 3, 6, 12 and 24 months after randomisation to ascertain pregnancy status. For women who are pregnant, further follow-up will be timed to key pregnancy stages. Pregnancy dating will be confirmed at the booking ultrasound through crown-rump length, viability assessment, concomitant medication review, and any developed pregnancy complications will be noted. Similar information will be captured at ongoing pregnancy reviews. Post pregnancy, details of the pregnancy outcome, placental histology if available, cytogenetics of products of conception if applicable, pregnancy complications, congenital abnormalities, immediate postpartum maternal and infant infections and maternal infections up to 8 weeks post delivery, will be collected. All trial participants are free to withdraw from the trial at any time point. Unless a participant explicitly withdraws consent, they and their infant will be followed up where possible. On withdrawal, no further data will be collected and, if requested, all participant data will be removed.

### Sample size

The total number of women entered for screening will be determined by the CE positivity ratio on screening. A 50% positivity rate has been assumed with a total screen population of 3062 accounting for participant drop out of 2%. This will give a maximum sample size of 1500 randomised with 750 in each arm. The study is therefore powered to detect an 8% between group difference this has been pragmatically selected due to limited previous high-quality work in the area. There are three planned interim analyses after 100, 300 and 900 women have reached 28 weeks post randomisation. These analyses will assess for futility and be reviewed by the DMC. Prespecified stopping rules as documented in the statistical analysis plan will be applied.

### Data analysis

Data will be analysed by intention to treat at randomisation. The treatment difference for the primary outcome will be assessed by deriving the posterior distribution of a Bayesian logistic regression model with a weakly informative prior distribution for all parameters. Although unlikely, in the event of missing data for primary outcomes of>10% imputation techniques will be used. The result for the primary outcome will be reported as the median value of the simulated ORs with the corresponding credible intervals. Secondary outcomes as previously outlined will be similarly assessed. The time-to-event (first pregnancy) analysis will be assessed using semiparametric proportional hazard regression. The following predefined subgroup analyses of the primary outcomes will also be assessed by age (<35 vs ≥35), number of previous miscarriages (≤3 vs >3), severity of CD138 expression and pattern of CD138 expression.

### AE management

AE will be collected to 30 days post-trial intervention, this may be either screening biopsy, treatment completion or secondary biopsy whichever comes later dependent on the participants trial arm. AEs captured elsewhere in the trial data do not require reporting, including those related to pregnancy outcome. In addition, common pregnancy related serious adverse events (SAEs) as seen in box 1 are exempt from SAE reporting but should be reported as AE. A causality assessment will be performed by a doctor for all SAEs and AEs. These will be assessed by the trial management group (TMG) and the DMC. SAEs and AEs thought to be related or unexpected will be reported to the Medicines and Healthcare Products Regulatory Agency) and Research Ethics Committee (REC), the sponsor and the chairs of the Trial Steering Committee (TSC) and DMC.

Box 1 **Adverse events (AEs) exempt from serious adverse event reporting but recorded as AEs**

Admission to hospital for nausea and vomiting

Admission to hospital for headaches

Admission to hospital for raised blood pressure or pregnancy induced hypertension

Admission to hospital for vaginal bleeding

### Monitoring

All clinicians involved in consent should have up to date Good Clinical Practice (GCP) training. Samples may be collected by those who fulfil this role in a clinical capacity. All involved will undergo a relevant programme of training. Site visits will be conducted where concerns arise. Data will be monitored for completeness and quality, where queries arise these will be escalated to the site involved.

A TMG comprising coinvestigators, allied experts, a patient public involvement advisor and the trial management staff will meet monthly to bimonthly to review the day-to-day running of the trial.

A TSC has been established comprising a group of independent experts covering all trial areas and lay members. The TSC will be responsible for protocol approval and review of any protocol changes, advising on aspects of trial conduct, monitoring trial progress and consideration of any recommendations from the DMC. They will meet not less than once a year. Any protocol modifications as agreed by the TSC will be submitted to relevant parties.

A DMC has been established comprising independent experts. They will ensure monitoring of outcomes and safety aspects during the trial.

An authorised representative of the sponsor has approved the final version of the protocol with respect to the trial design, conduct, data analysis and interpretation and plans for publication and dissemination of results.

### Integrated trial design

This trial uses an integrated adaptive approach. This allows facilitation of both assessing the core clinical outcomes but also the development of a concurrent sample and dataset for mechanistic evaluation should an effect be seen. It also allows better understanding of the aetiology of the condition including through the follow-up of those with a negative result. This integrated mechanistic clinical approach forms a model for a more economical approach to trial design in poorly understood conditions. Through more efficient use of resources, it allows the development of a holistic approach to disorder determination. A key hallmark of this design is the flexibility provided by the adaptive design and integration of sample banking. This ensures that a rigorous methodological approach is taken while still facilitating exploratory research. In fields such as this, which face the difficult challenges of a limited evidence base and disorder heterogeneity such approaches are likely to prove increasingly necessary.

### Patient and public involvement

The trial has been designed with the involvement of the Lily Mae Foundation, a charity supporting women who have lost a baby through miscarriage, stillbirth, neonatal death or medical termination. Amy Jackson, cofounder of the charity, sits on the TMG. She has additionally led on the development of patient-facing materials.

### Alterations to study design since commencement

Initial establishment of the trial was on the basis of coprimary outcomes: ongoing pregnancy at 12 weeks at the end of the trial and livebirth ≥24 + 0 weeks gestation at the end of the trial, in the first or subsequent pregnancy. Like other clinical trials, the timeframe for recruitment has meant the trial has been heavily impacted by the COVID-19 pandemic. This has resulted in under-recruitment, with a lower-than-expected trial enrolment size falling short of the targeted 3062 screened and 1500 randomised. In view of this, the TSC unanimously agreed on 22nd November 2022 that, given a likely smaller than anticipated participant population, reduction to a single primary outcome of livebirth ≥24 + 0 weeks gestation at the end of the trial, in the first or subsequent pregnancy, was necessary to ensure statistical robustness. A formal protocol and REC amendment was made, and the change was approved by both the TSC and chair of the DMC.

Also because of the COVID-19 pandemic, a decision was made to allow consent to randomisation to be undertaken over the telephone (this previously required written consent). This was enacted to reduce hospital site visits and a REC amendment made.

## Ethics and dissemination

The trial will be conducted in full conformance with the principles of the Declaration of Helsinki, International Council for Harmonisation GCP guidelines and the UK Statutory Instrument Number 1031 that implements the Medicine for Human Use (Clinical Trials) Directive 2004 and subsequent amendments. Ethical approval for the trial has been obtained from the NHS REC Northwest-Haydock (REC reference: 19/NW/0462, REC approval date: 20/08/2019). The key ethical consideration of the trial revolves around the paradox of asking women who are eager to achieve successful pregnancy to avoid pregnancy for 3 months while undergoing trial procedures. To mitigate this, trial procedures will be kept to as short a period as possible. It is hoped the potential for gain minimises this paradox. All participants will provide written informed consent including for the biobanking of samples at the point of the screening biopsy. Telephone consent will subsequently be taken at the point of randomisation. The consent form is included within the online supplemental material.

10.1136/bmjopen-2023-081470.supp1 Supplementary data

The trial will be reported in accordance with the Consolidated Standards of Reporting Trials guidelines.^20^ The trial will be published in an open-access peer reviewed journal with authorship agreed as per ICMJE requirements.^21^ The full protocol and trial findings will also be reported in the NIHR journals library. In addition, dissemination at national and international specialist conferences will be undertaken. Exploratory outcomes will be disseminated separately in open-access peer-reviewed journals to accommodate their more specialist nature.

### Trial progress to date

This trial opened for recruitment on 19 December 2019 and closed to new registrations on 30 September 2022. The trial is currently in the follow-up period, and the study is expected to be completed by 30 April 2024.

## Supplementary Material

Reviewer comments

Author's manuscript
