# Supplementary material for: Preconceptual administration of doxycycline in women with recurrent miscarriage and chronic endometritis: protocol for the Chronic Endometritis and Recurrent Miscarriage (CERM) trial, a multicentre, double-blind, placebo-controlled, adaptive randomised trial with an embedded translational substudy
Source: BMJ Open. 2023 Dec 1;13(12):e081470. doi: 10.1136/bmjopen-2023-081470 (PMC10693855; doi:10.1136/bmjopen-2023-081470)
Supplement: Supplementary data [file bmjopen-2023-081470supp001.pdf]

[Print on Trust Headed Paper]

Crystal Mark 23135 Clarity approved by Plain English Campaign

Participant information sheet (CERM A)

Information for women taking part in the chronic endometritis and recurrent miscarriage (CERM) trial

Trial title

Chronic endometritis and recurrent miscarriage – the CERM trial

This information sheet is available in **large print** from the university trial team (email: cerm@warwick.ac.uk).

Introduction

You are invited to take part in a research trial. Please read this information sheet carefully before deciding whether to take part. It explains why this research trial is being done and what it means for you if you decide to take part. You will have the opportunity to talk to a member of the hospital research team about this trial and they will be happy to answer any questions you have. You can also discuss the trial with your GP or the obstetrician or gynaecologist caring for you.

This leaflet is divided into two parts. **Part 1** tells you the purpose of the trial and what will happen to you if you take part. **Part 2** gives you more detailed information about how the trial will be carried out.

Part 1

What is the purpose of the research trial?

Inflammation of the lining of the womb is a condition called **endometritis.**

The aim of this research trial is to find out if antibiotics can reduce miscarriage. In some women the lining of the womb (the endometrium) is inflamed. Researchers have found a link between this and miscarriage. A healthy endometrium is important for the embryo to be able to attach to the womb. It is thought that endometritis disrupts this process, and can lead to a miscarriage. Treating endometritis with antibiotics may reduce the inflammation and the likelihood of a miscarriage. This has not been tested. This research trial will test this theory by comparing a 14-day course of an antibiotic (doxycycline) against a placebo (a 'dummy treatment' which will look exactly the same as the antibiotic but contains no active ingredients) to find out if taking antibiotics reduces miscarriages. The trial will be 'double blind'. This means that the women and the trial researchers will not know who is taking the antibiotic capsules and who is taking the placebo. It will also be a randomised controlled trial. This means, if you decide to take part, which capsules you receive (the antibiotic or the placebo) will be decided by chance. The trial will take place in NHS hospitals in the United Kingdom and will involve over 3,000 women who have recurrent miscarriage.

Recurrent miscarriage means two or more miscarriages in a row.

Odendaal J, *et al. BMJ Open* 2023; 13:e081470. doi: 10.1136/bmjopen-2023-081470

CERM Chronic Endometritis and Recurrent Miscarriage

[**Print on Trust Headed Paper**]

Not everyone who has recurrent miscarriage has endometritis, so before you can take part in the randomised controlled trial the hospital trial team will need to take an endometrial biopsy (a small sample of tissue from your endometrium) and examine it under a microscope to find out if you do. We (the university research team) estimate that half the women tested will have endometritis and so be able to take part in the randomised controlled trial.

Researchers also suspect that inflammation of the endometrium may be caused by an imbalance of the microbiome that lives in the reproductive tract (the vagina, cervix, womb, fallopian tubes and ovaries). Another part of the trial is to look at the endometrium and microbiome to see how antibiotics affect these. A diagram of this research trial is shown in figure 1.

The **microbiome** is the name given to all the microbes that live in and on our bodies. It is mostly made up of bacteria, and everyone's microbiome is unique.

CERM
Chronic Endometritis and Recurrent Miscarriage

[Print on Trust Headed Paper]

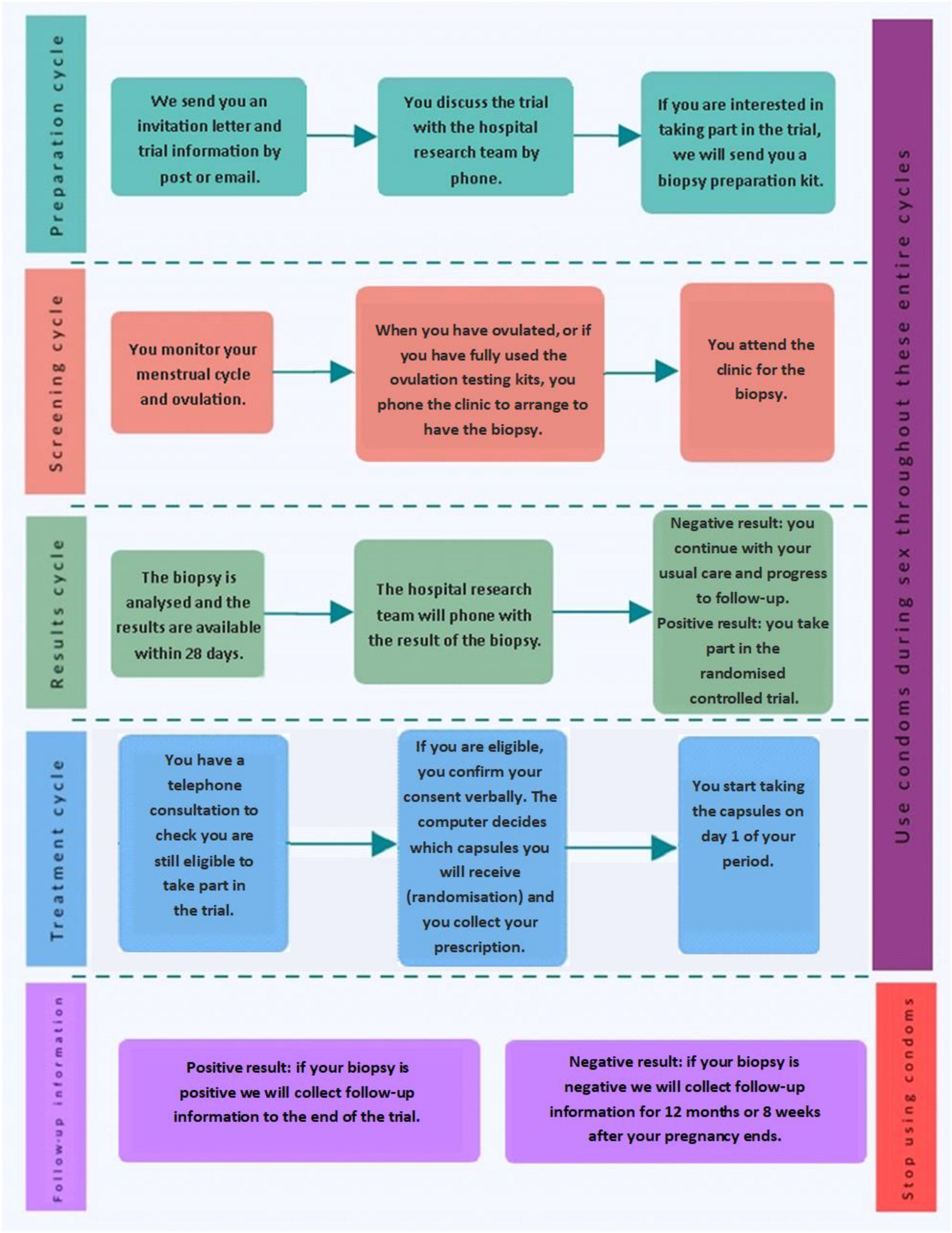

Figure 1 – an overview of the CERM trial

CERM A PIS – Version 3.0 | 05/05/2022 | IRAS ID: 251756 | EudraCT 2019-000585-38 | FINAL Page **3** of **14**

CERM Chronic Endometritis and Recurrent Miscarriage

[Print on Trust Headed Paper]

Screening trial

Why have I been invited?

You have been invited to take part in this research trial because you have had two or more miscarriages (recurrent miscarriage).

Do I have to take part?

Taking part in the research trial is entirely voluntary. It is up to you whether or not you take part. You do not have to take part, and there will be no difference in the care you receive if you choose not to take part. If you want to take part, you will be asked to sign a consent form to confirm you have agreed to take part. You will keep a signed copy of the form. Even after signing the consent form, you can withdraw from the trial at any time if you change your mind, without having to give a reason. This will not affect the care you receive.

This information sheet will now explain what will happen if you decide to take part in this trial.

What will happen next?

A member of the hospital research team will talk to you over the phone (or speak to you face-to-face if you prefer this and if it is possible) to explain what the trial is about and what taking part would mean for you. You can ask the hospital research team any questions you have.

What will happen if I agree to take part?

1. **We will check to see if you are eligible –** You must be aged 18 to 41 years old. The hospital research team will ask about your obstetric and medical history to see if you are eligible to take part in the research trial. If you have not had any investigations to find out the cause of your recurrent miscarriage, these will be done when you attend the clinic. If a cause for your miscarriages is found, you will not be eligible for the trial. An important part of the eligibility check is to find out if you are willing to use condoms whenever you have sex throughout the entire menstrual cycles when you are:
    - preparing for your biopsy;
    - waiting for the result of your biopsy; and
    - taking the capsules.

   Doxycycline, the antibiotic used in the study, can be passed on in breast milk. The effects this could have on babies are not known. For this reason, and to make sure the study is absolutely safe, you will not be able to join the trial if you are breastfeeding.

2. **We will ask for permission to send you a 'biopsy preparation kit' –** If you are eligible to have a biopsy taken, the hospital research team will ask for your verbal consent to post a biopsy preparation kit to you. If you agree, this will be recorded in your hospital case notes and you will be given a trial screening number. The kit contains instructions, a period tracker, condoms and an ovulation testing kit. If you are prescribed a course of antibiotics between giving verbal consent and receiving the biopsy preparation kit, or

Odendaal J, et al. BMJ Open 2023; 13:e081470. doi: 10.1136/bmjopen-2023-081470

CERM Chronic Endometritis and Recurrent Miscarriage

[Print on Trust Headed Paper]

while you are using the biopsy preparation kit, please contact a member of the hospital research team (see the section: How can I contact the hospital trial team?). The biopsy may need to be delayed and the hospital team may need to send you another biopsy preparation kit once you have finished your course of antibiotics.

3. **Preparing for the biopsy –** It is very important that you **do not** try to conceive and that you use condoms whenever you have sex throughout this entire menstrual cycle. Condoms are provided in the kit and you can get more by contacting the hospital research team or family-planning services.

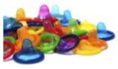

Starting on the first day of your next menstrual cycle after receiving the kit, you should use the period tracker to record:
- the days of your period; and
- the results of the ovulation testing.

The biopsy needs to be taken a few days after you have ovulated. Ovulation usually happens around day 12 to 16, depending on the length of your cycle. We provide a period tracker and ovulation testing kit to help you work out the best time to have the biopsy taken. When the ovulation test shows you have ovulated, or if you have used all of the kit and it does not show you have ovulated, you will need to contact the hospital research team to arrange an appointment to come in for your biopsy.

4. **Having the biopsy –** Please bring your period tracker with you when you come to the clinic for your biopsy. In the clinic a doctor will explain what will happen and check you are eligible to take part in the trial. You will have the opportunity to ask them any questions you might have. You are welcome to bring your partner, a family member, or a friend to this appointment, if the local rules on visitors to the clinic allow this. If you are eligible and would like to take part, you will be asked to sign a consent form. You will also be asked to take a pregnancy test before the biopsy. A healthcare professional will ask you for a sample of urine and they will test it. If the test shows you are pregnant, the biopsy will be cancelled because it could put the pregnancy at risk, and you will be referred to your GP who will arrange for your care. If the pregnancy test shows you are not pregnant, you will register for the trial and be given a trial identification (ID) number. A doctor will then explain the endometrial biopsy procedure.

Before the procedure you will have a vaginal examination to find out the position of your womb so the healthcare professional can take the biopsy. The biopsy usually takes a couple of minutes. The healthcare professional will take the biopsy by passing a thin plastic tube through your cervix and into your womb. We will take a small sample of the lining of your womb. We will send part of this sample to the laboratory at the University Hospitals Coventry and Warwickshire NHS Trust (UHCW) to be analysed under the microscope to find out whether or not you have endometritis. With your permission, any tissue that is left over following analysis, and clinical data that does not identify you will be stored in Tommy's National Reproductive Health Biobank and used for future ethically approved research. This will not affect the sample sent to UHCW for analysis. The sample

Odendaal J, et al. BMJ Open 2023; 13:e081470. doi: 10.1136/bmjopen-2023-081470

sent to UHCW for analysis will be labelled with your trial ID number and initials, not your name.

The healthcare professional will do all they can to make sure they collect enough endometrial tissue to be analysed, but in a few cases this may not be possible. If the healthcare professional is not able to collect a sample or cannot collect enough tissue to be analysed, depending on the reason for this, the health professional carrying out the biopsy will discuss the following options with you.

- The same healthcare professional will repeat the biopsy during the same appointment
- A different healthcare professional will take the biopsy during the same appointment
- You come back to the clinic for another biopsy during your next menstrual cycle
- You have a biopsy taken at UHCW if you are happy and willing to travel to this site
- You choose not to repeat the biopsy and withdraw from the trial

After the biopsy you will be given a pregnancy test kit to take home.

5. **You get your biopsy results and the next steps** – While you are waiting for your biopsy results, it is important that you continue to use condoms whenever you have sex. We will phone you when your biopsy results are available. This will usually be within four weeks.

   If your results show that you do not have endometritis, you will continue with your usual care.
   - We will phone you at three, six and 12 months to see if you are still trying to get pregnant, if you are pregnant or if you have had any miscarriages or terminations. If you get pregnant between our phone calls, please let the hospital research team know (see the section: How can I contact the hospital trial team?).
   - If you get pregnant, we will collect follow-up information from the scans you have during your pregnancy.
   - If you have a baby, we will phone you eight weeks after the birth to ask about your health and your baby's health. The details we ask for will include the date of the birth, how many weeks pregnant you were at the time of the birth, how your baby was delivered, and your baby's weight and sex.
   - We will ask for details of any complications and abnormalities (for both you and your baby).

If your results show that you have endometritis, you may be eligible to take part in the randomised controlled trial. The following section explains what will happen during this stage. It is very important that you use condoms whenever you have sex during this entire menstrual cycle, and not just when you are taking the capsules.

CERM Chronic Endometritis and Recurrent Miscarriage

[Print on Trust Headed Paper]

Randomised controlled trial

A member of the hospital research team will phone you to explain that your results show you have endometritis and you may be eligible to take part in the randomised controlled trial. They will explain what this part of the trial is about and what taking part would mean for you. You can ask the hospital research team any questions you have.

What will happen if I agree to take part?

1. **We will check to see if you are still eligible.** We will make an appointment for you to have a consultation with a member of the hospital research team. The consultation may take place face-to-face or by phone or video call. You can ask any questions you have and you can ask your partner, a family member or a friend to join the consultation.

   During your consultation a doctor will ask you about your medical and obstetric history to check you are eligible to take part in the randomised controlled trial. If investigations have revealed a treatable cause for your recurrent miscarriage since you had the endometrial biopsy, you will not be eligible for the trial and a doctor will discuss your treatment options with you.

   An important part of the eligibility check is to find out if you are still willing to use condoms whenever you have sex throughout your entire menstrual cycle when you are taking the capsules.

   You will be asked to do a pregnancy test (using the pregnancy test kit provided at your biopsy visit) **and** call the hospital research team with the result. If the test shows you are pregnant, you will not be eligible for the trial and you will be referred to your GP who will arrange your care.

   Following the eligibility check, the doctor will ask you to confirm that you are happy to take part in the randomised controlled trial. You are free to withdraw from the trial at this stage or at any point, for any reason.

2. **Randomisation –** Whether you will receive doxycycline or the placebo is decided by chance. You and the trial researchers will not know who is taking the doxycycline and who is taking the placebo, but it will be possible to find this out if it becomes necessary for your clinical care.

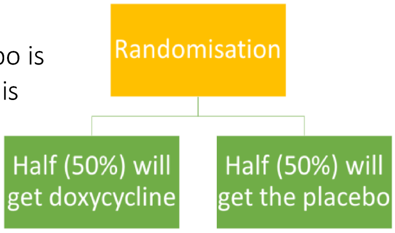

3. **Collecting your prescription and taking the capsules –** You can collect the capsules from a member of the hospital research team or from the hospital pharmacy (after you have collected your prescription from the hospital research team) at an agreed time that is convenient to you.

Odendaal J, *et al. BMJ Open* 2023; 13:e081470. doi: 10.1136/bmjopen-2023-081470

CERM Chronic Endometritis and Recurrent Miscarriage

[Print on Trust Headed Paper**]**

You will be given an information booklet with the capsules. The booklet gives full instructions on how to take the capsules and you should read it carefully. It has guidance on what to do if something happens during the trial, and what precautions you should take while you are taking the capsules.

You can start taking the capsules on the first day of your next menstrual cycle. The dose is one capsule twice a day, 12 hours apart, for 14 days. Please fill in the treatment diary and mark a cross in the diary every time you take a capsule.

These capsules contain gelatine and small amounts of lactose.

4. You will be given a CERM trial participant card. It is very important that you carry your trial participant card with you at all times while you are taking the capsules, and show it to anyone you are receiving healthcare from. Throughout this entire menstrual cycle, it is important that you use condoms whenever you have sex.

5. **If you miss your next period or suspect you are pregnant** - Take a home pregnancy test. Follow the instructions that come with the test. Contact the hospital research team if the pregnancy test is positive or if you are not sure of the result.

Follow-up information

We will only collect information about you and your baby that is relevant to taking part in the CERM research trial. We will ask you for this information, but if you are not sure of, or don't know, the details we need we will refer to your hospital records (at whichever hospital you receive care from during the trial) or GP records (or both).

- We will phone you after you have taken all of your capsules to see if you have taken all of them as prescribed and if you have had any side effects. Please refer to your treatment diary for this information.
- We will phone you at three, six, 12 and 24 months plus at the end of the trial to see if you are still trying to get pregnant, if you are pregnant or if you have had any miscarriages or terminations. If you get pregnant between our phone calls, please let the hospital research team know (see the section: How can I contact the hospital trial team?).
- If you get pregnant, we will collect follow-up information from the scans you have during your pregnancy.
- If you have a baby, we will phone you eight weeks after the birth to ask about your health and your baby's health. The details we ask for will include the date of the birth, how many weeks pregnant you were at the time of the birth, how your baby was delivered, and your baby's weight and sex.
- We will ask for details of any complications, abnormalities, infections, investigations and results, treatment and hospital stays (for both you and your baby).

Expenses and payments

Odendaal J, *et al. BMJ Open* 2023; 13:e081470. doi: 10.1136/bmjopen-2023-081470

CERM Chronic Endometritis and Recurrent Miscarriage

[Print on Trust Headed Paper]

There are no payments for taking part in this research trial. We will refund any hospital parking charges you have to pay for hospital appointments that relate to this research.

What are the clinical alternatives?
Currently there are no clinical alternatives, as few treatments have been shown to prevent miscarriage.

What are the possible disadvantages and risks of taking part?
You and your partner will need to delay trying to conceive because it is important that you use condoms whenever you have sex throughout your entire menstrual cycles when you are:

- preparing for your biopsy;
- waiting for the result of your biopsy; and
- taking the capsules.

We have streamlined the trial processes to make sure the time you need to delay trying to conceive is as short as possible.

Some women find the endometrial biopsy is painful and may get cramping at the time of the biopsy. Taking paracetamol and ibuprofen an hour before the biopsy can help with this. If you need it, gas and air (Entonox) will be available while you are having the biopsy. Some women may have some vaginal bleeding (spotting) after the biopsy is taken. This will stop quickly on its own.

Please see the 'Taking my capsules on the chronic endometritis and recurrent miscarriage (CERM) trial' leaflet for details of any possible side effects while you are taking the capsules, and what to do if something happens when you are taking them.

What are the possible benefits of taking part?
We do not know if taking part in the research trial would benefit you personally. Taking part will show whether you have chronic endometritis. The results will help us provide advice on treatment options for women who have recurrent miscarriage.

What happens when the research trial stops?
The research trial is planned to take up to 48 months to complete.

Whenever the research trial stops, the university trial team will analyse the information collected to decide if antibiotics should be prescribed to women with chronic endometritis. In the future, these results will help women who are experiencing recurrent miscarriage.

This is the end of Part 1.

If you are interested in taking part in the trial, please read the important information in Part 2 before making your decision.

Odendaal J, *et al. BMJ Open* 2023; 13:e081470. doi: 10.1136/bmjopen-2023-081470

[Print on Trust Headed Paper]

Part 2

What if there is a problem?
If you have any concerns about any aspect of this study, you should ask to speak to the researchers who will do their best to answer your questions. If you are still not happy and want to make a complaint, you can do this through the NHS complaints procedure.

Please write to:

Complaints Manager
University Hospitals of Coventry and Warwickshire NHS Trust
Clifford Bridge Road
CV2 2DX

Or phone: 02476 965 198

In the unlikely event that you are harmed by taking part in this study, compensation may be available. If you suspect that the harm is the result of someone's negligence, you may be able to take legal action, but you may have to pay any costs involved and you should get legal advice about this.

For independent advice on research, you can contact PALS (Patient Advice and Liaison Service) on Freephone 0800 028 4203, or you can email them at feedback@uhcw.nhs.uk.

What Covid-19 precautions should I take when coming to hospital for the trial?
Your local NHS trust may have introduced measures to reduce your risk of being exposed to Covid-19. Please see the relevant hospital's website or ask the hospital trial team for more information.

Should I have the Covid-19 vaccine while I am taking part in the trial?
The CERM trial team, which includes clinicians and a pharmacist, have confirmed that there are no interactions between the drug used in the trial (doxycycline) and available Covid-19 vaccines. This means there are no safety concerns with you having a Covid-19 vaccine if you are offered one while you are taking part in the trial.

The hospital research team will ask you to let them know if you do have a Covid-19 vaccine so they can record the details alongside any other medications you are taking. If you have any questions, please get in touch with the hospital research team or your GP.

How will my information be used?
University Hospital Coventry and Warwickshire (UHCW) is the sponsor for this trial in the United Kingdom. The trial will be managed by Warwick Clinical Trials Unit at the University of Warwick (UoW). UHCW and the UoW will use information you provide and information from your hospital records and your GP records to carry out this trial, and will act as joint data

Odendaal J, et al. BMJ Open 2023; 13:e081470. doi: 10.1136/bmjopen-2023-081470

controllers for the trial. This means that, together, they are responsible for looking after your information and using it properly.

We will only collect information about you and your baby that is relevant to taking part in the CERM research trial. We will ask you for this information, but if you are not sure of, or don't know, the details we need we will refer to your hospital records (at whichever hospital you receive care from during the trial) or GP records (or both). The information we will collect is listed below.

- The name of any investigations you have had
- The results of any investigations you have had
- The name of any illnesses, conditions or dependencies you have or have had
- The name of any prescription medications you are taking, the dose or units, and how long you have been taking them
- Details of any pregnancies, miscarriages or terminations
- The results of any pregnancy scans
- Details of any births, including the date, how many weeks pregnant you were at the time of the birth, how your baby was delivered, whether your baby was born alive or was stillborn, and your baby's weight and sex
- Details of any complications, abnormalities, infections, investigations and results, treatment and hospital stays (for both you and your baby)
- The results from the routine genetic analysis of any miscarriage tissue and tissue from the placenta (we will only collect this from you if you choose to take part in the randomised controlled trial)

Your hospital will collect information from you and your medical records and will pass this to us for this research trial in line with our instructions. This information will include your initials and date of birth. We would also like to collect details of your ethnic background and race, but you can choose not to provide this information if you prefer. We will ask for your permission to tell your GP that you are taking part in the research trial. If you do not want us to tell your GP, you will not be able to take part.

Your hospital will keep your name, NHS number and contact details confidential and will not pass this information to UHCW or the UoW. Your hospital will use this information, as needed, to contact you about the research trial, to make sure that relevant information about the trial is recorded for your care, and to oversee the quality of the trial. Certain people from UHCW, the UoW and regulatory organisations may look at your medical and research records to check that the research trial is accurate. UHCW and the UoW will only receive information that does not contain any details that directly identify you. The people who analyse the information will not be able to identify you and will not be able to find out your name, NHS number or contact details. UHCW and the UoW will keep identifiable information about you for 10 years after the trial has finished (if the results of your biopsy show that you **do not** have endometritis) or for 25 years after the trial has finished (if the results of your biopsy show that you **do** have endometritis).

CERM Chronic Endometritis and Recurrent Miscarriage

[Print on Trust Headed Paper]

When you agree to take part in a research trial, the information about your health and care may be provided to researchers running other research studies in this organisation and in other organisations. These organisations may be universities, NHS organisations or companies involved in health and care research in this country or abroad. Organisations and researchers will only use your information to carry out research in line with the UK Policy Framework for Health and Social Care Research. This information will not identify you and will not be combined with other information in a way that could identify you. The information will only be used for the purpose of health and care research. It will not affect your care, and organisations and researchers cannot use it to contact you. Your information will not be used to make decisions about future services that are available to you, such as insurance.

Your rights to see, change or move your information are limited, as your information is managed in specific ways to make sure the research is reliable and accurate. If you withdraw from the trial, the information that has already been collected about you will be kept. To protect your rights, as few details as possible that could identify you will be collected.

To find out more about how your information is handled, you can visit the privacy notices of the data controllers.
www.uhcw.nhs.uk/privacy/
www.warwick.ac.uk/services/idc/dataprotection/privacynotices/researchprivacynotice

Will my information be kept confidential?
Yes. All information collected about you is strictly confidential. Any research information UHCW and the UoW collect will refer to you only by a unique trial ID number and your initials, so the risk of you being identified is very low. All your information will be stored securely and held at the Warwick Clinical Trials Unit, in line with all relevant UK laws.

What if new information becomes available?
If any new information about your treatment becomes available during the research trial, your doctor will discuss this with you.

What will happen if I don't want to carry on with the research trial?
If you do not want to continue in the research trial, you can withdraw at any time without giving a reason. This will not affect your care in any way. If you decide to withdraw from the research trial, you can choose to have no further contact from us.

What will happen to the results of this research trial?
At the end of the research trial, the university trial team will prepare and publish a report. The results of the research trial will be publicly available on the CERM trial website at www.warwick.ac.uk/cerm. The results will be available to the hospitals that took part in the research trial.

The results of the research trial may be presented at scientific meetings and published in scientific journals. The university trial team will also share the results of the research trial with the Royal College of Obstetricians and Gynaecologists, who publish guidance on the best care

for women across the world. You will not be identified in any reports or publications and none of the information will be able to be traced to you personally.

Who is organising and funding this research trial?
This research trial is funded by the National Institute for Health Research (NIHR) in partnership with the Medical Research Council (MRC) under the Efficacy and Mechanism Evaluation Programme 17/60/22. The Government set up the NIHR in 2006 to provide organised funding for research within the NHS.

University Hospital Coventry and Warwickshire is sponsoring the research trial. This covers the insurance costs that apply to research trials. Professor Siobhan Quenby (from University Hospital Coventry, Warwickshire NHS Trust and The University of Warwick) is the Chief Investigator and has overall responsibility for the research trial. The University of Warwick Clinical Trials Unit is organising the administration of the research trial.

Who has reviewed this research trial?

1. Reviewed and commissioned by the National Institute for Health Research - Efficacy and Mechanism Evaluation Programme.
2. Reviewed and approved by North West-Haydock Research Ethics Committee (the REC) on 20/08/2019. The REC are an independent group of people who review all research carried out in the NHS to protect your safety, rights, well-being and dignity.
3. Reviewed and authorised by the Medicines and Healthcare products Regulatory Agency (MHRA) on 20/08/2019. The MHRA is the government body that authorises clinical trials of medicines.
4. Reviewed and approved by the Quality Assurance team at Warwick Clinical Trials Unit.
5. Reviewed and approved by the Research and Development Office at your local NHS trust.
6. Reviewed by the patient and public involvement (PPI) representative on this trial.

How can I contact the hospital trial team?
If you have any questions about the research trial or your involvement in it, either now or in the future, you can contact the hospital trial team.

Hospital trial team
Email: [insert number]
Phone: [insert number]
Write to: [insert number]

Information about the CERM trial, and other useful information, is available from the CERM trial website at www.warwick.ac.uk/cerm.

Thank you for taking the time to read this information sheet.

[Print on Trust Headed Paper**]**

DoH Disclaimer and **Funding acknowledgment** - This project (project reference 17/60/22) is funded by the Efficacy and Mechanism Evaluation (EME) Programme, an MRC and NIHR partnership. The views expressed in this publication are those of the authors and not necessarily those of the MRC, NIHR or the Department of Health and Social Care.

CERM Chronic Endometritis and Recurrent Miscarriage

[Print on Trust Headed Paper]

Crystal Mark 23136 Clarity approved by Plain English Campaign

Participant information sheet (CERM B)

Information for women taking part in the chronic endometritis and recurrent miscarriage (CERM) trial

Trial title

Chronic endometritis and recurrent miscarriage – the CERM trial

This information sheet is available in **large print** from the university trial team (email: cerm@warwick.ac.uk).

Introduction

You are invited to take part in a research trial. Please read this information sheet carefully before deciding whether to take part. It explains why this research trial is being done and what it means for you if you decide to take part. You will have the opportunity to talk to a member of the hospital research team about this trial and they will be happy to answer any questions you have. You can also discuss the trial with your GP or the obstetrician or gynaecologist caring for you.

This leaflet is divided into two parts. **Part 1** tells you the purpose of the trial and what will happen to you if you take part. **Part 2** gives you more detailed information about how the trial will be carried out.

Part 1

What is the purpose of the research trial?

The aim of this research trial is to find out if antibiotics can reduce miscarriage. In some women the lining of the womb (the endometrium) is inflamed. Researchers have found a link between this and miscarriage. A healthy endometrium is important for the embryo to be able to attach to the womb. It is thought that endometritis disrupts this process, and can lead to a miscarriage. Treating endometritis with antibiotics may reduce the inflammation and the likelihood of a miscarriage. This has not been tested. This research trial will test this theory by comparing a 14-day course of an antibiotic (doxycycline) against a placebo (a 'dummy treatment' which will look exactly the same as the antibiotic but contains no active ingredients) to find out if taking antibiotics reduces miscarriages. The trial will be 'double blind'. This means that the women and the trial researchers will not know who is taking the antibiotic capsules and who is taking the placebo. It will also be a randomised controlled trial. This means, if you decide to take part, which capsules you receive (the antibiotic or the placebo) will be decided by chance. The trial will take place in NHS hospitals in the United Kingdom and will involve over 3,000 women who have recurrent miscarriage.

> Inflammation of the lining of the womb is a condition called **endometritis.**

> **Recurrent miscarriage** means two or more miscarriages in a row.

[Print on Trust Headed Paper]

Not everyone who has recurrent miscarriage has endometritis, so before you can take part in the randomised controlled trial the hospital trial team will need to take an endometrial biopsy (a small sample of tissue from your endometrium) and examine it under a microscope to find out if you do. We (the university research team) estimate that half the women tested will have endometritis and so be able to take part in the randomised controlled trial.

Researchers also suspect that inflammation of the endometrium may be caused by an imbalance of the microbiome that lives in the reproductive tract (the vagina, cervix, womb, fallopian tubes and ovaries). Another part of the trial is to look at the endometrium and microbiome to see how antibiotics affect these. A diagram of this research trial is shown in figure 1.

The **microbiome** is the name given to all the microbes that live in and on our bodies. It is mostly made up of bacteria, and everyone's microbiome is unique.

Odendaal J, *et al. BMJ Open* 2023; 13:e081470. doi: 10.1136/bmjopen-2023-081470

CERM Chronic Endometritis and Recurrent Miscarriage

[Print on Trust Headed Paper]

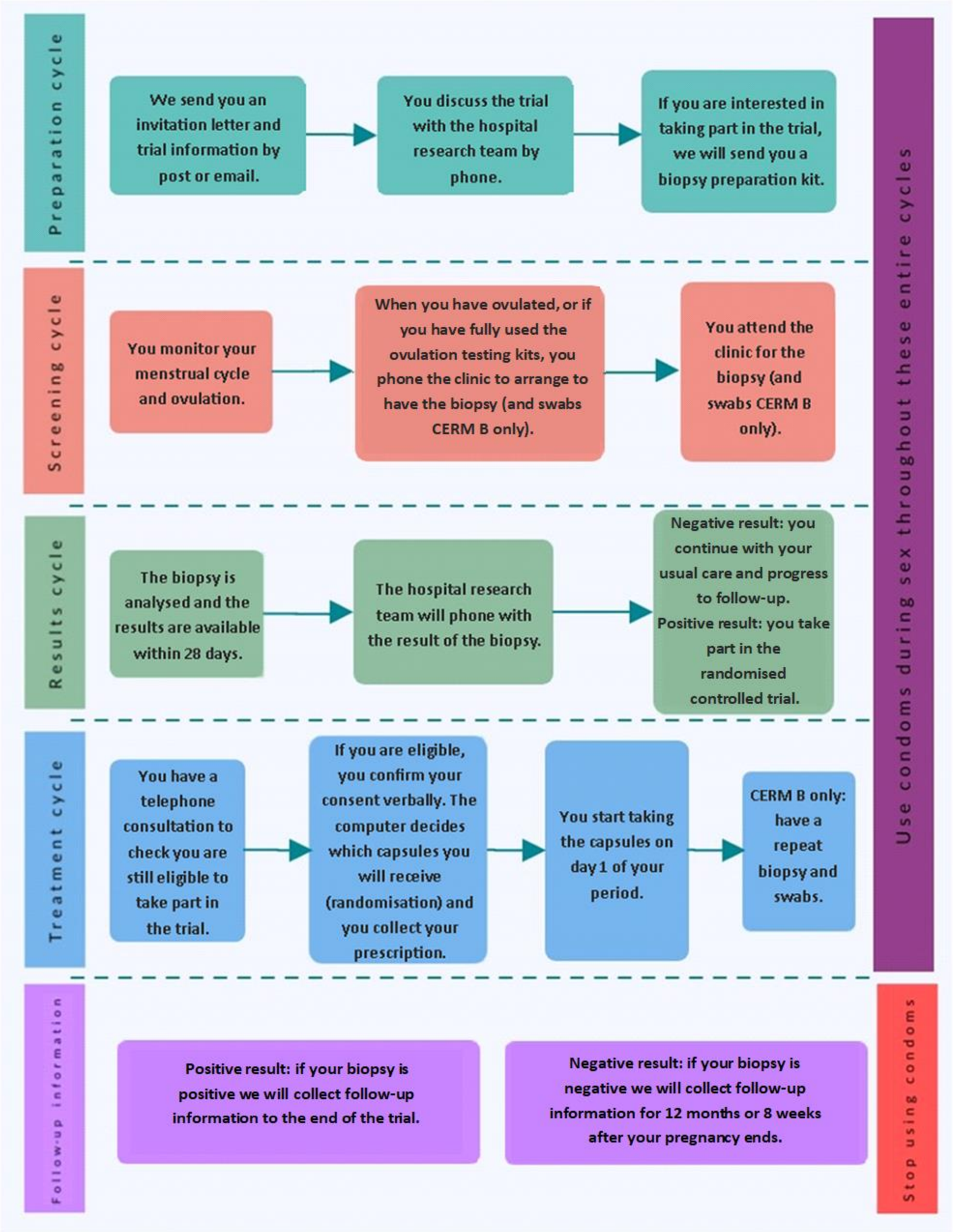

Figure 1 – an overview of the CERM trial

Odendaal J, *et al. BMJ Open* 2023; 13:e081470. doi: 10.1136/bmjopen-2023-081470

CERM Chronic Endometritis and Recurrent Miscarriage

[Print on Trust Headed Paper]

Screening trial

Why have I been invited?

You have been invited to take part in this research trial because you have had two or more miscarriages (recurrent miscarriage).

Do I have to take part?

Taking part in the research trial is entirely voluntary. It is up to you whether or not you take part. You do not have to take part, and there will be no difference in the care you receive if you choose not to take part. If you want to take part, you will be asked to sign a consent form to confirm you have agreed to take part. You will keep a signed copy of the form. Even after signing the consent form, you can withdraw from the trial at any time if you change your mind, without having to give a reason. This will not affect the care you receive.

This information sheet will now explain what will happen if you decide to take part in this trial and have an endometrial biopsy and vaginal, cervical and endometrial swabs taken.

What will happen next?

A member of the hospital research team will talk to you over the phone (or speak to you face-to-face if you prefer this and if it is possible) to explain what the trial is about and what taking part would mean for you. You can ask the hospital research team any questions you have.

What will happen if I agree to take part?

1. **We will check to see if you are eligible –** You must be aged 18 to 41 years old. The hospital research team will ask about your obstetric and medical history to see if you are eligible to take part in the research trial. If you have not had any investigations to find out the cause of your recurrent miscarriage, these will be done when you attend the clinic. If a cause for your miscarriages is found, you will not be eligible for the trial. An important part of the eligibility check is to find out if you are willing to use condoms whenever you have sex throughout the entire menstrual cycles when you are:
   - preparing for your biopsy and swabs;
   - waiting for the result of your biopsy; and
   - taking the capsules and having your repeat biopsy and swabs.

   Doxycycline, the antibiotic used in the study, can be passed on in breast milk. The effects this could have on babies are not known. For this reason, and to make sure the study is absolutely safe, you will not be able to join the trial if you are breastfeeding.

2. **We will ask for permission to send you a 'biopsy preparation kit' –** If you are eligible to have a biopsy taken, the hospital research team will ask for your verbal consent to post a biopsy preparation kit to you. If you agree, this will be recorded in your hospital case notes and you will be given a trial screening number. The kit contains instructions, a period tracker, condoms and an ovulation testing kit. If you are prescribed a course of antibiotics between giving verbal consent and receiving the biopsy preparation kit, or

while you are using the biopsy preparation kit, please contact a member of the hospital research team (see the section: How can I contact the hospital trial team?). The biopsy may need to be delayed and the hospital team may need to send you another biopsy preparation kit once you have finished your course of antibiotics.

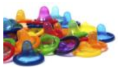

3. **Preparing for the biopsy and swabs –** It is very important that you **do not** try to conceive and that you use condoms whenever you have sex throughout this entire menstrual cycle. Condoms are provided in the kit and you can get more by contacting the hospital research team or family-planning services.

   Starting on the first day of your next menstrual cycle after receiving the kit, you should use the period tracker to record:
   - the days of your period; and
   - the results of the ovulation testing.

   The biopsy and swabs need to be taken a few days after you have ovulated. Ovulation usually happens around day 12 to 16, depending on the length of your cycle. We provide a period tracker and ovulation testing kit to help you work out the best time to have the biopsy and swabs taken. When the ovulation test shows you have ovulated, or if you have used all of the kit and it does not show you have ovulated, you will need to contact the hospital research team to arrange an appointment to come in for your biopsy and swabs.

4. **Having the biopsy and swabs –** Please bring your period tracker with you when you come to the clinic for your biopsy and swabs. In the clinic a doctor will explain what will happen and check you are eligible to take part in the trial. You will have the opportunity to ask them any questions you might have. You are welcome to bring your partner, a family member or a friend to this appointment, if the local rules on visitors to the clinic allow this. If you are eligible and would like to take part, you will be asked to sign a consent form. You will also be asked to take a pregnancy test before the biopsy. A healthcare professional will ask you for a sample of urine and they will test it. If the test shows you are pregnant, the biopsy and swabs will be cancelled, because it could put the pregnancy at risk, and you will be referred to your GP who will arrange for your care. If the pregnancy test shows you are not pregnant, you will register for the trial and be given a trial identification (ID) number. A doctor will then explain the endometrial biopsy procedure.

   Before the procedure you will have a vaginal examination to find out the position of your womb so the healthcare professional can take the biopsy and swabs. The biopsy and swabs usually take a couple of minutes. First the healthcare professional will take swabs from your vagina, cervix and endometrium. They will send the swabs, and clinical data that does not identify you to the laboratory at Imperial College London, who will look at the microbiome to see what microbes are present. With your permission any material that is left over from the swabs, and clinical data that does not identify you, will be stored in the Tommy's National Reproductive Health Biobank. As this part of the trial is to look at how chronic endometritis may cause miscarriage, you will not get any results from these swabs.

The healthcare professional will then take the biopsy by passing a thin plastic tube through your cervix and into your womb. We will take a small sample of the lining of your womb. We will send part of this sample to the laboratory at the University Hospitals Coventry and Warwickshire NHS Trust (UHCW) to be analysed under the microscope to find out whether or not you have endometritis. The sample sent to UHCW for analysis will be labelled with your trial ID number and initials, not your name. With your permission, any tissue that is left over following the analysis, and clinical data that does not identify you, will be stored in the Tommy's National Reproductive Health Biobank and used in future ethically approved research.

The healthcare professional will do all they can to make sure they collect enough endometrial tissue to be analysed, but in a few cases this may not be possible. If the healthcare professional is not able to collect a sample or cannot collect enough tissue to be analysed, depending on the reason for this, the health professional carrying out the biopsy will discuss the following options with you.

- The same healthcare professional will repeat the biopsy during the same appointment
- A different healthcare professional will take the biopsy during the same appointment
- You come back to the clinic for another biopsy during your next menstrual cycle
- You choose not to repeat the biopsy and withdraw from the trial

After the biopsy you will be given a pregnancy test kit to take home.

5. **You get your biopsy results and the next steps** – While you are waiting for your biopsy results, it is important that you continue to use condoms whenever you have sex. We will phone you when your biopsy results are available. This will usually be within four weeks.

   If your results show that you do not have endometritis, you will continue with your usual care.
   - We will phone you at three, six and 12 months to see if you are still trying to get pregnant, if you are pregnant or if you have had any miscarriages or terminations. If you get pregnant between our phone calls, please let the hospital research team know (see the section: How can I contact the hospital trial team?).
   - If you get pregnant, we will collect follow-up information from the scans you have during your pregnancy.
   - If you have a baby, we will phone you eight weeks after the birth to ask about your health and your baby's health. The details we ask for will include the date of the birth, how many weeks pregnant you were at the time of the birth, how your baby was delivered, and your baby's weight and sex.
   - We will ask for details of any complications and abnormalities (for both you and your baby).

If your results show that you have endometritis, you may be eligible to take part in the randomised controlled trial. The following section explains what will happen during this stage.

CERM Chronic Endometritis and Recurrent Miscarriage

[Print on Trust Headed Paper**]**

It is very important that you use condoms whenever you have sex during this entire menstrual cycle, and not just when you are taking the capsules.

Randomised controlled trial

A member of the hospital research team will phone you to explain that your results show you have endometritis and you may be eligible to take part in the randomised controlled trial. They will explain what this part of the trial is about and what taking part would mean for you. You can ask the hospital research team any questions you have.

What will happen if I agree to take part?

1. **We will check to see if you are still eligible.** We will make an appointment for you to have a consultation with a member of the hospital research team. The consultation may take place face-to-face or by phone or video call. You can ask any questions you have and you can ask your partner, a family member or a friend to join the consultation.

   During your consultation a doctor will ask you about your medical and obstetric history to check you are eligible to take part in the randomised controlled trial. If investigations have revealed a treatable cause for your recurrent miscarriage since you had the endometrial biopsy, you will not be eligible for the trial and a doctor will discuss your treatment options with you.

   An important part of the eligibility check is to find out if you are still willing to use condoms whenever you have sex throughout your entire menstrual cycle when you are taking the capsules.

   You will be asked to do a pregnancy test (using the pregnancy test kit provided at your biopsy visit) **and** call the hospital research team with the result. If the test shows you are pregnant, you will not be eligible for the trial and you will be referred to your GP who will arrange your care.

   Following the eligibility check, the doctor will ask you to confirm that you are happy to take part in the randomised controlled trial. You are free to withdraw from the trial at this stage or any point, for any reason.

2. **Randomisation –** Whether you will receive doxycycline or the placebo is decided by chance. You and the trial researchers will not know who is taking the doxycycline and who is taking the placebo, but it will be possible to find this out if it becomes necessary for your clinical care.

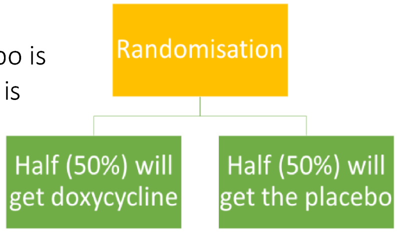

CERM Chronic Endometritis and Recurrent Miscarriage

[Print on Trust Headed Paper]

3. **Collecting your prescription and taking the capsules –** You can collect the capsules from a member of the hospital research team or from the hospital pharmacy (after you have collected your prescription from the hospital research team) at an agreed time that is convenient to you.

   You will be given an information booklet with the capsules. The booklet gives full instructions on how to take the capsules and you should read it carefully. It has guidance on what to do if something happens during the trial, and what precautions you should take while you are taking the capsules.

   You can start taking the capsules on the first day of your next menstrual cycle. The dose is one capsule twice a day, 12 hours apart, for 14 days. Please fill in the treatment diary and mark a cross in the diary every time you take a capsule.

   These capsules contain gelatine and small amounts of lactose.

   You will be given a CERM trial participant card. It is very important that you carry your trial participant card with you at all times while you are taking the capsules, and show it to anyone you are receiving healthcare from. Throughout this entire menstrual cycle, it is important that you use condoms whenever you have sex.

4. **Preparing for the repeat biopsy and swabs –** The procedure is identical to the first biopsy and swabs you had taken, and the instructions are repeated for you below. You can choose not to come for the second biopsy for any reason.

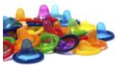

   It is very important that you **do not** try to conceive and you use condoms whenever you have sex throughout the entire menstrual cycle while you are taking the capsules and preparing for your repeat biopsy and swabs. Condoms were provided in the biopsy preparation kit and you can get more by contacting the hospital research team or family-planning services.

   Starting on the first day of your next menstrual cycle you should use the period tracker to record:
   a. the days of your period; and
   b. the results of the ovulation testing.

   The repeat biopsy and swabs need to be taken a few days after you have ovulated. Ovulation usually happens around day 12 to 16, depending on the length of your cycle. We provide a period tracker and ovulation testing kit to help you work out the best time to have the biopsy and swabs taken. When the ovulation test shows you have ovulated, or if you have used all of the kit and it does not show you have ovulated, you will need to contact the hospital research team to arrange an appointment to come in for your biopsy and swabs.

Odendaal J, *et al. BMJ Open* 2023; 13:e081470. doi: 10.1136/bmjopen-2023-081470

CERM Chronic Endometritis and Recurrent Miscarriage **[Print on Trust Headed Paper]**

5. **Having the repeat biopsy and swabs –** Please bring your period tracker with you when you come to the clinic for your repeat biopsy and swabs. In the clinic a member of the hospital research team will explain what will happen. You will have the opportunity to ask them any questions you might have. You are welcome to bring your partner, a family member or a friend to this appointment, if the local rules on visitors to the clinic allow this. You will also be asked to take a pregnancy test. If the test shows you are pregnant, the biopsy and swabs will not be taken and details of your pregnancy will be recorded. If the pregnancy test shows you are not pregnant, a doctor will then explain the endometrial biopsy and swab procedure.

   Before the procedure you will have a vaginal examination to find out the position of your womb so the healthcare professional can take the biopsy and swabs. The biopsy and swabs usually take a couple of minutes. First the healthcare professional will take swabs from your vagina, cervix and endometrium. They will send the swabs, and clinical data that does not identify you, to the laboratory at Imperial College London, who will look at the microbiome to see what microbes are present. With your permission any material that is left over from the swabs, and clinical data that does not identify you, will be stored in the Tommy's National Reproductive Health Biobank.

   The healthcare professional will then take the biopsy by passing a thin plastic tube through your cervix and into your womb. We will take a small sample of the lining of your womb. We will send part of this sample to the laboratory at the University Hospitals Coventry and Warwickshire NHS Trust (UHCW) to be analysed under the microscope. The sample will be labelled with your trial ID number and initials, not your name. With your permission, any tissue that is left over following the analysis and clinical data that does not identify you, will be stored in the Tommy's National Reproductive Health Biobank and used in future ethically approved research. You will not receive the results of the biopsy or swabs, but they will allow us to find out the effect of the capsules.

   The healthcare professional will do all they can to make sure they collect enough endometrial tissue to be analysed, but in a few cases this may not be possible. If the healthcare professional is not able to collect a sample or cannot collect enough tissue to be analysed, the biopsy will not be performed again.

   When you attend for your repeat biopsy and swabs we will ask you if you have taken all of your capsules as prescribed and if you have had any side effects. Please bring your treatment diary and any leftover capsules to this visit.

6. **If you miss your next period or suspect you are pregnant** - Take a home pregnancy test. Follow the instructions that come with the test. Contact the hospital research team if the pregnancy test is positive or if you are unsure of the result.

Follow-up information

We will only collect information about you and your baby that is relevant to taking part in the CERM research trial. We will ask you for this information, but if you are not sure of, or don't

Odendaal J, *et al. BMJ Open* 2023; 13:e081470. doi: 10.1136/bmjopen-2023-081470

know, the details we need we will refer to your hospital records (at whichever hospital you receive care from during the trial) or GP records (or both).

- We will phone you after you have taken all of your capsules to see if you have taken all of them as prescribed and if you have had any side effects. Please refer to your treatment diary for this information.
- We will phone you at three, six, 12 and 24 months plus at the end of the trial to see if you are still trying to get pregnant, if you are pregnant or if you have had any miscarriages or terminations. If you get pregnant between our phone calls, please let the hospital research team know (see the section: How can I contact the hospital trial team?).
- If you get pregnant, we will collect follow-up information from the scans you have during your pregnancy.
- If you have a baby, we will phone you eight weeks after the birth to ask about your health and your baby's health. The details we ask for will include the date of the birth, how many weeks pregnant you were at the time of the birth, how your baby was delivered, and your baby's weight and sex.
- We will ask for details of any complications, abnormalities, infections, investigations and results, treatment and hospital stays (for both you and your baby).

Expenses and payments

There are no payments for taking part in this research trial. We will refund any hospital parking charges you have to pay for hospital appointments that relate to this research.

What are the clinical alternatives?

Currently there are no clinical alternatives, as few treatments have been shown to prevent miscarriage.

What are the possible disadvantages and risks of taking part?

You and your partner will need to delay trying to conceive because it is important that you use condoms whenever you have sex throughout your entire menstrual cycles when you are:

- preparing for your biopsy and swabs
- waiting for the result of your biopsy
- taking the capsules; and
- preparing for your repeat biopsy and swabs.

We have streamlined the trial processes to make sure the time you need to delay trying to conceive is as short as possible.

Some women find the endometrial biopsy is painful and may get cramping at the time of the biopsy. Taking paracetamol and ibuprofen an hour before the biopsy can help with this. If you need it, gas and air (Entonox) will be available while you are having the biopsy. Some women

[Print on Trust Headed Paper]

may have some vaginal bleeding (spotting) after the biopsy is taken. This will stop quickly on its own.

Please see the 'Taking my capsules on the chronic endometritis and recurrent miscarriage (CERM) trial' leaflet for details of any possible side effects while you are taking the capsules, and what to do if something happens when you are taking them.

What are the possible benefits of taking part?
We do not know if taking part in the research trial would benefit you personally. Taking part will show whether you have chronic endometritis. The results will help us provide advice on treatment options for women who have recurrent miscarriage.

What happens when the research trial stops?
The research trial is planned to take up to 48 months to complete.

Whenever the research trial stops, the university trial team will analyse the information collected to decide if antibiotics should be prescribed to women with chronic endometritis. In the future, these results will help women who are experiencing recurrent miscarriage.

This is the end of Part 1.

If you are interested in taking part in the trial, please read the important information in Part 2 before making your decision.

Part 2

What if there is a problem?
If you have any concerns about any aspect of this study, you should ask to speak to the researchers who will do their best to answer your questions. If you are still not happy and want to make a complaint, you can do this through the NHS complaints procedure.

Please write to:

Complaints Manager
University Hospitals of Coventry and Warwickshire NHS Trust
Clifford Bridge Road
CV2 2DX

Or phone: 02476 965 198

In the unlikely event that you are harmed by taking part in this study, compensation may be available. If you suspect that the harm is the result of someone's negligence, you may be able to take legal action, but you may have to pay any costs involved and you should get legal advice about this.

Odendaal J, *et al. BMJ Open* 2023; 13:e081470. doi: 10.1136/bmjopen-2023-081470

CERM Chronic Endometritis and Recurrent Miscarriage

[Print on Trust Headed Paper]

For independent advice on research, you can contact PALS (Patient Advice and Liaison Service) on Freephone 0800 028 4203, or you can email them at feedback@uhcw.nhs.uk.

What Covid-19 precautions should I take when coming to hospital for the trial?
Your local NHS trust may have introduced measures to reduce your risk of being exposed to Covid-19. Please see the relevant hospital's website or ask the hospital trial team for more information.

Should I have the Covid-19 vaccine while I am taking part in the trial?
The CERM trial team, which includes clinicians and a pharmacist, have confirmed that there are no interactions between the drug used in the trial (doxycycline) and available Covid-19 vaccines. This means there are no safety concerns with you having a Covid-19 vaccine if you are offered one while you are taking part in the trial.

The hospital research team will ask you to let them know if you do have a Covid-19 vaccine so they can record the details alongside any other medications you are taking. If you have any questions, please get in touch with the hospital research team or your GP.

How will my information be used?
University Hospitals Coventry and Warwickshire NHS Trust (UHCW) is the sponsor for this trial in the United Kingdom. The trial will be managed by Warwick Clinical Trials Unit at the University of Warwick (UoW). UHCW and the UoW will use information you provide and information from your hospital records and your GP records to carry out this trial, and will act as joint data controllers for the trial. This means that, together, they are responsible for looking after your information and using it properly.

We will only collect information about you and your baby that is relevant to taking part in the CERM research trial. We will ask you for this information, but if you are not sure of, or don't know, the details we need we will refer to your hospital records (at whichever hospital you receive care from during the trial) or GP records (or both). The information we will collect is listed below.

- The name of any investigations you have had
- The results of any investigations you have had
- The name of any illnesses, conditions or dependencies you have or have had
- The name of any prescription medications you are taking, the dose or units, and how long you have been taking them
- Details of any pregnancies, miscarriages or terminations
- The results of any pregnancy scans
- Details of any births, including the date, how many weeks pregnant you were at the time of the birth, how your baby was delivered, whether your baby was born alive or was stillborn, and your baby's weight and sex
- Details of any complications, abnormalities, infections, investigations and results, treatment and hospital stays (for both you and your baby)

Odendaal J, et al. BMJ Open 2023; 13:e081470. doi: 10.1136/bmjopen-2023-081470

- The results from the routine genetic analysis of any miscarriage tissue and tissue from the placenta (we will only record these if you choose to take part in the randomised controlled trial)

Your hospital will collect information from you and your medical records and will pass this to us for this research trial in line with our instructions. This information will include your initials and date of birth. We would also like to collect details of your ethnic background and race, but you can choose not to provide this information if you prefer. We will ask for your permission to tell your GP that you are taking part in the research trial. If you do not want us to tell your GP, you will not be able to take part.

Your hospital will keep your name, NHS number and contact details confidential and will not pass this information to UHCW or the UoW. Your hospital will use this information, as needed, to contact you about the research trial, to make sure that relevant information about the trial is recorded for your care, and to oversee the quality of the trial. Certain people from UHCW, the UoW and regulatory organisations may look at your medical and research records to check that the research trial is accurate. UHCW and the UoW will only receive information that does not contain any details that directly identify you. The people who analyse the information will not be able to identify you and will not be able to find out your name, NHS number or contact details. UHCW and the UoW will keep identifiable information about you for 10 years after the trial has finished (if the results of your biopsy show that you **do not** have endometritis) or for 25 years after the trial has finished (if the results of your biopsy show that you **do** have endometritis).

When you agree to take part in a research trial, the information about your health and care may be provided to researchers running other research studies in this organisation and in other organisations. These organisations may be universities, NHS organisations or companies involved in health and care research in this country or abroad. Organisations and researchers will only use your information to carry out research in line with the UK Policy Framework for Health and Social Care Research. This information will not identify you and will not be combined with other information in a way that could identify you. The information will only be used for the purpose of health and care research. It will not affect your care, and organisations and researchers cannot use it to contact you. Your information will not be used to make decisions about future services that are available to you, such as insurance.

Your rights to see, change or move your information are limited, as your information is managed in specific ways to make sure the research is reliable and accurate. If you withdraw from the trial, the information that has already been collected about you will be kept. To protect your rights, as few details as possible that could identify you will be collected.

To find out more about how your information is handled, you can visit the privacy notices of the data controllers.
www.uhcw.nhs.uk/privacy/
www.warwick.ac.uk/services/idc/dataprotection/privacynotices/researchprivacynotice

Will my information be kept confidential?

CERM Chronic Endometritis and Recurrent Miscarriage

[Print on Trust Headed Paper]

Yes. All information collected about you is strictly confidential. Any research information UHCW and the UoW collect will refer to you only by a unique trial ID number and your initials, so the risk of you being identified is very low. All your information will be stored securely and held at the Warwick Clinical Trials Unit, in line with all relevant UK laws.

What if new information becomes available?
If any new information about your treatment becomes available during the research trial, your doctor will discuss this with you.

What will happen if I don't want to carry on with the research trial?
If you do not want to continue in the research trial, you can withdraw at any time without giving a reason. This will not affect your care in any way. If you decide to withdraw from the research trial, you can choose to have no further contact from us.

What will happen to the results of this research trial?
At the end of the research trial, the university trial team will prepare and publish a report. The results of the research trial will be publicly available on the CERM trial website at www.warwick.ac.uk/cerm. The results will be available to the hospitals that took part in the research trial.

The results of the research trial may be presented at scientific meetings and published in scientific journals. The university trial team will also share the results of the research trial with the Royal College of Obstetricians and Gynaecologists, who publish guidance on the best care for women across the world. You will not be identified in any reports or publications and none of the information will be able to be traced to you personally.

Who is organising and funding this research trial?
This research trial is funded by the National Institute for Health Research (NIHR) in partnership with the Medical Research Council (MRC) under the Efficacy and Mechanism Evaluation Programme 17/60/22. The Government set up the NIHR in 2006 to provide organised funding for research within the NHS.

University Hospital Coventry and Warwickshire is sponsoring the research trial. This covers the insurance costs that apply to research trials. Professor Siobhan Quenby (from University Hospital Coventry, Warwickshire NHS Trust and The University of Warwick) is the Chief Investigator and has overall responsibility for the research trial. The University of Warwick Clinical Trials Unit is organising the administration of the research trial.

Who has reviewed this research trial?

1. Reviewed and commissioned by the National Institute for Health Research - Efficacy and Mechanism Evaluation Programme.
2. Reviewed and approved by North West-Haydock Research Ethics Committee (the REC) on 20/08/2019. The REC are an independent group of people who review all research carried out in the NHS to protect your safety, rights, well-being and dignity.

[Print on Trust Headed Paper]

3. Reviewed and authorised by the Medicines and Healthcare products Regulatory Agency (MHRA) on 20/08/2019. The MHRA is the government body that authorises clinical trials of medicines.
4. Reviewed and approved by the Quality Assurance team at Warwick Clinical Trials Unit.
5. Reviewed and approved by the Research and Development Office at your local NHS trust.
6. Reviewed by the patient and public involvement (PPI) representative on this trial.

How can I contact the hospital trial teams?

If you have any questions about the research trial or your involvement in it, either now or in the future, you can contact the hospital trial team.

Hospital trial team
Email: [insert number]
Phone: [insert number]
Write to: [insert number]

Information about the CERM trial, and other useful information, is available from the CERM trial website at www.warwick.ac.uk/cerm.

Thank you for taking the time to read this information sheet.

DoH Disclaimer and **Funding acknowledgment** - This project (project reference 17/60/22) is funded by the Efficacy and Mechanism Evaluation (EME) Programme, an MRC and NIHR partnership. The views expressed in this publication are those of the authors and not necessarily those of the MRC, NIHR or the Department of Health and Social Care.

CERM Chronic Endometritis and Recurrent Miscarriage

Print on Trust headed paper

Participant consent form (CERM A)

Permission to take part in the chronic endometritis and recurrent miscarriage (CERM) trial

This form is available in **large print** from the trial team. Email **cerm@warwick.ac.uk**

Make three copies: Keep the original in the CERM trial investigator site file, give one copy to the participant, and put one in the participant's hospital medical notes.

Trial ID number					Principal investigator		Site	

**1.** I have read and understood the CERM A participant information sheet version [insert number] dated [dd/mm/yyyy].	Please initial
**2.** I have had time to think about the information I have been given about the CERM trial and ask questions, and I am satisfied with the answers I have been given.	Please initial
**3.** I understand that I do not have to take part and that I can withdraw from the CERM trial at any time, without giving a reason and without my medical care or legal rights being affected.	Please initial
**4.** I understand that the hospital research team will ask me for information about my past and current health that is relevant to me taking part in the CERM research trial. I give the hospital research team permission to collect and record this information.	Please initial
**5.** I understand that the hospital research team will collect information from my NHS hospital and GP records if it is relevant to me taking part in the CERM trial. I give the research team permission to access these records to collect and record this information.	Please initial
**6.** I understand that the research team and trial monitors from Warwick Clinical Trials Unit, the hospital trusts and regulatory authorities may have access to my medical and trial records to monitor this trial. I give permission for these people to access and record this information.	Please initial
**7.** I confirm that my partner and I have used condoms during this menstrual cycle. I will continue to use condoms whenever I am having sex throughout my entire menstrual cycle while I am waiting for the result of my biopsy and, if I take part in the randomised trial, while I am taking the capsules.	Please initial
**8.** I have seen the result of my pregnancy test taken today and confirm that it showed that I am not pregnant.	Please initial
**9.** I agree to have an endometrial biopsy taken.	Please initial
**10.** I agree to my endometrial biopsy being held in the Tommy's National Reproductive Health Biobank until it is analysed.	Please initial
**11. Optional:** I agree that any tissue that is left over following my endometrial biopsy or after my endometrial biopsy is analysed can be stored in the Tommy's National Reproductive Health Biobank, **with clinical data that does not identify me,** and used in future ethically approved research.	Please initial
**12.** I agree to the hospital trial team storing my phone numbers so they can contact me with the results of my endometrial biopsy and to discuss the next steps.	Please initial
**13.** I agree to the hospital trial team storing my phone numbers and contacting me for follow-up information as explained in the participant information sheet (or sheets).	Please initial
**14.** I agree to the research team telling my GP that I am taking part in the CERM research trial.	Please initial
**15.** I agree to take part in the CERM research trial.	Please initial

Please go to page 2.

Crystal Mark 23120 Clarity approved by Plain English Campaign

CERM
Chronic Endometritis and Recurrent Miscarriage

Print on Trust headed paper

We will only use the following consent if you later take part in the randomised controlled trial.

**16.** I understand that I should not take doxycycline while I am pregnant because it can affect the baby. I understand it is important to use condoms during my entire menstrual cycle when I am taking doxycycline or a placebo (whichever I am given).	Please initial
**17.** I agree to take doxycycline or a placebo for 14 days and only as instructed.	Please initial
**18.** I understand that if I give birth at another hospital, the hospital research team will contact the relevant hospital trust to collect medical details from my NHS hospital records (and my baby's) if this is relevant to taking part in the CERM research trial. I give the research team permission to access these records to collect and record this information.	Please initial
**19.** I understand that the hospital research team will collect information from my baby's NHS hospital and GP records if it is relevant to me taking part in the CERM trial. I give the research team permission to access these records to collect and record this information.	Please initial
**20.** I understand that the research team and trial monitors from Warwick Clinical Trials Unit, the hospital trusts and regulatory authorities may have access to my baby's medical and trial records to monitor this trial. I give permission for these people to access and record this information.	Please initial
**21.** I agree to take part in the CERM randomised controlled trial.	Please initial

Your name Please print **Your signature** Please sign **Date** dd/mm/yyyy

Investigator's name Please print **Investigator's signature** Please sign **Date** dd/mm/yyyy

Crystal Mark 23120 Clarity approved by Plain English Campaign

CERM Chronic Endometritis and Recurrent Miscarriage

Print on Trust headed paper

Participant consent form (CERM B)

Permission to take part in the chronic endometritis and recurrent miscarriage (CERM) trial

This form is available in **large print** from the trial team. Email **cerm@warwick.ac.uk**

Make three copies: Keep the original in the CERM trial investigator site file, give one copy to the participant, and put one in the participant's hospital medical notes.

Trial ID number					Principal investigator		Site	

**1.** I have read and understood the CERM B participant information sheet version [insert number] dated [dd/mm/yyyy].	Please initial
**2.** I have had time to think about the information I have been given about the CERM trial and ask questions, and I am satisfied with the answers I have been given.	Please initial
**3.** I understand that I do not have to take part and that I can withdraw from the CERM trial at any time, without giving a reason and without my medical care or legal rights being affected.	Please initial
**4.** I understand that the hospital research team will ask me for information about my past and current health that is relevant to me taking part in the CERM research trial. I give the hospital research team permission to collect and record this information.	Please initial
**5.** I understand that the hospital research team will collect information from my NHS hospital and GP records if it is relevant to me taking part in the CERM trial. I give the research team permission to access these records to collect and record this information.	Please initial
**6.** I understand that the research team and trial monitors from Warwick Clinical Trials Unit, the hospital trusts and regulatory authorities may have access to my medical and trial records to monitor this trial. I give permission for these people to access and record this information.	Please initial
**7.** I confirm that my partner and I have used condoms during this menstrual cycle. I will continue to use condoms whenever I am having sex throughout my entire menstrual cycle while I am waiting for the result of my biopsy and, if I take part in the randomised trial, while I am taking the capsules and preparing for my repeat biopsy and swabs.	Please initial
**8.** I have seen the result of my pregnancy test taken today and confirm that it showed that I am not pregnant.	Please initial
**9.** I agree to have an endometrial biopsy taken.	Please initial
**10.** I agree to my endometrial biopsy being held in the Tommy's National Reproductive Health Biobank until it is analysed.	Please initial
**11. Optional:** I agree that any tissue that is left over following my endometrial biopsy or after my endometrial biopsy is analysed can be stored in the Tommy's National Reproductive Health Biobank, with clinical data that does not identify me,- and used in future ethically approved research.	Please initial
**12.** I agree to have cervical, vaginal and endometrial swabs taken.	Please initial
**13.** I agree to my cervical, vaginal and endometrial swabs being stored in the Tommy's National Reproductive Health Biobank until they are analysed.	Please initial
**14. Optional:** I agree that any material that is left over after my cervical, vaginal and endometrial swabs are analysed can be stored in the Tommy's National Reproductive Health Biobank, with clinical data that does not identify me, and used in future ethically approved research.	Please initial

Please go to page 2.

Crystal Mark 23121 Clarity approved by Plain English Campaign

CERM Chronic Endometritis and Recurrent Miscarriage

Print on Trust headed paper

**15.** I agree to the hospital trial team storing my phone numbers so they can contact me with the results of my endometrial biopsy and to discuss the next steps.	Please initial
**16.** I agree to the hospital trial team storing my phone numbers and contacting me for follow-up information as explained in the participant information sheet (or sheets).	Please initial
**17.** I agree to the research team telling my GP that I am taking part in the CERM research trial.	Please initial
**18.** I agree to take part in the CERM research trial.	Please initial

We will only use the following consent if you later take part in the randomised controlled trial.

**19.** I understand that I should not take doxycycline while I am pregnant because it can affect the baby. I understand it is important to use condoms during my entire menstrual cycle when I am taking doxycycline or a placebo (whichever I am given).	Please initial
**20.** I agree to take doxycycline or a placebo for 14 days and only as instructed.	Please initial
**21.** I understand that if I give birth at another hospital, the hospital research team will contact the relevant hospital trust to collect medical details from my NHS hospital records (and my baby's) if this is relevant to taking part in the CERM research trial. I give the research team permission to access these records to collect and record this information.	Please initial
**22.** I understand that the hospital research team will collect information from my baby's NHS hospital and GP records if it is relevant to me taking part in the CERM trial. I give the research team permission to access these records to collect and record this information.	Please initial
**23.** I understand that the research team and trial monitors from Warwick Clinical Trials Unit, the hospital trusts and regulatory authorities may have access to my baby's medical and trial records to monitor this trial. I give permission for these people to access and record this information.	Please initial
**24.** I agree to have a pregnancy test taken when I come to hospital for a repeat endometrial biopsy. I understand I will be shown the result of the test to confirm I am not pregnant.	Please initial
**25.** I agree to have a repeat endometrial biopsy taken.	Please initial
**26.** I agree to my repeat endometrial biopsy being stored in the Tommy's National Reproductive Health Biobank until it is analysed.	Please initial
**27. Optional:** I agree that any tissue that is left over after my repeat endometrial biopsy is analysed can be stored in the Tommy's National Reproductive Health Biobank, **with clinical data that does not identify me,** and used in future ethically approved research.	Please initial
**28.** I agree to have repeat cervical, vaginal and endometrial swabs taken.	Please initial
**29.** I agree to my repeat cervical, vaginal and endometrial swabs being stored in the Tommy's National Reproductive Health Biobank until they are analysed.	Please initial
**30. Optional:** I agree that any material that is left over after my repeat cervical, vaginal and endometrial swabs are analysed can be stored in the Tommy's National Reproductive Health Biobank, **with clinical data that does not identify me,** and used in future ethically approved research.	Please initial
**31.** I agree to take part in the CERM randomised controlled trial.	Please initial

Please go to page 3.

Crystal Mark 23121 Clarity approved by Plain English Campaign

CERM
Chronic Endometritis and Recurrent Miscarriage

Print on Trust headed paper

**Your name** Please print	**Your signature** Please sign	**Date** dd/mm/yyyy
**Investigator's name** Please print	**Investigator's signature** Please sign	**Date** dd/mm/yyyy

Odendaal J, *et al. BMJ Open* 2023; 13:e081470. doi: 10.1136/bmjopen-2023-081470

Possible side effects

If you get any of the following side effects, stop taking the capsules. Go to an emergency department and take your CERM trial participant card with you.

- Chest pain
- Difficulty breathing
- Swollen eyelids, face or lips
- Very severe headaches
- Severe skin reaction
- Fever or swollen lymph nodes
- Diarrhoea with blood in it

If you get any of the following side effects and you are concerned, call the hospital research team on [insert number].

- Feeling or being sick
- Heartburn
- Indigestion (dyspepsia)
- Gastritis (being sick)
- Vaginal infection

Please note: Very few women will get these side effects.

About the CERM trial

This is a new research trial designed to see if taking antibiotics reduces miscarriage.

If a woman has two or more miscarriages in a row, this is known as recurrent miscarriage. Some women who have recurrent miscarriage have an inflammation of the lining of the womb (chronic endometritis) and it is thought that this might prevent the embryo from attaching to the wall of the womb (implantation).

This trial is designed to find out if a two-week course of antibiotics will treat the inflammation and reduce the likelihood of a miscarriage.

During the trial, you will be given capsules to take each day. These will either contain an antibiotic (doxycycline) or a placebo (a substance with no medicinal effect, used as a control in clinical trials).

How to contact us

Email: <<<Insert site contact email>>>
Phone: <<<insert site number>>>
Address: <<<Insert Site Address>>>
Website: www.warwick.ac.uk/cerm

CERM
Chronic Endometritis and Recurrent Miscarriage

Crystal Mark 23107
Clarity approved by Plain English Campaign

Taking my capsules on the chronic endometritis and recurrent miscarriage (CERM) trial

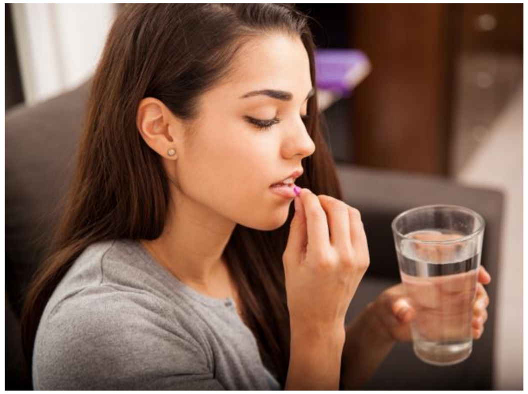

CERM Taking my capsules leaflet | Version 3.0 | 27/07/2020 | IRAS ID: 251756 | EudraCT 2019-000585-38_Final

How to take the capsules

- Start taking the capsules on the first day of your period.
- Take **one** capsule **twice** a day.
- Swallow the capsules with plenty of water and take them with milk or food.
- Space the doses 12 hours apart and do not take them immediately before bedtime or if you are lying down.
- Keep taking this medicine until you finish the course, unless you are told to stop.
- Protect your skin from sunlight by using sunscreen, wearing appropriate clothing and spending time in the shade – even on a bright but cloudy day. Do not use sunbeds.
- **Do not** take indigestion remedies, or medicines which contain iron or zinc, two hours before or after you take this medicine.
- It is important to use condoms and not have unprotected sex during this entire menstrual cycle.
- It is important that you keep your CERM trial participant card with you at all times during the two weeks you are taking the capsules.

What to do if something happens when you are taking the capsules	
What if I forget to take a capsule?	Please do not double your dose. Take a capsule as soon as you remember, within six hours, and then take the next dose as scheduled. (If you don't remember until after six hours have passed, miss that dose – see below.) It is important that you finish the course.
What if I miss a capsule?	Please do not double your dose. Continue to take the capsules as prescribed and take the missed capsule at end of the course. It is important that you finish the course.
What if I miss more than one capsule?	Please do not double your dose. Continue to take the capsules as prescribed, and take the missed capsules at end of the course. It is important that you finish the course.
What if I lose my capsules?	If you lose your capsules we will not be able to replace them.
What if I take too many capsules?	Get medical advice immediately. Show your CERM trial participant card to any staff treating you. Stop taking the capsules. Tell the research team after your medical consultation.
What if I am sick after taking a capsule?	If you are sick within two hours of taking the capsule, you can take another one. If you are sick more than two hours after taking a capsule, take the next dose at the usual time.
What if I develop a rash?	If you develop a rash, stop taking the capsules and get medical advice. Show your CERM trial participant card to any staff treating you. Tell the research team after your medical consultation.
What if I have an allergic reaction?	If you have an allergic reaction, stop taking the capsules and get medical advice. Show your CERM trial participant card to any health staff treating you. Tell the research team after your consultation.
What if I find out I am pregnant when I'm taking the capsules?	If you find out you are pregnant while you are still taking the capsules, please stop taking them immediately and contact the research team. If you find you are pregnant after you have finished taking the capsules, please tell the research team.
Can I take the capsules with other medication?	Not all medications are safe to take with the capsules. If a health professional advises you to take a **new** medication, either prescribed or over the counter, show them your CERM trial participant card.
What if I get an infection and need to take antibiotics?	If you get an infection that needs antibiotics, show the healthcare provider your CERM trial patient card and follow their advice regarding stopping the trial capsules.
Can I drink alcohol when I am taking the capsules?	Do not drink alcohol when you are taking the capsules.
What if anything else happens?	If anything happens that is not listed here, call us for advice on [insert number]
