# Supplementary material for: Preconceptual administration of doxycycline in women with recurrent miscarriage and chronic endometritis: protocol for the Chronic Endometritis and Recurrent Miscarriage (CERM) trial, a multicentre, double-blind, placebo-controlled, adaptive randomised trial with an embedded translational substudy
Source: BMJ Open. 2023 Dec 1;13(12):e081470. doi: 10.1136/bmjopen-2023-081470 (PMC10693855; doi:10.1136/bmjopen-2023-081470)
Supplement: Author's manuscript [file bmjopen-2023-081470.draft_revisions.pdf]

BMJ Open is committed to open peer review. As part of this commitment we make the peer review history of every article we publish publicly available.

When an article is published we post the peer reviewers' comments and the authors' responses online. We also post the versions of the paper that were used during peer review. These are the versions that the peer review comments apply to.

The versions of the paper that follow are the versions that were submitted during the peer review process. They are not the versions of record or the final published versions. They should not be cited or distributed as the published version of this manuscript.

BMJ Open is an open access journal and the full, final, typeset and author-corrected version of record of the manuscript is available on our site with no access controls, subscription charges or pay-per-view fees (http://bmjopen.bmj.com).

If you have any questions on BMJ Open's open peer review process please email info.bmjopen@bmj.com

BMJ Open

Chronic Endometritis and Recurrent Miscarriage (CERM): A study protocol for an integrated translational trial

Journal:	*BMJ Open*
Manuscript ID	bmjopen-2023-081470
Article Type:	Protocol
Date Submitted by the Author:	28-Oct-2023
Complete List of Authors:	Odendaal, Joshua; University of Warwick; University Hospitals Coventry and Warwickshire NHS Trust<br>Guck, Jonathan; University of Warwick<br>Fisher, Joanne; University of Warwick<br>Black, Naomi; University of Warwick; University Hospitals Coventry and Warwickshire NHS Trust,<br>Bouliotis, Georgios; University of Warwick<br>Underwood, Martin; University of Warwick, Warwick Medical School<br>Quenby, Siobhan; University of Warwick, Division of Reproductive Health, Warwick Medical School; University Hospitals Coventry and Warwickshire NHS Trust
Keywords:	Reproductive medicine < GYNAECOLOGY, Randomized Controlled Trial, Clinical Trial

SCHOLARONE™
Manuscripts

Chronic Endometritis and Recurrent Miscarriage (CERM): A study protocol for an integrated translational trial

Joshua Odendaal MRCOG PhD [1-3], Naomi Black MBBS MSc[1-2], Georgios Bouliotis PhD[4], Jonathan Guck MSc[4], Martin Underwood FRCGP MD[2, 4], Joanne Fisher PhD[4], Siobhan Quenby FRCOG MD [1,2].

[1]Division of Biomedical Sciences, Clinical Sciences Research Laboratories, Warwick Medical School, University of Warwick, Coventry CV2 2DX, United Kingdom.

[2]University Hospitals Coventry & Warwickshire, Coventry CV2 2DX, United Kingdom.

[3]Corresponding Author: Joshua Odendaal, Division of Biomedical Sciences, Clinical Sciences Research Laboratories, Warwick Medical School, University of Warwick, Coventry CV2 2DX, United Kingdom. Tel: 02476968702. Email: joshua.odendaal@warwick.ac.uk. ORCID ID: 0000-0001-7829-3729

[4]Warwick Clinical Trials Unit, Warwick Medical School, University of Warwick, Gibbett Hill, Coventry, CV4 7AL, United Kingdom.

Word Count: 3,728 words

For peer review only - http://bmjopen.bmj.com/site/about/guidelines.xhtml

Abstract

Introduction Recurrent miscarriage is a common condition with a substantial associated morbidity. A hypothesised cause of recurrent miscarriage is chronic endometritis (CE). The aetiology of CE remains uncertain. An association between CE and recurrent miscarriage has been shown. This study will aim to determine if the pre-conceptual administration of doxycycline, in women with recurrent miscarriages, and chronic endometritis, reduces first trimester miscarriages, increasing live births.

Methods and Analysis A prospective, multi-centre, double blind adaptive trial with an embedded translational sub-study. Women with a history of two or more consecutive first trimester losses with evidence of chronic endometritis on endometrial biopsy (defined as ≥5 CD138 positive cells per 10mm2) will be randomised to oral doxycycline or placebo for 14-days. A subset will be recruited to a mechanistic sub-study in which microbial swabs and pre-/post-intervention endometrial samples will be collected. Up to 3062 women are expected to be screened with up to 1500 women randomised in a 1:1 ratio. Women with a negative endometrial biopsy (defined as <5 CD138 positive cells per 10mm2) will also be followed up to test validity of the tool. The primary outcome is live births plus pregnancies ≥24 + 0 weeks gestation at the end of the trial, in the first or subsequent pregnancy. In addition, secondary clinical outcomes will be assessed. Exploratory outcomes will assess the effect of doxycycline treatment on the endometrial microbiota, the differentiation capacity of the endometrium and the senescent profile of the endometrium with CE.

Ethics and Dissemination Ethical approval has been obtained from the NHS Research Ethics Committee (REC) Northwest- Haydock (19/NW/0462). Once completed the study will be published in an open-access peer reviewed journal and reported in the NIHR journals library.

Trial Registration Number: ISRCTN23947730

For peer review only

Strengths and Limitations of this Study

1) This will be the largest randomised controlled trial assessing the role of antibiotics in improving livebirth rates in those with a history of recurrent miscarriage.
2) An integrated mechanistic design allows elucidation of both clinical and mechanistic outcomes.
3) Generation of a bank of data-linked samples for further CE research.
4) Recruitment may be difficult given the use of CE testing, relative low cost of doxycycline and treatment in the private sector and the limited equipoise within the specialist population.
5) Large number of secondary outcomes with reduced power for detection in this model but balanced by the increased benefits of an integrated design.

For peer review only

For peer review only - http://bmjopen.bmj.com/site/about/guidelines.xhtml

Introduction

Recurrent miscarriage is an important cause of reproductive associated morbidity. Defined as the loss of two or more pregnancies it has a population prevalence of up to 3% (1). It is research priority within reproductive health (2, 3). A Lancet review series highlighted the ongoing uncertainty around the aetiology of recurrent miscarriage (1). It is hypothesised that chronic endometritis is a cause of recurrent miscarriage (4). In chronic endometritis, there is asymptomatic inflammation of the endometrium mediated by infiltration of the endometrial stroma by plasma cells(5). CE remains of uncertain aetiology being differentially attributed to dysbiosis of the endometrial microbiota and the presence of pathological species within the endometrium (6-8). There is not consensus around the diagnosis of CE (9). Traditionally CE has been diagnosed on histology through the identification of infiltrating endometrial plasma cells (10). Recently this has been augmented using CD138 immunohistochemistry (11). Whilst this has resulted in improved sensitivity, the diagnostic thresholds remain variable and the impact on therapeutic outcome varies dependent on the CD138 threshold set (9). Liu et al., evaluated different diagnostic methodologies demonstrating a technique based on whole section density to have the lowest intra- and inter-observer variability (11). The study also identified a density threshold based on the 95% confidence interval with a differential prevalence of 22.2% in those with reproductive failure in comparison to 5% in fertile controls ($p<0.01$). McQueen et al., assessed the effect of antibiotic treatment in a cohort of women presenting with recurrent miscarriage or loss of a single fetus >10 weeks gestation (12). Following treatment with antibiotics, a similar cumulative livebirth rate (LBR) was seen in the CE group in comparison to controls (88% treated CE vs. 74% without CE; $p=0.215$). A significant increase in per pregnancy livebirth rate pre- and post- treatment for CE was also seen (7% pre-treatment LBR vs. 56% post treatment LBR; $p<0.001$). Similarly, a retrospective cohort study assessing for the presence of CE demonstrated a marked improvement in live birth rate in those with CE resolution following antibiotic therapy (78.4% LBR in resolved CE vs. 17.5% LBR persistent CE; $p<0.001$) (13). Doxycycline is the most commonly reported antibiotic therapy used in the treatment of CE. A single course has previously been reported to achieve histological resolution in 92% of cases (14). These findings, however, remain limited by sample size and methodological concerns including lack of randomisation and lack of untreated controls. Despite this however, commercial testing is now available within the private sector (15). Given the combined limitations in pathophysiological and clinical understanding of the condition a novel translation approach is required. In view of this the Chronic Endometritis and Recurrent Miscarriage (CERM) trial was developed as a novel translation approach to better determine the role of CE in recurrent miscarriage and the potential for therapeutic intervention.

For peer review only - http://bmjopen.bmj.com/site/about/guidelines.xhtml

Study Objectives

1. To determine if doxycycline administered prior to conception improves pregnancy outcome in women with recurrent miscarriage associated with chronic endometritis,

2. To explore the mechanisms by which it could prevent miscarriage, and

3. To determine if CD138 values predict miscarriage

Primary Outcome

The primary outcome is total live births plus ongoing pregnancies ≥24 + 0 weeks gestation at the end of the trial, in the first or subsequent pregnancy. This measure was chosen as the most meaningful outcome to patients.

Secondary Outcomes

Given aetiological uncertainty a cluster of both clinical and pathophysiological secondary outcomes have been selected. These include: Ongoing pregnancy at 12 weeks gestation, time to first conception, anticipated time to first livebirth, the proportion with livebirth ≥24 weeks gestation in the first pregnancy, pregnancy complications, type of miscarriage, ongoing pregnancy rates, livebirth rates in those excluded from randomisation as CE negative, the predictive value of CD138+ cell density on pregnancy outcome, the impact of intervention on endometrial $CD138^{+}$ cell density, and termination for social reasons.

Exploratory Outcomes

Given the translational nature of the trial, a cluster of exploratory outcomes will also be assessed. These include an assessment of the effect of doxycycline treatment on the endometrial microbiota, the differentiation capacity of the endometrium and the senescent profile of the endometrium with CE.

Methods and Analysis

The protocol (V8.3 22 June 2023) was designed in accordance with Standard Protocol Items: Recommendations for Interventional Trials guidelines (16). The trial has been prospectively registered on the ISRCTN registry (ISRCTN23947730).

Study Design

The CERM trial is a prospective, multi-centre, double blind adaptive trial. It is a multi-phased trial with a pre-eligibility phase, screening phase, randomisation phase and two separate follow-up

For peer review only - http://bmjopen.bmj.com/site/about/guidelines.xhtml

pathways dependent on-screen status. These are summarised in figure 1. This is a trial of adaptive design with three prespecified interim stops to assess for futility at 100, 300 and 900 participants. Based on the unblinding results, the trial may stop after Data Monitoring Committee (DMC) recommendation, if there is low or negligible treatment efficacy. In addition, the use of on-going pregnancy as a surrogate marker will also increase the trial flexibility whilst maintaining evidential rigour. The trial also has a parallel translational sub-study assessing the endometrial and microbial effects of a diagnosis of CE and the impact of treatment on this. Utilising the clinical trial to develop a sample cohort will allow assessment of both the exploratory outcomes and allow further basic science assessment of the impact of CE with the aim of identifying the pathophysiological sequence of the condition alongside the potential mechanism of treatment effect.

The clinical trial is conducted and managed by the Warwick Clinical Trials Unit (WCTU). The trial has been sponsored by University Hospitals Coventry and Warwickshire (UHCW). The trial is funded by the Efficacy and Mechanism Evaluation (EME) Programme, an MRC and NIHR partnership, project number 17/60/22. Standard Protocol Items: Recommendations for Interventional Trials reporting guidelines were used for this protocol (16).

Trial Setting

The initial aim of the trial was to recruit up to 3062 women to the screening phase with an expected up to 1500 women randomised from 10 tertiary recurrent miscarriage centres. Given the decentralisation of recurrent miscarriage care however, a decision was taken to widen participation allowing trial set-up across all NHS trusts with a working recurrent miscarriage service.

Translational sub-study

Women recruited at the sponsor site will be entered into the optional translational sub-study. These women will undertake a dual consent process for both the clinical trial and for samples and associated clinical data to be banked within a reproductive health biobank allowing both flexibility and regulatory oversight of additional scientific exploratory work that emerges from endometrial assessment.

Women entered into the translational sub-study will undergo sampling of the microbiota in addition to endometrial histological sampling. This will be achieved through the concomitant collection of high vaginal swabs, endocervical swabs and uterine swabs alongside an outpatient endometrial biopsy. Consent to the sub-study will be undertaken by the research team at the sponsor site.

Environmental and negative controls will be collected across each collection day to adjust for potential contamination effects. Women who are screen positive will be offered repeat endometrial

For peer review only - http://bmjopen.bmj.com/site/about/guidelines.xhtml

sampling in the intervention cycle to ascertain the endometrial effects of treatment. Overall, up to 200 paired samples will be obtained. A random sample of high vaginal and cervical swabs will be analysed to ascertain risk of contamination, the remainder will be stored. All endometrial swabs will be analysed.

Participants and Recruitment

Eligibility for inclusion is dependent on the phase of the trial. The key entry point and assessment of eligibility is within the screening phase. These inclusion and exclusion criteria are as below.

Inclusion Criteria

Women aged ≥18 to <42 who have experienced two or more consecutive first trimester miscarriages, with first trimester miscarriage defined as an intrauterine pregnancy loss at ≤14 weeks gestation. Women who have suffered an ectopic pregnancy, a molar pregnancy or 2nd trimester miscarriage between or after their first trimester miscarriages will still be eligible given the differing pathology of loss.

Exclusion criteria

Women will be excluded if they experienced a livebirth, stillbirth or termination of pregnancy between or after their first trimester miscarriage. In addition, women with a known treatable cause of RM will be excluded including antiphospholipid syndrome or uncontrolled thyroid disease. Women with a menstrual cycle ≤21 or ≥42 days will be excluded due to difficulties in timing the sampling and/or treatment. Those with systemic inflammatory conditions including Systemic Lupus Erythematous and Myasthenia Gravis will be excluded. Finally, those on long term antibiotics, having undertaken antibiotic therapy in the screening menstrual cycle or with a contraindication to doxycycline will be excluded.

Trial Procedure

Pre-eligibility phase

Women eligible as above will be advised to avoid pregnancy and provided with ovulation tests. They will be verbally consented to the study by a clinician, trained research nurse or midwife. In a subsequent menstrual cycle, during which any intercourse involved use of barrier contraception, they will undertake ovulation testing and contact the research unit at the point of ovulation to book the screening biopsy.

Screening for Chronic Endometritis

For peer review only - http://bmjopen.bmj.com/site/about/guidelines.xhtml

A medical doctor will confirm eligibility and obtain consent. Women will undergo endometrial screening on day 10 ±4 following ovulation. Where ovulation testing is inconclusive, screening will be performed on day 23 ±2. An outpatient endometrial biopsy is undertaken following a negative pregnancy test. Additional samples are undertaken as above in those included within the translational sub-study. Samples will be collected by those who do so as part of their job role. Where insufficient samples are obtained the participant has the option for a repeat attempt at collection in the same or next menstrual cycle. Alternatively, they can be referred to the lead site for sample collection. A maximum of two biopsies are be attempted.

Samples will be processed in a central laboratory. A standard protocol for analysis will be followed. A minimum section size of $2mm^2$ should be present. CD138 immunohistochemistry will be undertaken, and a diagnosis of CE will be made in the presence of ≥5 CD138 positive cells per $10mm^2$ of endometrial section based on the existing literature (11). Samples will be reported as positive, or negative, but additional stratification information will be utilised for analysis. Samples will be stratified into 4 categories to further aid prognostic analysis: negative (<5 CD138+/$10mm^2$), mild (5-20 CD138+/$10mm^2$), moderate (21-200 CD138+/$10mm^2$) and severe (>200 CD138+/$10mm^2$). In addition, the pattern of CD138 staining will be recorded and the presence of morphological appearance of plasma cells. Borderline samples will be assessed by two trained observers.

Additional Samples

Microbial samples collected as described previously are immediately placed on ice and transferred to a -80°C freezer. Samples are transferred to a specialist laboratory where ribonucleic acid (RNA) extraction will be performed and 16S sequencing conducted. Sequenced data will be passed down a pre-designed data pipeline to allow profiling of the microbiota. These will be analysed in conjunction with trial data and CE status. Additionally, remnant tissue at the lead site will be split between 2mls RNA later and stored at -80°C for RNA related exploratory studies and 4mls 10% dextran coated charcoal treated foetal bovine serum (DCC-FBS) supplemented with DMEM-F12 for culture studies.

Randomisation

Following a screen positive result for CE, women are eligible for randomisation provided they remain eligible based on the previous entry criteria, have not subsequently received a course of antibiotics, or had a delay of longer than three months since the biopsy was undertaken. Consent is further confirmed by a medical doctor, and randomisation undertaken by a member of the site research team using an online web application hosted by the Warwick Clinical Trials Unit. Block randomisation

For peer review only - http://bmjopen.bmj.com/site/about/guidelines.xhtml

is performed in a 1:1 ratio, with Minimisation by age (<35 vs ≥35 years), number of previous miscarriages (≤3 vs >3) and site.

Intervention

Pre-conceptual doxycycline or placebo over-encapsulated for the same appearance will be administered orally at 100mg twice a day for 14-days. Women are advised to commence the intervention on day 1 of the subsequent menstrual cycle. Those entered into the sub-study will undergo ovulation testing within this cycle with repeat biopsy timed as per the screening phase biopsy. Women will be asked to self-record compliance with the intervention.

Safety Mechanisms

Doxycycline is contra-indicated in pregnancy as its use has been reported as teratogenic (17, 18). Nevertheless, a review by Cross et al reported an absence of teratogenic effects and advised the use of doxycycline where clinical circumstances indicate (19). Safety mechanisms have, however, been built into the trial to mitigate the risk of inadvertent use in pregnancy. These include the use of a pregnancy test prior to commencement of doxycycline, commencement on the first day of the menstrual period hence ensuring course completion prior to ovulation and finally women are advised to use barrier contraception throughout the remainder of the cycle. Any pregnancies occurring within 4-weeks of the intervention will be reported and thorough assessment performed to assess for evidence of congenital abnormalities. Emergency unblinding is possible via a 24-hour code breaking telephone line. This will be performed for clinical indications.

Follow-Up

Two separate follow-up pathways exist dependent on CE screen status. Women who screen negative will be followed-up to assess subsequent pregnancy outcome at 3-, 6- and 12-months post-biopsy or to the first complete pregnancy outcome, whichever comes first. Women who screen positive will be followed up at 3, 6, 12 and 24 months from the point of intervention to the first pregnancy outcome after 24-weeks gestation, 24 months of follow up or the end of the trial, whichever comes first.

End of trial

Given the adaptive nature of the trial the end can be determined by one of several factors. Firstly, following recruitment and completion of 14-months of subsequent follow-up of 1500 women. Secondly, where interim analysis demonstrates either efficacy or futility. Thirdly where mandated by the ethics committee. Fourthly where recruitment falls substantially below target for a period

For peer review only - http://bmjopen.bmj.com/site/about/guidelines.xhtml

including but not limited to ≤68 women randomised in the first 6-months. Finally, where trial funding ceases.

Data Collection

Pseudo-anonymised data attributed to trial ID will be collected and inputted at sites into a dedicated secure trial database. Warwick Clinical Trials Unit and UHCW (sponsor) act as joint data controllers for this trial.

Data collected at baseline includes date of birth, ethnicity, body mass index, smoking history, medical history, obstetric/pregnancy history, concomitant medication, pregnancy status, a contraception review, and a review of the participant's menstrual cycle. Further information on the timing of the biopsy and any use of antibiotics in the menstrual cycle will be collected at the screening visit. At the randomisation visit, information on concomitant medication, pregnancy status, use of contraception and adverse events will be collected. After the anticipated date of treatment completion, data will be collected on treatment compliance, ongoing contraceptive use, and any adverse events. Follow up will occur at 3, 6, 12 and 24 months after randomisation to ascertain pregnancy status. For women who are pregnant, further follow-up will be timed to key pregnancy stages. Pregnancy dating will be confirmed at the booking ultrasound through crown-rump length, viability assessment, concomitant medication review, and any developed pregnancy complications will be noted. Similar information will be captured at ongoing pregnancy reviews. Post-pregnancy, details of the pregnancy outcome, placental histology if available, cytogenetics of products of conception if applicable, pregnancy complications, congenital abnormalities, immediate postpartum maternal and infant infections and maternal infections up to 8-weeks post-delivery, will be collected. All trial participants are free to withdraw from the trial at any time point. Unless a participant explicitly withdraws consent, they and their infant will be followed up where possible. On withdrawal, no further data will be collected and, if requested, all participant data will be removed.

Sample size

The total number of women entered for screening will be determined by the CE positivity ratio on screening. A 50% positivity rate has been assumed with a total screen population of 3062 accounting for participant drop out of 2%. This will give a maximum sample size of 1500 randomised with 750 in each arm. The study is therefore powered to detect an 8% between group difference this has been pragmatically selected due to limited previous high-quality work in the area. There are three planned interim analyses after 100, 300 and 900 women have reached 28 weeks post-randomisation. These analyses will assess for futility and be reviewed by the Data Monitoring

For peer review only

For peer review only - http://bmjopen.bmj.com/site/about/guidelines.xhtml

Committee (DMC). Prespecified stopping rules as documented in the statistical analysis plan will be applied.

Data Analysis

Data will be analysed by intention to treat at randomisation. The treatment difference for the primary outcome will be assessed by deriving the posterior distribution of a Bayesian logistic regression model with a weakly informative prior distribution for all parameters. Although unlikely, in the event of missing data for primary outcomes of >10% imputation techniques will be used. The result for the primary outcome will be reported as the median value of the simulated odds ratios with the corresponding credible intervals. Secondary outcomes as previously outlined will be similarly assessed. The time to event (first pregnancy) analysis will be assessed using semiparametric proportional hazard regression. The following pre-defined subgroup analyses of the primary outcomes will also be assessed by age (<35 vs ≥35), number of previous miscarriages (≤3 vs >3), severity of CD138 expression and pattern of CD138 expression.

Ethics and Dissemination

The trial will be conducted in full conformance with the principles of the Declaration of Helsinki, ICH Good Clinical Practice (GCP) guidelines and the UK Statutory Instrument Number 1031 that implements the Medicine for Human Use (Clinical Trials) Directive 2004 and subsequent amendments. Ethical approval for the trial has been obtained from the NHS Research Ethics Committee (REC) Northwest- Haydock (REC reference: 19/NW/0462, REC approval date: 20/08/2019). The key ethical consideration of the trial revolves around the paradox of asking women who are eager to achieve successful pregnancy to avoid pregnancy for three months whilst undergoing trial procedures. To mitigate this, trial procedures will be kept to as short a period as possible. It is hoped the potential for gain minimises this paradox.

Adverse Event Management

Adverse events (AE) will be collected to 30 days post-trial intervention, this may be either screening biopsy, treatment completion or secondary biopsy whichever comes later dependent on the participants trial arm. Adverse events captured elsewhere in the trial data do not require reporting, including those related to pregnancy outcome. In addition, common pregnancy related serious adverse events as seen in table 1 are exempt from SAE reporting but should be reported as adverse events. A causality assessment will be performed by a doctor for all SAEs and Aes. These will be assessed by the trial management group and the data monitoring committee. SAEs and Aes thought to be related or unexpected will be reported to the Medicines and Healthcare Products Regulatory

For peer review only - http://bmjopen.bmj.com/site/about/guidelines.xhtml

Agency) and Research Ethics Committee, the sponsor and the chairs of the Trial Steering Committee and Data Monitoring Committee.

**Table 1: Adverse events exempt from SAE reporting but recorded as Aes**
**Admission to hospital for nausea and vomiting**
**Admission to hospital for headaches**
**Admission to hospital for raised blood pressure or pregnancy induced hypertension**
**Admission to hospital for vaginal bleeding**

Monitoring

For peer review only

All clinicians involved in consent should have up to date Good Clinical Practice training. Samples may be collected by those who fulfil this role in a clinical capacity. All involved will undergo a relevant programme of training. Site visits will be conducted where concerns arise. Data will be monitored for completeness and quality, where queries arise these will be escalated to the site involved.

A Trial Management Group (TMG) comprising co-investigators, allied experts, a patient public involvement advisor and the trial management staff will meet monthly to bimonthly to review the day-to-day running of the trial.

A Trial Steering Committee (TSC) has been established comprising a group of independent experts covering all trial areas and lay members. The TSC will be responsible for protocol approval and review of any protocol changes, advising on aspects of trial conduct, monitoring trial progress and consideration of any recommendations from the Data Monitoring Committee (DMC). They will meet not less than once a year. Any protocol modifications as agreed by the TSC will be submitted to relevant parties.

A Data Monitoring Committee (DMC) has been established comprising independent experts. They will ensure monitoring of outcomes and safety aspects during the trial.

An authorised representative of the Sponsor has approved the final version of the protocol with respect to the trial design, conduct, data analysis and interpretation and plans for publication and dissemination of results.

Patient and public involvement

The trial has been designed with the involvement of the Lily-Mae Foundation a charity supporting women who have lost a baby through miscarriage, stillbirth, neonatal death or medical termination.

For peer review only - http://bmjopen.bmj.com/site/about/guidelines.xhtml

Amy Jackson co-founder of the charity sits on the trial management group. She has additionally led on the development of patient facing materials.

Dissemination

The trial will be reported in accordance with the Consolidated Standards of Reporting Trials (CONSORT) guidelines (20). The trial will be published in an open-access peer reviewed journal with authorship agreed as per ICMJE requirements (21). The full protocol and trial findings will also be reported in the NIHR journals library. In addition, dissemination at national and international specialist conferences will be undertaken. Exploratory outcomes will be disseminated separately in open-access peer reviewed journals to accommodate their more specialist nature.

Trial Progress to Date

This trial opened for recruitment on 19 December 2019 and closed to new registrations on 30 September 2022. The trial is currently in the follow-up period this is due to end on 31st October 2023 and is expected to end on 30 April 2024.

Integrated Trial Design

This trial utilises a integrated adaptive approach. This allows facilitation of both assessing the core clinical outcomes but also the development of a concurrent sample and dataset for mechanistic evaluation should an effect be seen. It also allows better understanding of the aetiology of the condition including through the follow-up of those with a negative result. This integrated mechanistic clinical approach forms a model for a more economical approach to trial design in poorly understood conditions. Through more efficient use of resources, it allows the development of a holistic approach to disorder determination. A key hallmark of this design is the flexibility provided by the adaptive design and integration of sample banking. This ensures that a rigorous methodological approach is taken whilst still facilitating exploratory research. In fields such as this, which face the difficult challenges of a limited evidence base and disorder heterogeneity such approaches are likely to prove increasingly necessary.

For peer review only

For peer review only - http://bmjopen.bmj.com/site/about/guidelines.xhtml

References

1. Quenby S, Gallos ID, Dhillon-Smith RK, Podesek M, Stephenson MD, Fisher J, et al. Miscarriage matters: the epidemiological, physical, psychological, and economic costs of early pregnancy loss. The Lancet. 2021 2021-05-01;397(10285):1658-67.

2. Prior M, Bagness C, Brewin J, Coomarasamy A, Easthope L, Hepworth-Jones B, et al. Priorities for research in miscarriage: a priority setting partnership between people affected by miscarriage and professionals following the James Lind Alliance methodology. BMJ Open. 2017 Aug 23;7(8):e016571.

3. Department of Health & Social Care. Policy Paper: Women's Health Strategy for England. In: Department of Health & Social Care, editor. 2022.

4. Odendaal J, Quenby S, Sammaritano L, Macklon N, Branch DW, Rosenwaks Z. Immunologic and rheumatologic causes and treatment of recurrent pregnancy loss: what is the evidence? Fertil Steril. 2019 Dec;112(6):1002-12.

5. McQueen DB, Perfetto CO, Hazard FK, Lathi RB. Pregnancy outcomes in women with chronic endometritis and recurrent pregnancy loss. Fertil Steril. 2015 Oct;104(4):927-31.

6. Moreno I, Codoner FM, Vilella F, Valbuena D, Martinez-Blanch JF, Jimenez-Almazan J, et al. Evidence that the endometrial microbiota has an effect on implantation success or failure. Am J Obstet Gynecol. 2016 Dec;215(6):684-703.

7. Moreno I, Cicinelli E, Garcia-Grau I, Gonzalez-Monfort M, Bau D, Vilella F, et al. The diagnosis of chronic endometritis in infertile asymptomatic women: a comparative study of histology, microbial cultures, hysteroscopy, and molecular microbiology. American Journal of Obstetrics and Gynecology. 2018 2018/06/01/;218(6):602.e1-.e16.

8. Cicinelli E, De Ziegler D, Nicoletti R, Colafiglio G, Saliani N, Resta L, et al. Chronic endometritis: correlation among hysteroscopic, histologic, and bacteriologic findings in a prospective trial with 2190 consecutive office hysteroscopies. Fertil Steril. 2008 Mar;89(3):677-84.

9. Huang W, Liu B, He Y, Xie Y, Liang T, Bi Y, et al. Variation of diagnostic criteria in women with chronic endometritis and its effect on reproductive outcomes: A systematic review and meta-analysis. J Reprod Immunol. 2020 Aug;140:103146.

10. Puente E, Alonso L, Laganà AS, Ghezzi F, Casarin J, Carugno J. Chronic Endometritis: Old Problem, Novel Insights and Future Challenges. Int J Fertil Steril. 2020 Jan;13(4):250-6.

11. Liu Y, Chen X, Huang J, Wang CC, Yu MY, Laird S, et al. Comparison of the prevalence of chronic endometritis as determined by means of different diagnostic methods in women with and without reproductive failure. Fertil Steril. 2018 May;109(5):832-9.

For peer review only - http://bmjopen.bmj.com/site/about/guidelines.xhtml

12. McQueen DB, Bernardi LA, Stephenson MD. Chronic endometritis in women with recurrent early pregnancy loss and/or fetal demise. Fertil Steril. 2014 Apr;101(4):1026-30.

13. Cicinelli E, Matteo M, Tinelli R, Pinto V, Marinaccio M, Indraccolo U, et al. Chronic endometritis due to common bacteria is prevalent in women with recurrent miscarriage as confirmed by improved pregnancy outcome after antibiotic treatment. Reprod Sci. 2014 May;21(5):640-7.

14. Kitaya K, Matsubayashi H, Takaya Y, Nishiyama R, Yamaguchi K, Takeuchi T, et al. Live birth rate following oral antibiotic treatment for chronic endometritis in infertile women with repeated implantation failure. Am J Reprod Immunol. 2017 Nov;78(5).

15. Clain E, Devine K. Endometrial receptivity, to test or not to test: the evidence on contemporary assays. F&S Reviews. 2023 2023/01/01/;4(1):50-65.

16. Chan AW, Tetzlaff JM, Altman DG, Laupacis A, Gøtzsche PC, Krleža-Jerić K, et al. SPIRIT 2013 statement: defining standard protocol items for clinical trials. Ann Intern Med. 2013 Feb 5;158(3):200-7.

17. Muanda FT, Sheehy O, Bérard A. Use of antibiotics during pregnancy and the risk of major congenital malformations: a population based cohort study. British Journal of Clinical Pharmacology. 2017 2017/11/01;83(11):2557-71.

18. Abd-Allah ER, Abd El-Rahman HA. Influence of doxycycline administration on rat embryonic development during organogenesis. Journal of Biochemical and Molecular Toxicology. 2021 2021/01/01;35(1):e22613.

19. Cross R, Ling C, Day NP, McGready R, Paris DH. Revisiting doxycycline in pregnancy and early childhood--time to rebuild its reputation? Expert Opin Drug Saf. 2016;15(3):367-82.

20. Schulz KF, Altman DG, Moher D, the CG. CONSORT 2010 Statement: updated guidelines for reporting parallel group randomised trials. BMC Medicine. 2010 2010/03/24;8(1):18.

21. Editors ICoMJ. Defining the Role of Authors and Contributors 2023 [cited 2023 24 August]. Available from: https://www.icmje.org/recommendations/browse/roles-and-responsibilities/defining-the-role-of-authors-and-contributors.html.

For peer review only - http://bmjopen.bmj.com/site/about/guidelines.xhtml

Authors' contributions:

SQ, JF, & MU were applicants on the original grant application, SQ is the chief investigator. JO wrote first draft of this paper, NB, GB, and JG commented on successive drafts of the paper. All authors read and approved the manuscript.

Funding: This project is funded by the Efficacy and Mechanism Evaluation (EME) Programme, an MRC and NIHR partnership (Funder reference: 17/60/22). The views expressed in this publication are those of the authors and do not necessarily reflect those of the MRC, NIHR or Department of Health and Social Care.

Competing interests' statement:

None declared

Additional study details:

Trial sponsor: University Hospitals Coventry and Warwickshire NHS Trust (Sponsor reference: SQ411218). Sponsor contact: Ceri Jones, ceri.jones@uhcw.nhs.uk

Roles and responsibilities:

Trial Steering committee (TSC): Role – Approval protocol, advise on aspects of trial conduct, monitor trial progress, consider recommendations from DMC and if necessary agreeing changes to the protocol. TSC members - Andrew Horne (Chair), Jim Thornton (Clinical Expert), Sally Kerry (Medical Statistician), Rachel Roberts (Public Member), Ceri Jones (Sponsor observer), George Bouliotis (Medical Statistician), Siobhan Quenby (Chief Investigator).

Data Monitoring Committee (DMC): Role – Ensure monitoring of outcomes and safety aspects during the trial. Members - Alex McConnachie (Chair/Statistician), Sharon Cameron (Clinical expert member), Mary Stephenson (Clinical expert member), George Bouliotis (Trial statistician). The DMC is an independent committee from the trial sponsor.

Patient and public involvement:

Patients and/or the public were involved in the design, or conduct, or reporting, or dissemination plans for this research. These involvements are detailed in the methods section.

Supplementary Information:

1) **Participant Information Leaflet**
2) **Participant Consent Form**

For peer review only - http://bmjopen.bmj.com/site/about/guidelines.xhtml

For peer review only

3) Participant instruction leaflet

For peer review only

For peer review only - http://bmjopen.bmj.com/site/about/guidelines.xhtml

Figure Legends

Figure 1: Study participant flowsheet.

For peer review only

For peer review only - http://bmjopen.bmj.com/site/about/guidelines.xhtml

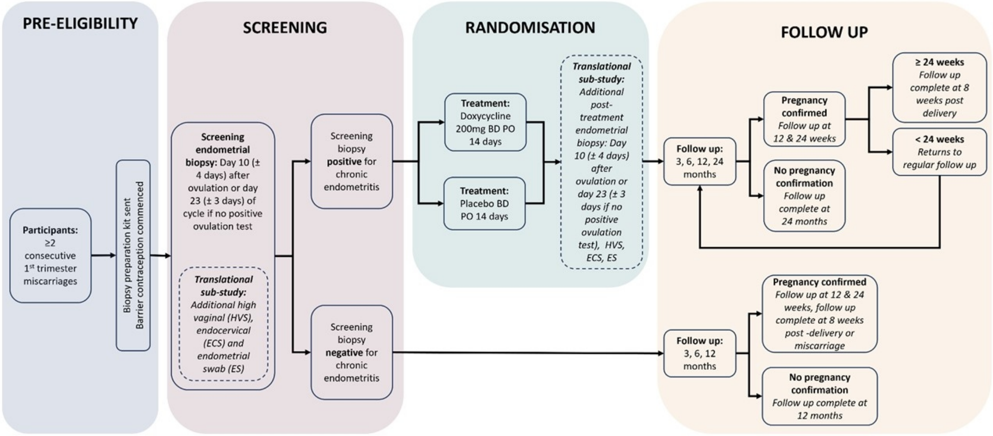

Figure 1: Study participant flowsheet.

338x190mm (96 x 96 DPI)

CERM Chronic Endometritis and Recurrent Miscarriage

[Print on Trust Headed Paper]

Crystal Mark 23135 Clarity approved by Plain English Campaign

Participant information sheet (CERM A)

Information for women taking part in the chronic endometritis and recurrent miscarriage (CERM) trial

Trial title

Chronic endometritis and recurrent miscarriage – the CERM trial

This information sheet is available in **large print** from the university trial team (email: cerm@warwick.ac.uk).

Introduction

You are invited to take part in a research trial. Please read this information sheet carefully before deciding whether to take part. It explains why this research trial is being done and what it means for you if you decide to take part. You will have the opportunity to talk to a member of the hospital research team about this trial and they will be happy to answer any questions you have. You can also discuss the trial with your GP or the obstetrician or gynaecologist caring for you.

This leaflet is divided into two parts. **Part 1** tells you the purpose of the trial and what will happen to you if you take part. **Part 2** gives you more detailed information about how the trial will be carried out.

Part 1

What is the purpose of the research trial?

The aim of this research trial is to find out if antibiotics can reduce miscarriage. In some women the lining of the womb (the endometrium) is inflamed. Researchers have found a link between this and miscarriage. A healthy endometrium is important for the embryo to be able to attach to the womb. It is thought that endometritis disrupts this process, and can lead to a miscarriage. Treating endometritis with antibiotics may reduce the inflammation and the likelihood of a miscarriage. This has not been tested. This research trial will test this theory by comparing a 14-day course of an antibiotic (doxycycline) against a placebo (a 'dummy treatment' which will look exactly the same as the antibiotic but contains no active ingredients) to find out if taking antibiotics reduces miscarriages. The trial will be 'double blind'. This means that the women and the trial researchers will not know who is taking the antibiotic capsules and who is taking the placebo. It will also be a randomised controlled trial. This means, if you decide to take part, which capsules you receive (the antibiotic or the placebo) will be decided by chance. The trial will take place in NHS hospitals in the United Kingdom and will involve over 3,000 women who have recurrent miscarriage.

Inflammation of the lining of the womb is a condition called **endometritis.**

Recurrent miscarriage means two or more miscarriages in a row.

For peer review only - http://bmjopen.bmj.com/site/about/guidelines.xhtml

CERM Chronic Endometritis and Recurrent Miscarriage

[Print on Trust Headed Paper]

Not everyone who has recurrent miscarriage has endometritis, so before you can take part in the randomised controlled trial the hospital trial team will need to take an endometrial biopsy (a small sample of tissue from your endometrium) and examine it under a microscope to find out if you do. We (the university research team) estimate that half the women tested will have endometritis and so be able to take part in the randomised controlled trial.

Researchers also suspect that inflammation of the endometrium may be caused by an imbalance of the microbiome that lives in the reproductive tract (the vagina, cervix, womb, fallopian tubes and ovaries). Another part of the trial is to look at the endometrium and microbiome to see how antibiotics affect these. A diagram of this research trial is shown in figure 1.

The **microbiome** is the name given to all the microbes that live in and on our bodies. It is mostly made up of bacteria, and everyone's microbiome is unique.

For peer review only

For peer review only - http://bmjopen.bmj.com/site/about/guidelines.xhtml

CERM
Chronic Endometritis and Recurrent Miscarriage

[Print on Trust Headed Paper]

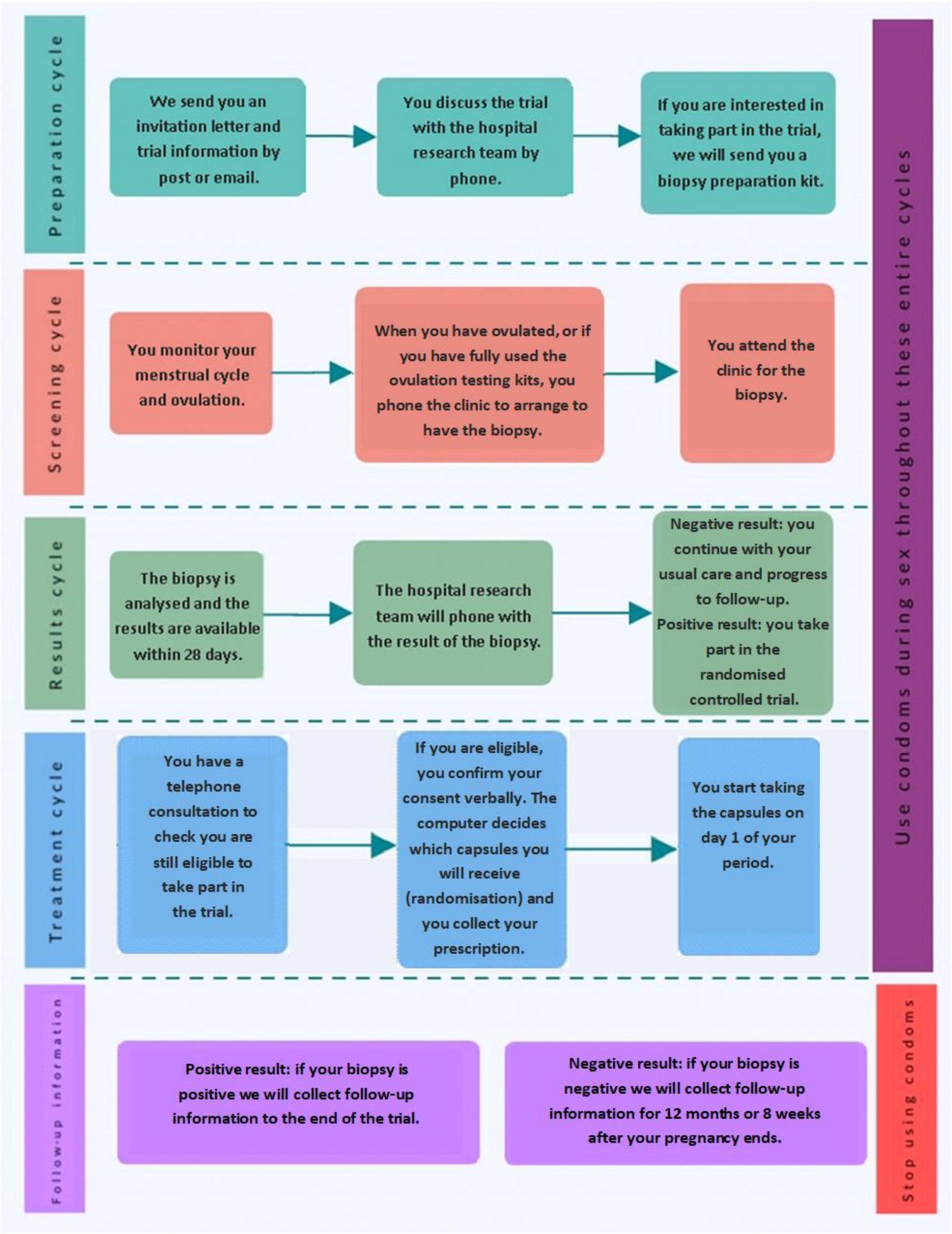

Figure 1 – an overview of the CERM trial

For peer review only - http://bmjopen.bmj.com/site/about/guidelines.xhtml

[Print on Trust Headed Paper]

Screening trial

Why have I been invited?

You have been invited to take part in this research trial because you have had two or more miscarriages (recurrent miscarriage).

Do I have to take part?

Taking part in the research trial is entirely voluntary. It is up to you whether or not you take part. You do not have to take part, and there will be no difference in the care you receive if you choose not to take part. If you want to take part, you will be asked to sign a consent form to confirm you have agreed to take part. You will keep a signed copy of the form. Even after signing the consent form, you can withdraw from the trial at any time if you change your mind, without having to give a reason. This will not affect the care you receive.

This information sheet will now explain what will happen if you decide to take part in this trial.

What will happen next?

A member of the hospital research team will talk to you over the phone (or speak to you face-to-face if you prefer this and if it is possible) to explain what the trial is about and what taking part would mean for you. You can ask the hospital research team any questions you have.

What will happen if I agree to take part?

1. **We will check to see if you are eligible –** You must be aged 18 to 41 years old. The hospital research team will ask about your obstetric and medical history to see if you are eligible to take part in the research trial. If you have not had any investigations to find out the cause of your recurrent miscarriage, these will be done when you attend the clinic. If a cause for your miscarriages is found, you will not be eligible for the trial. An important part of the eligibility check is to find out if you are willing to use condoms whenever you have sex throughout the entire menstrual cycles when you are:
    - preparing for your biopsy;
    - waiting for the result of your biopsy; and
    - taking the capsules.

    Doxycycline, the antibiotic used in the study, can be passed on in breast milk. The effects this could have on babies are not known. For this reason, and to make sure the study is absolutely safe, you will not be able to join the trial if you are breastfeeding.

2. **We will ask for permission to send you a 'biopsy preparation kit' –** If you are eligible to have a biopsy taken, the hospital research team will ask for your verbal consent to post a biopsy preparation kit to you. If you agree, this will be recorded in your hospital case notes and you will be given a trial screening number. The kit contains instructions, a period tracker, condoms and an ovulation testing kit. If you are prescribed a course of antibiotics between giving verbal consent and receiving the biopsy preparation kit, or

For peer review only - http://bmjopen.bmj.com/site/about/guidelines.xhtml

CERM Chronic Endometritis and Recurrent Miscarriage

[Print on Trust Headed Paper]

while you are using the biopsy preparation kit, please contact a member of the hospital research team (see the section: How can I contact the hospital trial team?). The biopsy may need to be delayed and the hospital team may need to send you another biopsy preparation kit once you have finished your course of antibiotics.

3. **Preparing for the biopsy –** It is very important that you **do not** try to conceive and that you use condoms whenever you have sex throughout this entire menstrual cycle. Condoms are provided in the kit and you can get more by contacting the hospital research team or family-planning services.

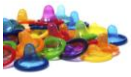

Starting on the first day of your next menstrual cycle after receiving the kit, you should use the period tracker to record:

- the days of your period; and
- the results of the ovulation testing.

The biopsy needs to be taken a few days after you have ovulated. Ovulation usually happens around day 12 to 16, depending on the length of your cycle. We provide a period tracker and ovulation testing kit to help you work out the best time to have the biopsy taken. When the ovulation test shows you have ovulated, or if you have used all of the kit and it does not show you have ovulated, you will need to contact the hospital research team to arrange an appointment to come in for your biopsy.

4. **Having the biopsy –** Please bring your period tracker with you when you come to the clinic for your biopsy. In the clinic a doctor will explain what will happen and check you are eligible to take part in the trial. You will have the opportunity to ask them any questions you might have. You are welcome to bring your partner, a family member, or a friend to this appointment, if the local rules on visitors to the clinic allow this. If you are eligible and would like to take part, you will be asked to sign a consent form. You will also be asked to take a pregnancy test before the biopsy. A healthcare professional will ask you for a sample of urine and they will test it. If the test shows you are pregnant, the biopsy will be cancelled because it could put the pregnancy at risk, and you will be referred to your GP who will arrange for your care. If the pregnancy test shows you are not pregnant, you will register for the trial and be given a trial identification (ID) number. A doctor will then explain the endometrial biopsy procedure.

Before the procedure you will have a vaginal examination to find out the position of your womb so the healthcare professional can take the biopsy. The biopsy usually takes a couple of minutes. The healthcare professional will take the biopsy by passing a thin plastic tube through your cervix and into your womb. We will take a small sample of the lining of your womb. We will send part of this sample to the laboratory at the University Hospitals Coventry and Warwickshire NHS Trust (UHCW) to be analysed under the microscope to find out whether or not you have endometritis. With your permission, any tissue that is left over following analysis, and clinical data that does not identify you will be stored in Tommy's National Reproductive Health Biobank and used for future ethically approved research. This will not affect the sample sent to UHCW for analysis. The sample

For peer review only - http://bmjopen.bmj.com/site/about/guidelines.xhtml

CERM Chronic Endometritis and Recurrent Miscarriage

[Print on Trust Headed Paper]

sent to UHCW for analysis will be labelled with your trial ID number and initials, not your name.

The healthcare professional will do all they can to make sure they collect enough endometrial tissue to be analysed, but in a few cases this may not be possible. If the healthcare professional is not able to collect a sample or cannot collect enough tissue to be analysed, depending on the reason for this, the health professional carrying out the biopsy will discuss the following options with you.

- The same healthcare professional will repeat the biopsy during the same appointment
- A different healthcare professional will take the biopsy during the same appointment
- You come back to the clinic for another biopsy during your next menstrual cycle
- You have a biopsy taken at UHCW if you are happy and willing to travel to this site
- You choose not to repeat the biopsy and withdraw from the trial

After the biopsy you will be given a pregnancy test kit to take home.

5. **You get your biopsy results and the next steps** – While you are waiting for your biopsy results, it is important that you continue to use condoms whenever you have sex. We will phone you when your biopsy results are available. This will usually be within four weeks.

   If your results show that you do not have endometritis, you will continue with your usual care.
   - We will phone you at three, six and 12 months to see if you are still trying to get pregnant, if you are pregnant or if you have had any miscarriages or terminations. If you get pregnant between our phone calls, please let the hospital research team know (see the section: How can I contact the hospital trial team?).
   - If you get pregnant, we will collect follow-up information from the scans you have during your pregnancy.
   - If you have a baby, we will phone you eight weeks after the birth to ask about your health and your baby's health. The details we ask for will include the date of the birth, how many weeks pregnant you were at the time of the birth, how your baby was delivered, and your baby's weight and sex.
   - We will ask for details of any complications and abnormalities (for both you and your baby).

If your results show that you have endometritis, you may be eligible to take part in the randomised controlled trial. The following section explains what will happen during this stage. It is very important that you use condoms whenever you have sex during this entire menstrual cycle, and not just when you are taking the capsules.

For peer review only - http://bmjopen.bmj.com/site/about/guidelines.xhtml

CERM Chronic Endometritis and Recurrent Miscarriage

[Print on Trust Headed Paper]

Randomised controlled trial

A member of the hospital research team will phone you to explain that your results show you have endometritis and you may be eligible to take part in the randomised controlled trial. They will explain what this part of the trial is about and what taking part would mean for you. You can ask the hospital research team any questions you have.

What will happen if I agree to take part?

1. **We will check to see if you are still eligible.** We will make an appointment for you to have a consultation with a member of the hospital research team. The consultation may take place face-to-face or by phone or video call. You can ask any questions you have and you can ask your partner, a family member or a friend to join the consultation.

   During your consultation a doctor will ask you about your medical and obstetric history to check you are eligible to take part in the randomised controlled trial. If investigations have revealed a treatable cause for your recurrent miscarriage since you had the endometrial biopsy, you will not be eligible for the trial and a doctor will discuss your treatment options with you.

   An important part of the eligibility check is to find out if you are still willing to use condoms whenever you have sex throughout your entire menstrual cycle when you are taking the capsules.

   You will be asked to do a pregnancy test (using the pregnancy test kit provided at your biopsy visit) **and** call the hospital research team with the result. If the test shows you are pregnant, you will not be eligible for the trial and you will be referred to your GP who will arrange your care.

   Following the eligibility check, the doctor will ask you to confirm that you are happy to take part in the randomised controlled trial. You are free to withdraw from the trial at this stage or at any point, for any reason.

2. **Randomisation –** Whether you will receive doxycycline or the placebo is decided by chance. You and the trial researchers will not know who is taking the doxycycline and who is taking the placebo, but it will be possible to find this out if it becomes necessary for your clinical care.

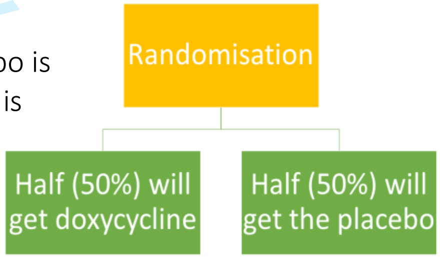

3. **Collecting your prescription and taking the capsules –** You can collect the capsules from a member of the hospital research team or from the hospital pharmacy (after you have collected your prescription from the hospital research team) at an agreed time that is convenient to you.

For peer review only - http://bmjopen.bmj.com/site/about/guidelines.xhtml

[Print on Trust Headed Paper]

You will be given an information booklet with the capsules. The booklet gives full instructions on how to take the capsules and you should read it carefully. It has guidance on what to do if something happens during the trial, and what precautions you should take while you are taking the capsules.

You can start taking the capsules on the first day of your next menstrual cycle. The dose is one capsule twice a day, 12 hours apart, for 14 days. Please fill in the treatment diary and mark a cross in the diary every time you take a capsule.

These capsules contain gelatine and small amounts of lactose.

4. You will be given a CERM trial participant card. It is very important that you carry your trial participant card with you at all times while you are taking the capsules, and show it to anyone you are receiving healthcare from. Throughout this entire menstrual cycle, it is important that you use condoms whenever you have sex.

5. If you miss your next period or suspect you are pregnant - Take a home pregnancy test. Follow the instructions that come with the test. Contact the hospital research team if the pregnancy test is positive or if you are not sure of the result.

Follow-up information

We will only collect information about you and your baby that is relevant to taking part in the CERM research trial. We will ask you for this information, but if you are not sure of, or don't know, the details we need we will refer to your hospital records (at whichever hospital you receive care from during the trial) or GP records (or both).

- We will phone you after you have taken all of your capsules to see if you have taken all of them as prescribed and if you have had any side effects. Please refer to your treatment diary for this information.
- We will phone you at three, six, 12 and 24 months plus at the end of the trial to see if you are still trying to get pregnant, if you are pregnant or if you have had any miscarriages or terminations. If you get pregnant between our phone calls, please let the hospital research team know (see the section: How can I contact the hospital trial team?).
- If you get pregnant, we will collect follow-up information from the scans you have during your pregnancy.
- If you have a baby, we will phone you eight weeks after the birth to ask about your health and your baby's health. The details we ask for will include the date of the birth, how many weeks pregnant you were at the time of the birth, how your baby was delivered, and your baby's weight and sex.
- We will ask for details of any complications, abnormalities, infections, investigations and results, treatment and hospital stays (for both you and your baby).

Expenses and payments

For peer review only - http://bmjopen.bmj.com/site/about/guidelines.xhtml

CERM Chronic Endometritis and Recurrent Miscarriage

[Print on Trust Headed Paper]

There are no payments for taking part in this research trial. We will refund any hospital parking charges you have to pay for hospital appointments that relate to this research.

What are the clinical alternatives?

Currently there are no clinical alternatives, as few treatments have been shown to prevent miscarriage.

What are the possible disadvantages and risks of taking part?

You and your partner will need to delay trying to conceive because it is important that you use condoms whenever you have sex throughout your entire menstrual cycles when you are:

- preparing for your biopsy;
- waiting for the result of your biopsy; and
- taking the capsules.

We have streamlined the trial processes to make sure the time you need to delay trying to conceive is as short as possible.

Some women find the endometrial biopsy is painful and may get cramping at the time of the biopsy. Taking paracetamol and ibuprofen an hour before the biopsy can help with this. If you need it, gas and air (Entonox) will be available while you are having the biopsy. Some women may have some vaginal bleeding (spotting) after the biopsy is taken. This will stop quickly on its own.

Please see the 'Taking my capsules on the chronic endometritis and recurrent miscarriage (CERM) trial' leaflet for details of any possible side effects while you are taking the capsules, and what to do if something happens when you are taking them.

What are the possible benefits of taking part?

We do not know if taking part in the research trial would benefit you personally. Taking part will show whether you have chronic endometritis. The results will help us provide advice on treatment options for women who have recurrent miscarriage.

What happens when the research trial stops?

The research trial is planned to take up to 48 months to complete.

Whenever the research trial stops, the university trial team will analyse the information collected to decide if antibiotics should be prescribed to women with chronic endometritis. In the future, these results will help women who are experiencing recurrent miscarriage.

This is the end of Part 1.

If you are interested in taking part in the trial, please read the important information in Part 2 before making your decision.

For peer review only - http://bmjopen.bmj.com/site/about/guidelines.xhtml

[Print on Trust Headed Paper]

Part 2

What if there is a problem?

If you have any concerns about any aspect of this study, you should ask to speak to the researchers who will do their best to answer your questions. If you are still not happy and want to make a complaint, you can do this through the NHS complaints procedure.

Please write to:

Complaints Manager
University Hospitals of Coventry and Warwickshire NHS Trust
Clifford Bridge Road
CV2 2DX

Or phone: 02476 965 198

In the unlikely event that you are harmed by taking part in this study, compensation may be available. If you suspect that the harm is the result of someone's negligence, you may be able to take legal action, but you may have to pay any costs involved and you should get legal advice about this.

For independent advice on research, you can contact PALS (Patient Advice and Liaison Service) on Freephone 0800 028 4203, or you can email them at feedback@uhcw.nhs.uk.

What Covid-19 precautions should I take when coming to hospital for the trial?

Your local NHS trust may have introduced measures to reduce your risk of being exposed to Covid-19. Please see the relevant hospital's website or ask the hospital trial team for more information.

Should I have the Covid-19 vaccine while I am taking part in the trial?

The CERM trial team, which includes clinicians and a pharmacist, have confirmed that there are no interactions between the drug used in the trial (doxycycline) and available Covid-19 vaccines. This means there are no safety concerns with you having a Covid-19 vaccine if you are offered one while you are taking part in the trial.

The hospital research team will ask you to let them know if you do have a Covid-19 vaccine so they can record the details alongside any other medications you are taking. If you have any questions, please get in touch with the hospital research team or your GP.

How will my information be used?

University Hospital Coventry and Warwickshire (UHCW) is the sponsor for this trial in the United Kingdom. The trial will be managed by Warwick Clinical Trials Unit at the University of Warwick (UoW). UHCW and the UoW will use information you provide and information from your hospital records and your GP records to carry out this trial, and will act as joint data

For peer review only - http://bmjopen.bmj.com/site/about/guidelines.xhtml

CERM Chronic Endometritis and Recurrent Miscarriage **[Print on Trust Headed Paper]**

controllers for the trial. This means that, together, they are responsible for looking after your information and using it properly.

We will only collect information about you and your baby that is relevant to taking part in the CERM research trial. We will ask you for this information, but if you are not sure of, or don't know, the details we need we will refer to your hospital records (at whichever hospital you receive care from during the trial) or GP records (or both). The information we will collect is listed below.

- The name of any investigations you have had
- The results of any investigations you have had
- The name of any illnesses, conditions or dependencies you have or have had
- The name of any prescription medications you are taking, the dose or units, and how long you have been taking them
- Details of any pregnancies, miscarriages or terminations
- The results of any pregnancy scans
- Details of any births, including the date, how many weeks pregnant you were at the time of the birth, how your baby was delivered, whether your baby was born alive or was stillborn, and your baby's weight and sex
- Details of any complications, abnormalities, infections, investigations and results, treatment and hospital stays (for both you and your baby)
- The results from the routine genetic analysis of any miscarriage tissue and tissue from the placenta (we will only collect this from you if you choose to take part in the randomised controlled trial)

Your hospital will collect information from you and your medical records and will pass this to us for this research trial in line with our instructions. This information will include your initials and date of birth. We would also like to collect details of your ethnic background and race, but you can choose not to provide this information if you prefer. We will ask for your permission to tell your GP that you are taking part in the research trial. If you do not want us to tell your GP, you will not be able to take part.

Your hospital will keep your name, NHS number and contact details confidential and will not pass this information to UHCW or the UoW. Your hospital will use this information, as needed, to contact you about the research trial, to make sure that relevant information about the trial is recorded for your care, and to oversee the quality of the trial. Certain people from UHCW, the UoW and regulatory organisations may look at your medical and research records to check that the research trial is accurate. UHCW and the UoW will only receive information that does not contain any details that directly identify you. The people who analyse the information will not be able to identify you and will not be able to find out your name, NHS number or contact details. UHCW and the UoW will keep identifiable information about you for 10 years after the trial has finished (if the results of your biopsy show that you **do not** have endometritis) or for 25 years after the trial has finished (if the results of your biopsy show that you **do** have endometritis).

For peer review only - http://bmjopen.bmj.com/site/about/guidelines.xhtml

[Print on Trust Headed Paper]

When you agree to take part in a research trial, the information about your health and care may be provided to researchers running other research studies in this organisation and in other organisations. These organisations may be universities, NHS organisations or companies involved in health and care research in this country or abroad. Organisations and researchers will only use your information to carry out research in line with the UK Policy Framework for Health and Social Care Research. This information will not identify you and will not be combined with other information in a way that could identify you. The information will only be used for the purpose of health and care research. It will not affect your care, and organisations and researchers cannot use it to contact you. Your information will not be used to make decisions about future services that are available to you, such as insurance.

Your rights to see, change or move your information are limited, as your information is managed in specific ways to make sure the research is reliable and accurate. If you withdraw from the trial, the information that has already been collected about you will be kept. To protect your rights, as few details as possible that could identify you will be collected.

To find out more about how your information is handled, you can visit the privacy notices of the data controllers.
www.uhcw.nhs.uk/privacy/
www.warwick.ac.uk/services/idc/dataprotection/privacynotices/researchprivacynotice

Will my information be kept confidential?

Yes. All information collected about you is strictly confidential. Any research information UHCW and the UoW collect will refer to you only by a unique trial ID number and your initials, so the risk of you being identified is very low. All your information will be stored securely and held at the Warwick Clinical Trials Unit, in line with all relevant UK laws.

What if new information becomes available?

If any new information about your treatment becomes available during the research trial, your doctor will discuss this with you.

What will happen if I don't want to carry on with the research trial?

If you do not want to continue in the research trial, you can withdraw at any time without giving a reason. This will not affect your care in any way. If you decide to withdraw from the research trial, you can choose to have no further contact from us.

What will happen to the results of this research trial?

At the end of the research trial, the university trial team will prepare and publish a report. The results of the research trial will be publicly available on the CERM trial website at www.warwick.ac.uk/cerm. The results will be available to the hospitals that took part in the research trial.

The results of the research trial may be presented at scientific meetings and published in scientific journals. The university trial team will also share the results of the research trial with the Royal College of Obstetricians and Gynaecologists, who publish guidance on the best care

For peer review only - http://bmjopen.bmj.com/site/about/guidelines.xhtml

CERM Chronic Endometritis and Recurrent Miscarriage **[Print on Trust Headed Paper]**

for women across the world. You will not be identified in any reports or publications and none of the information will be able to be traced to you personally.

Who is organising and funding this research trial?
This research trial is funded by the National Institute for Health Research (NIHR) in partnership with the Medical Research Council (MRC) under the Efficacy and Mechanism Evaluation Programme 17/60/22. The Government set up the NIHR in 2006 to provide organised funding for research within the NHS.

University Hospital Coventry and Warwickshire is sponsoring the research trial. This covers the insurance costs that apply to research trials. Professor Siobhan Quenby (from University Hospital Coventry, Warwickshire NHS Trust and The University of Warwick) is the Chief Investigator and has overall responsibility for the research trial. The University of Warwick Clinical Trials Unit is organising the administration of the research trial.

Who has reviewed this research trial?

1. Reviewed and commissioned by the National Institute for Health Research - Efficacy and Mechanism Evaluation Programme.
2. Reviewed and approved by North West-Haydock Research Ethics Committee (the REC) on 20/08/2019. The REC are an independent group of people who review all research carried out in the NHS to protect your safety, rights, well-being and dignity.
3. Reviewed and authorised by the Medicines and Healthcare products Regulatory Agency (MHRA) on 20/08/2019. The MHRA is the government body that authorises clinical trials of medicines.
4. Reviewed and approved by the Quality Assurance team at Warwick Clinical Trials Unit.
5. Reviewed and approved by the Research and Development Office at your local NHS trust.
6. Reviewed by the patient and public involvement (PPI) representative on this trial.

How can I contact the hospital trial team?
If you have any questions about the research trial or your involvement in it, either now or in the future, you can contact the hospital trial team.

Hospital trial team
Email: [insert number]
Phone: [insert number]
Write to: [insert number]

Information about the CERM trial, and other useful information, is available from the CERM trial website at www.warwick.ac.uk/cerm.

Thank you for taking the time to read this information sheet.

For peer review only - http://bmjopen.bmj.com/site/about/guidelines.xhtml

[Print on Trust Headed Paper]

DoH Disclaimer and **Funding acknowledgment** - This project (project reference 17/60/22) is funded by the Efficacy and Mechanism Evaluation (EME) Programme, an MRC and NIHR partnership. The views expressed in this publication are those of the authors and not necessarily those of the MRC, NIHR or the Department of Health and Social Care.

For peer review only - http://bmjopen.bmj.com/site/about/guidelines.xhtml

CERM Chronic Endometritis and Recurrent Miscarriage

[Print on Trust Headed Paper]

Crystal Mark 23136 Clarity approved by Plain English Campaign

Participant information sheet (CERM B)

Information for women taking part in the chronic endometritis and recurrent miscarriage (CERM) trial

Trial title

Chronic endometritis and recurrent miscarriage – the CERM trial

This information sheet is available in **large print** from the university trial team (email: cerm@warwick.ac.uk).

Introduction

You are invited to take part in a research trial. Please read this information sheet carefully before deciding whether to take part. It explains why this research trial is being done and what it means for you if you decide to take part. You will have the opportunity to talk to a member of the hospital research team about this trial and they will be happy to answer any questions you have. You can also discuss the trial with your GP or the obstetrician or gynaecologist caring for you.

This leaflet is divided into two parts. **Part 1** tells you the purpose of the trial and what will happen to you if you take part. **Part 2** gives you more detailed information about how the trial will be carried out.

Part 1

What is the purpose of the research trial?

The aim of this research trial is to find out if antibiotics can reduce miscarriage. In some women the lining of the womb (the endometrium) is inflamed. Researchers have found a link between this and miscarriage. A healthy endometrium is important for the embryo to be able to attach to the womb. It is thought that endometritis disrupts this process, and can lead to a miscarriage. Treating endometritis with antibiotics may reduce the inflammation and the likelihood of a miscarriage. This has not been tested. This research trial will test this theory by comparing a 14-day course of an antibiotic (doxycycline) against a placebo (a 'dummy treatment' which will look exactly the same as the antibiotic but contains no active ingredients) to find out if taking antibiotics reduces miscarriages. The trial will be 'double blind'. This means that the women and the trial researchers will not know who is taking the antibiotic capsules and who is taking the placebo. It will also be a randomised controlled trial. This means, if you decide to take part, which capsules you receive (the antibiotic or the placebo) will be decided by chance. The trial will take place in NHS hospitals in the United Kingdom and will involve over 3,000 women who have recurrent miscarriage.

Inflammation of the lining of the womb is a condition called **endometritis.**

Recurrent miscarriage means two or more miscarriages in a row.

For peer review only - http://bmjopen.bmj.com/site/about/guidelines.xhtml

[Print on Trust Headed Paper]

Not everyone who has recurrent miscarriage has endometritis, so before you can take part in the randomised controlled trial the hospital trial team will need to take an endometrial biopsy (a small sample of tissue from your endometrium) and examine it under a microscope to find out if you do. We (the university research team) estimate that half the women tested will have endometritis and so be able to take part in the randomised controlled trial.

Researchers also suspect that inflammation of the endometrium may be caused by an imbalance of the microbiome that lives in the reproductive tract (the vagina, cervix, womb, fallopian tubes and ovaries). Another part of the trial is to look at the endometrium and microbiome to see how antibiotics affect these. A diagram of this research trial is shown in figure 1.

The **microbiome** is the name given to all the microbes that live in and on our bodies. It is mostly made up of bacteria, and everyone's microbiome is unique.

For peer review only - http://bmjopen.bmj.com/site/about/guidelines.xhtml

CERM Chronic Endometritis and Recurrent Miscarriage

[Print on Trust Headed Paper]

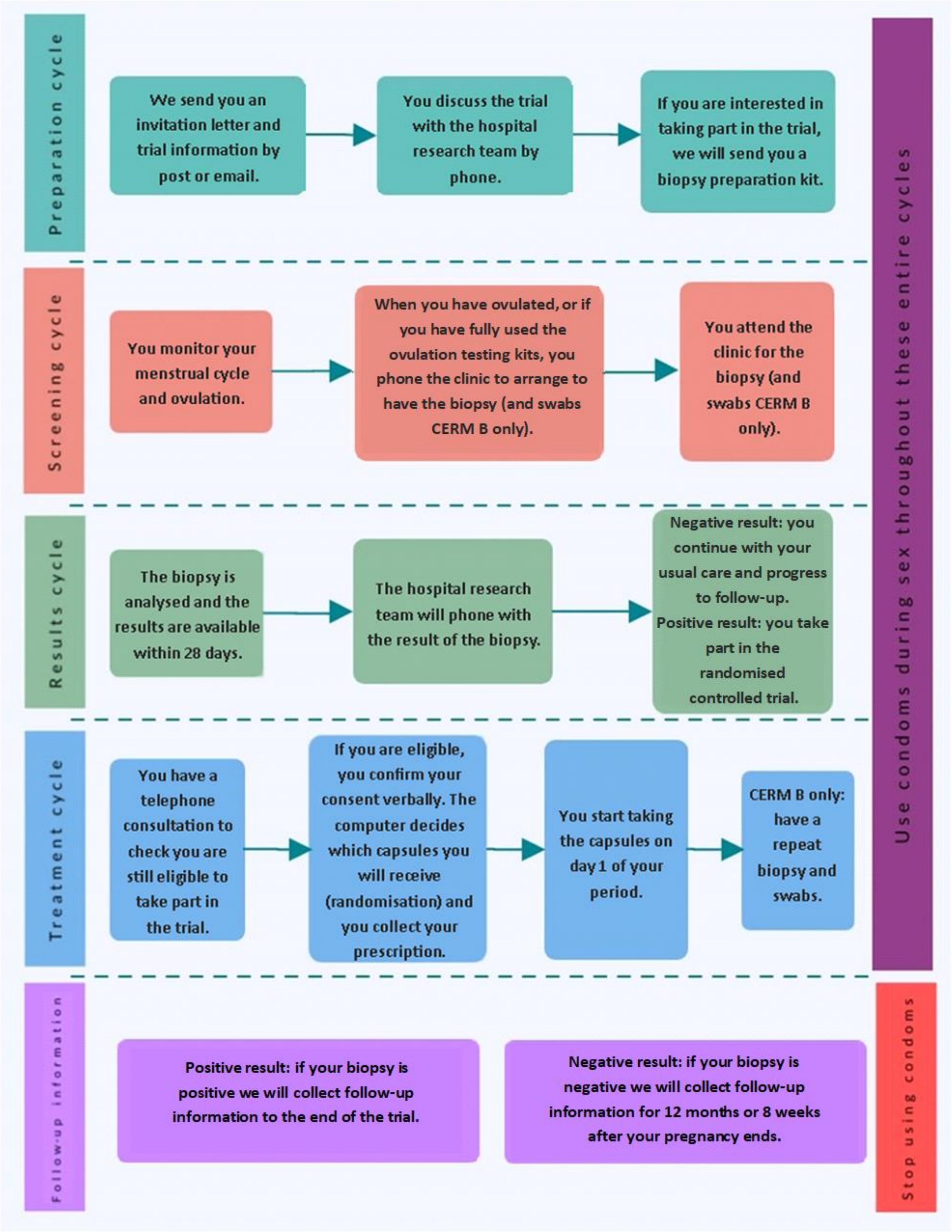

Figure 1 – an overview of the CERM trial

For peer review only - http://bmjopen.bmj.com/site/about/guidelines.xhtml

[Print on Trust Headed Paper]

Screening trial

Why have I been invited?

You have been invited to take part in this research trial because you have had two or more miscarriages (recurrent miscarriage).

Do I have to take part?

Taking part in the research trial is entirely voluntary. It is up to you whether or not you take part. You do not have to take part, and there will be no difference in the care you receive if you choose not to take part. If you want to take part, you will be asked to sign a consent form to confirm you have agreed to take part. You will keep a signed copy of the form. Even after signing the consent form, you can withdraw from the trial at any time if you change your mind, without having to give a reason. This will not affect the care you receive.

This information sheet will now explain what will happen if you decide to take part in this trial and have an endometrial biopsy and vaginal, cervical and endometrial swabs taken.

What will happen next?

A member of the hospital research team will talk to you over the phone (or speak to you face-to-face if you prefer this and if it is possible) to explain what the trial is about and what taking part would mean for you. You can ask the hospital research team any questions you have.

What will happen if I agree to take part?

1. **We will check to see if you are eligible –** You must be aged 18 to 41 years old. The hospital research team will ask about your obstetric and medical history to see if you are eligible to take part in the research trial. If you have not had any investigations to find out the cause of your recurrent miscarriage, these will be done when you attend the clinic. If a cause for your miscarriages is found, you will not be eligible for the trial. An important part of the eligibility check is to find out if you are willing to use condoms whenever you have sex throughout the entire menstrual cycles when you are:
    - preparing for your biopsy and swabs;
    - waiting for the result of your biopsy; and
    - taking the capsules and having your repeat biopsy and swabs.

    Doxycycline, the antibiotic used in the study, can be passed on in breast milk. The effects this could have on babies are not known. For this reason, and to make sure the study is absolutely safe, you will not be able to join the trial if you are breastfeeding.

2. **We will ask for permission to send you a 'biopsy preparation kit' –** If you are eligible to have a biopsy taken, the hospital research team will ask for your verbal consent to post a biopsy preparation kit to you. If you agree, this will be recorded in your hospital case notes and you will be given a trial screening number. The kit contains instructions, a period tracker, condoms and an ovulation testing kit. If you are prescribed a course of antibiotics between giving verbal consent and receiving the biopsy preparation kit, or

For peer review only - http://bmjopen.bmj.com/site/about/guidelines.xhtml

CERM Chronic Endometritis and Recurrent Miscarriage

[Print on Trust Headed Paper]

while you are using the biopsy preparation kit, please contact a member of the hospital research team (see the section: How can I contact the hospital trial team?). The biopsy may need to be delayed and the hospital team may need to send you another biopsy preparation kit once you have finished your course of antibiotics.

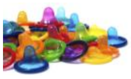

3. **Preparing for the biopsy and swabs –** It is very important that you **do not** try to conceive and that you use condoms whenever you have sex throughout this entire menstrual cycle. Condoms are provided in the kit and you can get more by contacting the hospital research team or family-planning services.

Starting on the first day of your next menstrual cycle after receiving the kit, you should use the period tracker to record:

- the days of your period; and
- the results of the ovulation testing.

The biopsy and swabs need to be taken a few days after you have ovulated. Ovulation usually happens around day 12 to 16, depending on the length of your cycle. We provide a period tracker and ovulation testing kit to help you work out the best time to have the biopsy and swabs taken. When the ovulation test shows you have ovulated, or if you have used all of the kit and it does not show you have ovulated, you will need to contact the hospital research team to arrange an appointment to come in for your biopsy and swabs.

4. **Having the biopsy and swabs –** Please bring your period tracker with you when you come to the clinic for your biopsy and swabs. In the clinic a doctor will explain what will happen and check you are eligible to take part in the trial. You will have the opportunity to ask them any questions you might have. You are welcome to bring your partner, a family member or a friend to this appointment, if the local rules on visitors to the clinic allow this. If you are eligible and would like to take part, you will be asked to sign a consent form. You will also be asked to take a pregnancy test before the biopsy. A healthcare professional will ask you for a sample of urine and they will test it. If the test shows you are pregnant, the biopsy and swabs will be cancelled, because it could put the pregnancy at risk, and you will be referred to your GP who will arrange for your care. If the pregnancy test shows you are not pregnant, you will register for the trial and be given a trial identification (ID) number. A doctor will then explain the endometrial biopsy procedure.

Before the procedure you will have a vaginal examination to find out the position of your womb so the healthcare professional can take the biopsy and swabs. The biopsy and swabs usually take a couple of minutes. First the healthcare professional will take swabs from your vagina, cervix and endometrium. They will send the swabs, and clinical data that does not identify you to the laboratory at Imperial College London, who will look at the microbiome to see what microbes are present. With your permission any material that is left over from the swabs, and clinical data that does not identify you, will be stored in the Tommy's National Reproductive Health Biobank. As this part of the trial is to look at how chronic endometritis may cause miscarriage, you will not get any results from these swabs.

For peer review only - http://bmjopen.bmj.com/site/about/guidelines.xhtml

[Print on Trust Headed Paper]

The healthcare professional will then take the biopsy by passing a thin plastic tube through your cervix and into your womb. We will take a small sample of the lining of your womb. We will send part of this sample to the laboratory at the University Hospitals Coventry and Warwickshire NHS Trust (UHCW) to be analysed under the microscope to find out whether or not you have endometritis. The sample sent to UHCW for analysis will be labelled with your trial ID number and initials, not your name. With your permission, any tissue that is left over following the analysis, and clinical data that does not identify you, will be stored in the Tommy's National Reproductive Health Biobank and used in future ethically approved research.

The healthcare professional will do all they can to make sure they collect enough endometrial tissue to be analysed, but in a few cases this may not be possible. If the healthcare professional is not able to collect a sample or cannot collect enough tissue to be analysed, depending on the reason for this, the health professional carrying out the biopsy will discuss the following options with you.

- The same healthcare professional will repeat the biopsy during the same appointment
- A different healthcare professional will take the biopsy during the same appointment
- You come back to the clinic for another biopsy during your next menstrual cycle
- You choose not to repeat the biopsy and withdraw from the trial

After the biopsy you will be given a pregnancy test kit to take home.

5. <u>You get your biopsy results and the next steps</u> – While you are waiting for your biopsy results, it is important that you continue to use condoms whenever you have sex. We will phone you when your biopsy results are available. This will usually be within four weeks.

   If your results show that you do not have endometritis, you will continue with your usual care.

   - We will phone you at three, six and 12 months to see if you are still trying to get pregnant, if you are pregnant or if you have had any miscarriages or terminations. If you get pregnant between our phone calls, please let the hospital research team know (see the section: How can I contact the hospital trial team?).
   - If you get pregnant, we will collect follow-up information from the scans you have during your pregnancy.
   - If you have a baby, we will phone you eight weeks after the birth to ask about your health and your baby's health. The details we ask for will include the date of the birth, how many weeks pregnant you were at the time of the birth, how your baby was delivered, and your baby's weight and sex.
   - We will ask for details of any complications and abnormalities (for both you and your baby).

If your results show that you have endometritis, you may be eligible to take part in the randomised controlled trial. The following section explains what will happen during this stage.

For peer review only - http://bmjopen.bmj.com/site/about/guidelines.xhtml

[Print on Trust Headed Paper]

It is very important that you use condoms whenever you have sex during this entire menstrual cycle, and not just when you are taking the capsules.

Randomised controlled trial

A member of the hospital research team will phone you to explain that your results show you have endometritis and you may be eligible to take part in the randomised controlled trial. They will explain what this part of the trial is about and what taking part would mean for you. You can ask the hospital research team any questions you have.

For peer review only

What will happen if I agree to take part?

1. **We will check to see if you are still eligible.** We will make an appointment for you to have a consultation with a member of the hospital research team. The consultation may take place face-to-face or by phone or video call. You can ask any questions you have and you can ask your partner, a family member or a friend to join the consultation.

   During your consultation a doctor will ask you about your medical and obstetric history to check you are eligible to take part in the randomised controlled trial. If investigations have revealed a treatable cause for your recurrent miscarriage since you had the endometrial biopsy, you will not be eligible for the trial and a doctor will discuss your treatment options with you.

   An important part of the eligibility check is to find out if you are still willing to use condoms whenever you have sex throughout your entire menstrual cycle when you are taking the capsules.

   You will be asked to do a pregnancy test (using the pregnancy test kit provided at your biopsy visit) **and** call the hospital research team with the result. If the test shows you are pregnant, you will not be eligible for the trial and you will be referred to your GP who will arrange your care.

   Following the eligibility check, the doctor will ask you to confirm that you are happy to take part in the randomised controlled trial. You are free to withdraw from the trial at this stage or any point, for any reason.

2. **Randomisation –** Whether you will receive doxycycline or the placebo is decided by chance. You and the trial researchers will not know who is taking the doxycycline and who is taking the placebo, but it will be possible to find this out if it becomes necessary for your clinical care.

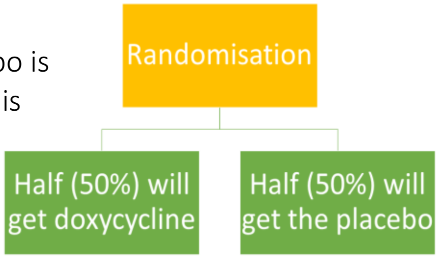

CERM Chronic Endometritis and Recurrent Miscarriage

[Print on Trust Headed Paper]

3. **Collecting your prescription and taking the capsules –** You can collect the capsules from a member of the hospital research team or from the hospital pharmacy (after you have collected your prescription from the hospital research team) at an agreed time that is convenient to you.

   You will be given an information booklet with the capsules. The booklet gives full instructions on how to take the capsules and you should read it carefully. It has guidance on what to do if something happens during the trial, and what precautions you should take while you are taking the capsules.

   You can start taking the capsules on the first day of your next menstrual cycle. The dose is one capsule twice a day, 12 hours apart, for 14 days. Please fill in the treatment diary and mark a cross in the diary every time you take a capsule.

   These capsules contain gelatine and small amounts of lactose.

   You will be given a CERM trial participant card. It is very important that you carry your trial participant card with you at all times while you are taking the capsules, and show it to anyone you are receiving healthcare from. Throughout this entire menstrual cycle, it is important that you use condoms whenever you have sex.

4. **Preparing for the repeat biopsy and swabs –** The procedure is identical to the first biopsy and swabs you had taken, and the instructions are repeated for you below. You can choose not to come for the second biopsy for any reason.

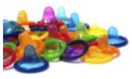

   It is very important that you **do not** try to conceive and you use condoms whenever you have sex throughout the entire menstrual cycle while you are taking the capsules and preparing for your repeat biopsy and swabs. Condoms were provided in the biopsy preparation kit and you can get more by contacting the hospital research team or family-planning services.

   Starting on the first day of your next menstrual cycle you should use the period tracker to record:
   a. the days of your period; and
   b. the results of the ovulation testing.

   The repeat biopsy and swabs need to be taken a few days after you have ovulated. Ovulation usually happens around day 12 to 16, depending on the length of your cycle. We provide a period tracker and ovulation testing kit to help you work out the best time to have the biopsy and swabs taken. When the ovulation test shows you have ovulated, or if you have used all of the kit and it does not show you have ovulated, you will need to contact the hospital research team to arrange an appointment to come in for your biopsy and swabs.

For peer review only - http://bmjopen.bmj.com/site/about/guidelines.xhtml

CERM Chronic Endometritis and Recurrent Miscarriage

[**Print on Trust Headed Paper**]

5. Having the repeat biopsy and swabs – Please bring your period tracker with you when you come to the clinic for your repeat biopsy and swabs. In the clinic a member of the hospital research team will explain what will happen. You will have the opportunity to ask them any questions you might have. You are welcome to bring your partner, a family member or a friend to this appointment, if the local rules on visitors to the clinic allow this. You will also be asked to take a pregnancy test. If the test shows you are pregnant, the biopsy and swabs will not be taken and details of your pregnancy will be recorded. If the pregnancy test shows you are not pregnant, a doctor will then explain the endometrial biopsy and swab procedure.

   Before the procedure you will have a vaginal examination to find out the position of your womb so the healthcare professional can take the biopsy and swabs. The biopsy and swabs usually take a couple of minutes. First the healthcare professional will take swabs from your vagina, cervix and endometrium. They will send the swabs, and clinical data that does not identify you, to the laboratory at Imperial College London, who will look at the microbiome to see what microbes are present. With your permission any material that is left over from the swabs, and clinical data that does not identify you, will be stored in the Tommy's National Reproductive Health Biobank.

   The healthcare professional will then take the biopsy by passing a thin plastic tube through your cervix and into your womb. We will take a small sample of the lining of your womb. We will send part of this sample to the laboratory at the University Hospitals Coventry and Warwickshire NHS Trust (UHCW) to be analysed under the microscope. The sample will be labelled with your trial ID number and initials, not your name. With your permission, any tissue that is left over following the analysis and clinical data that does not identify you, will be stored in the Tommy's National Reproductive Health Biobank and used in future ethically approved research. You will not receive the results of the biopsy or swabs, but they will allow us to find out the effect of the capsules.

   The healthcare professional will do all they can to make sure they collect enough endometrial tissue to be analysed, but in a few cases this may not be possible. If the healthcare professional is not able to collect a sample or cannot collect enough tissue to be analysed, the biopsy will not be performed again.

   When you attend for your repeat biopsy and swabs we will ask you if you have taken all of your capsules as prescribed and if you have had any side effects. Please bring your treatment diary and any leftover capsules to this visit.

6. If you miss your next period or suspect you are pregnant - Take a home pregnancy test. Follow the instructions that come with the test. Contact the hospital research team if the pregnancy test is positive or if you are unsure of the result.

Follow-up information

We will only collect information about you and your baby that is relevant to taking part in the CERM research trial. We will ask you for this information, but if you are not sure of, or don't

For peer review only - http://bmjopen.bmj.com/site/about/guidelines.xhtml

know, the details we need we will refer to your hospital records (at whichever hospital you receive care from during the trial) or GP records (or both).

- We will phone you after you have taken all of your capsules to see if you have taken all of them as prescribed and if you have had any side effects. Please refer to your treatment diary for this information.
- We will phone you at three, six, 12 and 24 months plus at the end of the trial to see if you are still trying to get pregnant, if you are pregnant or if you have had any miscarriages or terminations. If you get pregnant between our phone calls, please let the hospital research team know (see the section: How can I contact the hospital trial team?).
- If you get pregnant, we will collect follow-up information from the scans you have during your pregnancy.
- If you have a baby, we will phone you eight weeks after the birth to ask about your health and your baby's health. The details we ask for will include the date of the birth, how many weeks pregnant you were at the time of the birth, how your baby was delivered, and your baby's weight and sex.
- We will ask for details of any complications, abnormalities, infections, investigations and results, treatment and hospital stays (for both you and your baby).

Expenses and payments

There are no payments for taking part in this research trial. We will refund any hospital parking charges you have to pay for hospital appointments that relate to this research.

What are the clinical alternatives?

Currently there are no clinical alternatives, as few treatments have been shown to prevent miscarriage.

What are the possible disadvantages and risks of taking part?

You and your partner will need to delay trying to conceive because it is important that you use condoms whenever you have sex throughout your entire menstrual cycles when you are:

- preparing for your biopsy and swabs
- waiting for the result of your biopsy
- taking the capsules; and
- preparing for your repeat biopsy and swabs.

We have streamlined the trial processes to make sure the time you need to delay trying to conceive is as short as possible.

Some women find the endometrial biopsy is painful and may get cramping at the time of the biopsy. Taking paracetamol and ibuprofen an hour before the biopsy can help with this. If you need it, gas and air (Entonox) will be available while you are having the biopsy. Some women

For peer review only - http://bmjopen.bmj.com/site/about/guidelines.xhtml

[Print on Trust Headed Paper]

may have some vaginal bleeding (spotting) after the biopsy is taken. This will stop quickly on its own.

Please see the 'Taking my capsules on the chronic endometritis and recurrent miscarriage (CERM) trial' leaflet for details of any possible side effects while you are taking the capsules, and what to do if something happens when you are taking them.

What are the possible benefits of taking part?

We do not know if taking part in the research trial would benefit you personally. Taking part will show whether you have chronic endometritis. The results will help us provide advice on treatment options for women who have recurrent miscarriage.

What happens when the research trial stops?

The research trial is planned to take up to 48 months to complete.

Whenever the research trial stops, the university trial team will analyse the information collected to decide if antibiotics should be prescribed to women with chronic endometritis. In the future, these results will help women who are experiencing recurrent miscarriage.

This is the end of Part 1.

If you are interested in taking part in the trial, please read the important information in Part 2 before making your decision.

Part 2

What if there is a problem?

If you have any concerns about any aspect of this study, you should ask to speak to the researchers who will do their best to answer your questions. If you are still not happy and want to make a complaint, you can do this through the NHS complaints procedure.

Please write to:

Complaints Manager
University Hospitals of Coventry and Warwickshire NHS Trust
Clifford Bridge Road
CV2 2DX

Or phone: 02476 965 198

In the unlikely event that you are harmed by taking part in this study, compensation may be available. If you suspect that the harm is the result of someone's negligence, you may be able to take legal action, but you may have to pay any costs involved and you should get legal advice about this.

For peer review only - http://bmjopen.bmj.com/site/about/guidelines.xhtml

CERM Chronic Endometritis and Recurrent Miscarriage

[Print on Trust Headed Paper]

For independent advice on research, you can contact PALS (Patient Advice and Liaison Service) on Freephone 0800 028 4203, or you can email them at feedback@uhcw.nhs.uk.

What Covid-19 precautions should I take when coming to hospital for the trial?
Your local NHS trust may have introduced measures to reduce your risk of being exposed to Covid-19. Please see the relevant hospital's website or ask the hospital trial team for more information.

Should I have the Covid-19 vaccine while I am taking part in the trial?
The CERM trial team, which includes clinicians and a pharmacist, have confirmed that there are no interactions between the drug used in the trial (doxycycline) and available Covid-19 vaccines. This means there are no safety concerns with you having a Covid-19 vaccine if you are offered one while you are taking part in the trial.

The hospital research team will ask you to let them know if you do have a Covid-19 vaccine so they can record the details alongside any other medications you are taking. If you have any questions, please get in touch with the hospital research team or your GP.

How will my information be used?
University Hospitals Coventry and Warwickshire NHS Trust (UHCW) is the sponsor for this trial in the United Kingdom. The trial will be managed by Warwick Clinical Trials Unit at the University of Warwick (UoW). UHCW and the UoW will use information you provide and information from your hospital records and your GP records to carry out this trial, and will act as joint data controllers for the trial. This means that, together, they are responsible for looking after your information and using it properly.

We will only collect information about you and your baby that is relevant to taking part in the CERM research trial. We will ask you for this information, but if you are not sure of, or don't know, the details we need we will refer to your hospital records (at whichever hospital you receive care from during the trial) or GP records (or both). The information we will collect is listed below.

- The name of any investigations you have had
- The results of any investigations you have had
- The name of any illnesses, conditions or dependencies you have or have had
- The name of any prescription medications you are taking, the dose or units, and how long you have been taking them
- Details of any pregnancies, miscarriages or terminations
- The results of any pregnancy scans
- Details of any births, including the date, how many weeks pregnant you were at the time of the birth, how your baby was delivered, whether your baby was born alive or was stillborn, and your baby's weight and sex
- Details of any complications, abnormalities, infections, investigations and results, treatment and hospital stays (for both you and your baby)

For peer review only - http://bmjopen.bmj.com/site/about/guidelines.xhtml

CERM Chronic Endometritis and Recurrent Miscarriage

[Print on Trust Headed Paper]

- The results from the routine genetic analysis of any miscarriage tissue and tissue from the placenta (we will only record these if you choose to take part in the randomised controlled trial)

Your hospital will collect information from you and your medical records and will pass this to us for this research trial in line with our instructions. This information will include your initials and date of birth. We would also like to collect details of your ethnic background and race, but you can choose not to provide this information if you prefer. We will ask for your permission to tell your GP that you are taking part in the research trial. If you do not want us to tell your GP, you will not be able to take part.

Your hospital will keep your name, NHS number and contact details confidential and will not pass this information to UHCW or the UoW. Your hospital will use this information, as needed, to contact you about the research trial, to make sure that relevant information about the trial is recorded for your care, and to oversee the quality of the trial. Certain people from UHCW, the UoW and regulatory organisations may look at your medical and research records to check that the research trial is accurate. UHCW and the UoW will only receive information that does not contain any details that directly identify you. The people who analyse the information will not be able to identify you and will not be able to find out your name, NHS number or contact details. UHCW and the UoW will keep identifiable information about you for 10 years after the trial has finished (if the results of your biopsy show that you **do not** have endometritis) or for 25 years after the trial has finished (if the results of your biopsy show that you **do** have endometritis).

When you agree to take part in a research trial, the information about your health and care may be provided to researchers running other research studies in this organisation and in other organisations. These organisations may be universities, NHS organisations or companies involved in health and care research in this country or abroad. Organisations and researchers will only use your information to carry out research in line with the UK Policy Framework for Health and Social Care Research. This information will not identify you and will not be combined with other information in a way that could identify you. The information will only be used for the purpose of health and care research. It will not affect your care, and organisations and researchers cannot use it to contact you. Your information will not be used to make decisions about future services that are available to you, such as insurance.

Your rights to see, change or move your information are limited, as your information is managed in specific ways to make sure the research is reliable and accurate. If you withdraw from the trial, the information that has already been collected about you will be kept. To protect your rights, as few details as possible that could identify you will be collected.

To find out more about how your information is handled, you can visit the privacy notices of the data controllers.
www.uhcw.nhs.uk/privacy/
www.warwick.ac.uk/services/idc/dataprotection/privacynotices/researchprivacynotice

Will my information be kept confidential?

For peer review only - http://bmjopen.bmj.com/site/about/guidelines.xhtml

CERM Chronic Endometritis and Recurrent Miscarriage

[Print on Trust Headed Paper]

Yes. All information collected about you is strictly confidential. Any research information UHCW and the UoW collect will refer to you only by a unique trial ID number and your initials, so the risk of you being identified is very low. All your information will be stored securely and held at the Warwick Clinical Trials Unit, in line with all relevant UK laws.

What if new information becomes available?

If any new information about your treatment becomes available during the research trial, your doctor will discuss this with you.

What will happen if I don't want to carry on with the research trial?

If you do not want to continue in the research trial, you can withdraw at any time without giving a reason. This will not affect your care in any way. If you decide to withdraw from the research trial, you can choose to have no further contact from us.

What will happen to the results of this research trial?

At the end of the research trial, the university trial team will prepare and publish a report. The results of the research trial will be publicly available on the CERM trial website at www.warwick.ac.uk/cerm. The results will be available to the hospitals that took part in the research trial.

The results of the research trial may be presented at scientific meetings and published in scientific journals. The university trial team will also share the results of the research trial with the Royal College of Obstetricians and Gynaecologists, who publish guidance on the best care for women across the world. You will not be identified in any reports or publications and none of the information will be able to be traced to you personally.

Who is organising and funding this research trial?

This research trial is funded by the National Institute for Health Research (NIHR) in partnership with the Medical Research Council (MRC) under the Efficacy and Mechanism Evaluation Programme 17/60/22. The Government set up the NIHR in 2006 to provide organised funding for research within the NHS.

University Hospital Coventry and Warwickshire is sponsoring the research trial. This covers the insurance costs that apply to research trials. Professor Siobhan Quenby (from University Hospital Coventry, Warwickshire NHS Trust and The University of Warwick) is the Chief Investigator and has overall responsibility for the research trial. The University of Warwick Clinical Trials Unit is organising the administration of the research trial.

Who has reviewed this research trial?

1. Reviewed and commissioned by the National Institute for Health Research - Efficacy and Mechanism Evaluation Programme.
2. Reviewed and approved by North West-Haydock Research Ethics Committee (the REC) on 20/08/2019. The REC are an independent group of people who review all research carried out in the NHS to protect your safety, rights, well-being and dignity.

For peer review only - http://bmjopen.bmj.com/site/about/guidelines.xhtml

[Print on Trust Headed Paper]

3. Reviewed and authorised by the Medicines and Healthcare products Regulatory Agency (MHRA) on 20/08/2019. The MHRA is the government body that authorises clinical trials of medicines.
4. Reviewed and approved by the Quality Assurance team at Warwick Clinical Trials Unit.
5. Reviewed and approved by the Research and Development Office at your local NHS trust.
6. Reviewed by the patient and public involvement (PPI) representative on this trial.

How can I contact the hospital trial teams?

If you have any questions about the research trial or your involvement in it, either now or in the future, you can contact the hospital trial team.

Hospital trial team
Email: [insert number]
Phone: [insert number]
Write to: [insert number]

Information about the CERM trial, and other useful information, is available from the CERM trial website at www.warwick.ac.uk/cerm.

Thank you for taking the time to read this information sheet.

DoH Disclaimer and **Funding acknowledgment** - This project (project reference 17/60/22) is funded by the Efficacy and Mechanism Evaluation (EME) Programme, an MRC and NIHR partnership. The views expressed in this publication are those of the authors and not necessarily those of the MRC, NIHR or the Department of Health and Social Care.

For peer review only - http://bmjopen.bmj.com/site/about/guidelines.xhtml

Print on Trust headed paper

Participant consent form (CERM A)

Permission to take part in the chronic endometritis and recurrent miscarriage (CERM) trial

This form is available in **large print** from the trial team. Email **cerm@warwick.ac.uk**

Make three copies: Keep the original in the CERM trial investigator site file, give one copy to the participant, and put one in the participant's hospital medical notes.

Trial ID number					Principal investigator		Site	

**1.** I have read and understood the CERM A participant information sheet version [insert number] dated [dd/mm/yyyy].	Please initial
**2.** I have had time to think about the information I have been given about the CERM trial and ask questions, and I am satisfied with the answers I have been given.	Please initial
**3.** I understand that I do not have to take part and that I can withdraw from the CERM trial at any time, without giving a reason and without my medical care or legal rights being affected.	Please initial
**4.** I understand that the hospital research team will ask me for information about my past and current health that is relevant to me taking part in the CERM research trial. I give the hospital research team permission to collect and record this information.	Please initial
**5.** I understand that the hospital research team will collect information from my NHS hospital and GP records if it is relevant to me taking part in the CERM trial. I give the research team permission to access these records to collect and record this information.	Please initial
**6.** I understand that the research team and trial monitors from Warwick Clinical Trials Unit, the hospital trusts and regulatory authorities may have access to my medical and trial records to monitor this trial. I give permission for these people to access and record this information.	Please initial
**7.** I confirm that my partner and I have used condoms during this menstrual cycle. I will continue to use condoms whenever I am having sex throughout my entire menstrual cycle while I am waiting for the result of my biopsy and, if I take part in the randomised trial, while I am taking the capsules.	Please initial
**8.** I have seen the result of my pregnancy test taken today and confirm that it showed that I am not pregnant.	Please initial
**9.** I agree to have an endometrial biopsy taken.	Please initial
**10.** I agree to my endometrial biopsy being held in the Tommy's National Reproductive Health Biobank until it is analysed.	Please initial
**11. Optional:** I agree that any tissue that is left over following my endometrial biopsy or after my endometrial biopsy is analysed can be stored in the Tommy's National Reproductive Health Biobank, **with clinical data that does not identify me,** and used in future ethically approved research.	Please initial
**12.** I agree to the hospital trial team storing my phone numbers so they can contact me with the results of my endometrial biopsy and to discuss the next steps.	Please initial
**13.** I agree to the hospital trial team storing my phone numbers and contacting me for follow-up information as explained in the participant information sheet (or sheets).	Please initial
**14.** I agree to the research team telling my GP that I am taking part in the CERM research trial.	Please initial
**15.** I agree to take part in the CERM research trial.	Please initial

Please go to page 2.

Crystal Mark 23120 Clarity approved by Plain English Campaign

For peer review only - http://bmjopen.bmj.com/site/about/guidelines.xhtml

Print on Trust headed paper

We will only use the following consent if you later take part in the randomised controlled trial.

**16.** I understand that I should not take doxycycline while I am pregnant because it can affect the baby. I understand it is important to use condoms during my entire menstrual cycle when I am taking doxycycline or a placebo (whichever I am given).	Please initial
**17.** I agree to take doxycycline or a placebo for 14 days and only as instructed.	Please initial
**18.** I understand that if I give birth at another hospital, the hospital research team will contact the relevant hospital trust to collect medical details from my NHS hospital records (and my baby's) if this is relevant to taking part in the CERM research trial. I give the research team permission to access these records to collect and record this information.	Please initial
**19.** I understand that the hospital research team will collect information from my baby's NHS hospital and GP records if it is relevant to me taking part in the CERM trial. I give the research team permission to access these records to collect and record this information.	Please initial
**20.** I understand that the research team and trial monitors from Warwick Clinical Trials Unit, the hospital trusts and regulatory authorities may have access to my baby's medical and trial records to monitor this trial. I give permission for these people to access and record this information.	Please initial
**21.** I agree to take part in the CERM randomised controlled trial.	Please initial

Your name Please print **Your signature** Please sign **Date** dd/mm/yyyy

Investigator's name Please print **Investigator's signature** Please sign **Date** dd/mm/yyyy

Crystal Mark 23120 Clarity approved by Plain English Campaign

For peer review only - http://bmjopen.bmj.com/site/about/guidelines.xhtml

For peer review only

CERM Chronic Endometritis and Recurrent Miscarriage

Print on Trust headed paper

Participant consent form (CERM B)

Permission to take part in the chronic endometritis and recurrent miscarriage (CERM) trial

This form is available in **large print** from the trial team. Email **cerm@warwick.ac.uk**

Make three copies: Keep the original in the CERM trial investigator site file, give one copy to the participant, and put one in the participant's hospital medical notes.

Trial ID number					Principal investigator		Site	

**1.** I have read and understood the CERM B participant information sheet version [insert number] dated [dd/mm/yyyy].	Please initial
**2.** I have had time to think about the information I have been given about the CERM trial and ask questions, and I am satisfied with the answers I have been given.	Please initial
**3.** I understand that I do not have to take part and that I can withdraw from the CERM trial at any time, without giving a reason and without my medical care or legal rights being affected.	Please initial
**4.** I understand that the hospital research team will ask me for information about my past and current health that is relevant to me taking part in the CERM research trial. I give the hospital research team permission to collect and record this information.	Please initial
**5.** I understand that the hospital research team will collect information from my NHS hospital and GP records if it is relevant to me taking part in the CERM trial. I give the research team permission to access these records to collect and record this information.	Please initial
**6.** I understand that the research team and trial monitors from Warwick Clinical Trials Unit, the hospital trusts and regulatory authorities may have access to my medical and trial records to monitor this trial. I give permission for these people to access and record this information.	Please initial
**7.** I confirm that my partner and I have used condoms during this menstrual cycle. I will continue to use condoms whenever I am having sex throughout my entire menstrual cycle while I am waiting for the result of my biopsy and, if I take part in the randomised trial, while I am taking the capsules and preparing for my repeat biopsy and swabs.	Please initial
**8.** I have seen the result of my pregnancy test taken today and confirm that it showed that I am not pregnant.	Please initial
**9.** I agree to have an endometrial biopsy taken.	Please initial
**10.** I agree to my endometrial biopsy being held in the Tommy's National Reproductive Health Biobank until it is analysed.	Please initial
**11. Optional:** I agree that any tissue that is left over following my endometrial biopsy or after my endometrial biopsy is analysed can be stored in the Tommy's National Reproductive Health Biobank, with clinical data that does not identify me,- and used in future ethically approved research.	Please initial
**12.** I agree to have cervical, vaginal and endometrial swabs taken.	Please initial
**13.** I agree to my cervical, vaginal and endometrial swabs being stored in the Tommy's National Reproductive Health Biobank until they are analysed.	Please initial
**14. Optional:** I agree that any material that is left over after my cervical, vaginal and endometrial swabs are analysed can be stored in the Tommy's National Reproductive Health Biobank, with clinical data that does not identify me, and used in future ethically approved research.	Please initial

Please go to page 2.

Crystal Mark 23121 Clarity approved by Plain English Campaign

For peer review only - http://bmjopen.bmj.com/site/about/guidelines.xhtml

CERM Chronic Endometritis and Recurrent Miscarriage

Print on Trust headed paper

**15.** I agree to the hospital trial team storing my phone numbers so they can contact me with the results of my endometrial biopsy and to discuss the next steps.	Please initial
**16.** I agree to the hospital trial team storing my phone numbers and contacting me for follow-up information as explained in the participant information sheet (or sheets).	Please initial
**17.** I agree to the research team telling my GP that I am taking part in the CERM research trial.	Please initial
**18.** I agree to take part in the CERM research trial.	Please initial

We will only use the following consent if you later take part in the randomised controlled trial.

**19.** I understand that I should not take doxycycline while I am pregnant because it can affect the baby. I understand it is important to use condoms during my entire menstrual cycle when I am taking doxycycline or a placebo (whichever I am given).	Please initial
**20.** I agree to take doxycycline or a placebo for 14 days and only as instructed.	Please initial
**21.** I understand that if I give birth at another hospital, the hospital research team will contact the relevant hospital trust to collect medical details from my NHS hospital records (and my baby's) if this is relevant to taking part in the CERM research trial. I give the research team permission to access these records to collect and record this information.	Please initial
**22.** I understand that the hospital research team will collect information from my baby's NHS hospital and GP records if it is relevant to me taking part in the CERM trial. I give the research team permission to access these records to collect and record this information.	Please initial
**23.** I understand that the research team and trial monitors from Warwick Clinical Trials Unit, the hospital trusts and regulatory authorities may have access to my baby's medical and trial records to monitor this trial. I give permission for these people to access and record this information.	Please initial
**24.** I agree to have a pregnancy test taken when I come to hospital for a repeat endometrial biopsy. I understand I will be shown the result of the test to confirm I am not pregnant.	Please initial
**25.** I agree to have a repeat endometrial biopsy taken.	Please initial
**26.** I agree to my repeat endometrial biopsy being stored in the Tommy's National Reproductive Health Biobank until it is analysed.	Please initial
**27. Optional:** I agree that any tissue that is left over after my repeat endometrial biopsy is analysed can be stored in the Tommy's National Reproductive Health Biobank, **with clinical data that does not identify me,** and used in future ethically approved research.	Please initial
**28.** I agree to have repeat cervical, vaginal and endometrial swabs taken.	Please initial
**29.** I agree to my repeat cervical, vaginal and endometrial swabs being stored in the Tommy's National Reproductive Health Biobank until they are analysed.	Please initial
**30. Optional:** I agree that any material that is left over after my repeat cervical, vaginal and endometrial swabs are analysed can be stored in the Tommy's National Reproductive Health Biobank, **with clinical data that does not identify me,** and used in future ethically approved research.	Please initial
**31.** I agree to take part in the CERM randomised controlled trial.	Please initial

Please go to page 3.

Crystal Mark 23121 Clarity approved by Plain English Campaign

For peer review only - http://bmjopen.bmj.com/site/about/guidelines.xhtml

CERM
Chronic Endometritis and Recurrent Miscarriage

Print on Trust headed paper

Your name Please print	Your signature Please sign	Date dd/mm/yyyy
Investigator's name Please print	Investigator's signature Please sign	Date dd/mm/yyyy

For peer review only

Crystal Mark 23121 Clarity approved by Plain English Campaign
For peer review only - http://bmjopen.bmj.com/site/about/guidelines.xhtml

Possible side effects

If you get any of the following side effects, stop taking the capsules. Go to an emergency department and take your CERM trial participant card with you.

- Chest pain
- Difficulty breathing
- Swollen eyelids, face or lips
- Very severe headaches
- Severe skin reaction
- Fever or swollen lymph nodes
- Diarrhoea with blood in it

If you get any of the following side effects and you are concerned, call the hospital research team on [insert number].

- Feeling or being sick
- Heartburn
- Indigestion (dyspepsia)
- Gastritis (being sick)
- Vaginal infection

Please note: Very few women will get these side effects.

About the CERM trial

This is a new research trial designed to see if taking antibiotics reduces miscarriage.

If a woman has two or more miscarriages in a row, this is known as recurrent miscarriage. Some women who have recurrent miscarriage have an inflammation of the lining of the womb (chronic endometritis) and it is thought that this might prevent the embryo from attaching to the wall of the womb (implantation).

This trial is designed to find out if a two-week course of antibiotics will treat the inflammation and reduce the likelihood of a miscarriage.

During the trial, you will be given capsules to take each day. These will either contain an antibiotic (doxycycline) or a placebo (a substance with no medicinal effect, used as a control in clinical trials).

How to contact us

Email: <<<Insert site contact email>>>
Phone: <<<insert site number>>>
Address: <<<Insert Site Address>>>
Website: www.warwick.ac.uk/cerm

CERM
Chronic Endometritis and Recurrent Miscarriage

Crystal Mark 23107
Clarity approved by Plain English Campaign

Taking my capsules on the chronic endometritis and recurrent miscarriage (CERM) trial

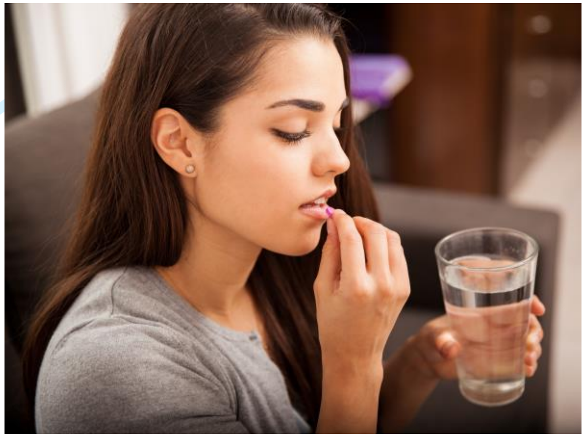

For peer review only - http://bmjopen.bmj.com/site/about/guidelines.xhtml

How to take the capsules

- Start taking the capsules on the first day of your period.
- Take **one** capsule **twice** a day.
- Swallow the capsules with plenty of water and take them with milk or food.
- Space the doses 12 hours apart and do not take them immediately before bedtime or if you are lying down.
- Keep taking this medicine until you finish the course, unless you are told to stop.
- Protect your skin from sunlight by using sunscreen, wearing appropriate clothing and spending time in the shade – even on a bright but cloudy day. Do not use sunbeds.
- **Do not** take indigestion remedies, or medicines which contain iron or zinc, two hours before or after you take this medicine.
- It is important to use condoms and not have unprotected sex during this entire menstrual cycle.
- It is important that you keep your CERM trial participant card with you at all times during the two weeks you are taking the capsules.

What to do if something happens when you are taking the capsules	
What if I forget to take a capsule?	Please do not double your dose. Take a capsule as soon as you remember, within six hours, and then take the next dose as scheduled. (If you don't remember until after six hours have passed, miss that dose – see below.) It is important that you finish the course.
What if I miss a capsule?	Please do not double your dose. Continue to take the capsules as prescribed and take the missed capsule at end of the course. It is important that you finish the course.
What if I miss more than one capsule?	Please do not double your dose. Continue to take the capsules as prescribed, and take the missed capsules at end of the course. It is important that you finish the course.
What if I lose my capsules?	If you lose your capsules we will not be able to replace them.
What if I take too many capsules?	Get medical advice immediately. Show your CERM trial participant card to any staff treating you. Stop taking the capsules. Tell the research team after your medical consultation.
What if I am sick after taking a capsule?	If you are sick within two hours of taking the capsule, you can take another one. If you are sick more than two hours after taking a capsule, take the next dose at the usual time.
What if I develop a rash?	If you develop a rash, stop taking the capsules and get medical advice. Show your CERM trial participant card to any staff treating you. Tell the research team after your medical consultation.
What if I have an allergic reaction?	If you have an allergic reaction, stop taking the capsules and get medical advice. Show your CERM trial participant card to any health staff treating you. Tell the research team after your consultation.
What if I find out I am pregnant when I'm taking the capsules?	If you find out you are pregnant while you are still taking the capsules, please stop taking them immediately and contact the research team. If you find you are pregnant after you have finished taking the capsules, please tell the research team.
Can I take the capsules with other medication?	Not all medications are safe to take with the capsules. If a health professional advises you to take a **new** medication, either prescribed or over the counter, show them your CERM trial participant card.
What if I get an infection and need to take antibiotics?	If you get an infection that needs antibiotics, show the healthcare provider your CERM trial patient card and follow their advice regarding stopping the trial capsules.
Can I drink alcohol when I am taking the capsules?	Do not drink alcohol when you are taking the capsules.
What if anything else happens?	If anything happens that is not listed here, call us for advice on [insert number]

For peer review only

SPIRIT | CONSORT OUTCOMES
Reporting Guidelines for Trial Outcomes

SPIRIT-Outcomes 2022 Checklist (for combined completion of SPIRIT 2013 and SPIRIT-Outcomes 2022 items)[a]

Section	Item No.	SPIRIT 2013 Item	SPIRIT-Outcomes 2022 item	Location Reported[b]
**Administrative information**				
Title	1	Descriptive title identifying the study design, population, interventions, and, if applicable, trial acronym	-	
Trial registration	2a	Trial identifier and registry name. If not yet registered, name of intended registry	-	
	2b	All items from the World Health Organization Trial Registration Data Set	-	
Protocol version	3	Date and version identifier	-	
Funding	4	Sources and types of financial, material, and other support	-	
Roles and responsibilities	5a	Names, affiliations, and roles of protocol contributors	-	
	5b	Name and contact information for the trial sponsor	-	
	5c	Role of study sponsor and funders, if any, in study design; collection, management, analysis, and interpretation of data; writing of the report; and the decision to submit the report for publication, including whether they will have ultimate authority over any of these activities	-	
	5d	Composition, roles, and responsibilities of the coordinating centre, steering committee, endpoint adjudication committee, data management team, and other individuals or groups overseeing the trial, if applicable (see Item 21a for data monitoring committee)	-	
**Introduction**				
Background and rationale	6a	Description of research question and justification for undertaking the trial, including summary of relevant studies (published and unpublished) examining benefits and harms for each intervention	-	
	6b	Explanation for choice of comparators	-	
Objectives	7	Specific objectives or hypotheses	-	

For peer review only

SPIRIT | CONSORT OUTCOMES
Reporting Guidelines for Trial Outcomes

Section	Item No.	SPIRIT 2013 Item	SPIRIT-Outcomes 2022 item	Location Reported[b]
Trial design	8	Description of trial design including type of trial (eg, parallel group, crossover, factorial, single group), allocation ratio, and framework (eg, superiority, equivalence, noninferiority, exploratory)	-	
**Methods: Participants, interventions, and outcomes**				
Study setting	9	Description of study settings (eg, community clinic, academic hospital) and list of countries where data will be collected. Reference to where list of study sites can be obtained	-	
Eligibility criteria	10	Inclusion and exclusion criteria for participants. If applicable, eligibility criteria for study centres and individuals who will perform the interventions (eg, surgeons, psychotherapists)	-	
Interventions	11a	Interventions for each group with sufficient detail to allow replication, including how and when they will be administered (for specific guidance see TIDieR checklist and guide)	-	
	11b	Criteria for discontinuing or modifying allocated interventions for a given trial participant (eg, drug dose change in response to harms, participant request, or improving/worsening disease)	-	
	11c	Strategies to improve adherence to intervention protocols, and any procedures for monitoring adherence (eg, drug tablet return, laboratory tests)	-	
	11d	Relevant concomitant care and interventions that are permitted or prohibited during the trial	-	
Outcomes	12	Primary, secondary, and other outcomes, including the specific measurement variable (eg, systolic blood pressure), analysis metric (eg, change from baseline, final value, time to event), method of aggregation (eg, median, proportion), and time point for each outcome. Explanation of the clinical relevance of chosen efficacy and harm outcomes is strongly recommended	-	

For peer review only

Section	Item No.	SPIRIT 2013 Item	SPIRIT-Outcomes 2022 item	Location Reported[b]
	12.1		Provide a rationale for the selection of the domain for the trial's primary outcome	
	12.2		If the analysis metric for the primary outcome represents within-participant change, define and justify the minimal important change in individuals	
	12.3		If the outcome data collected are continuous but will be analyzed as categorical (method of aggregation), specify the cutoff values to be used	
	12.4		If outcome assessments will be performed at several time points after randomization, state the time points that will be used for analysis	
	12.5		If a composite outcome is used, define all individual components of the composite outcome	
Participant timeline	13	Time schedule of enrolment, interventions (including any run-ins and washouts), assessments, and visits for participants. A schematic diagram is highly recommended (see Figure)	-	
Sample size	14	Estimated number of participants needed to achieve study objectives and how it was determined, including clinical and statistical assumptions supporting any sample size calculations	-	
	14.1		Define and justify the target difference between treatment groups (eg, the minimal important difference)	
Recruitment	15	Strategies for achieving adequate participant enrolment to reach target sample size	-	
**Methods: Assignment of interventions (for controlled trials)**				
Allocation:				
Sequence generation	16a	Method of generating the allocation sequence (eg, computer-generated random numbers), and list of any factors for stratification. To reduce predictability of a random sequence, details of any planned restriction (eg, blocking) should be provided in a separate document that is unavailable to those who enrol participants or assign interventions	-	

For peer review only - http://bmjopen.bmj.com/site/about/guidelines.xhtml

Section	Item No.	SPIRIT 2013 Item	SPIRIT-Outcomes 2022 item	Location Reported[b]
Allocation concealment mechanism	16b	Mechanism of implementing the allocation sequence (eg, central telephone; sequentially numbered, opaque, sealed envelopes), describing any steps to conceal the sequence until interventions are assigned	-	
Implementation	16c	Who will generate the allocation sequence, who will enrol participants, and who will assign participants to interventions	-	
Blinding (masking)	17a	Who will be blinded after assignment to interventions (eg, trial participants, care providers, outcome assessors, data analysts), and how	-	
	17b	If blinded, circumstances under which unblinding is permissible, and procedure for revealing a participant's allocated intervention during the trial	-	
**Methods: Data collection, management, and analysis**				
Data collection methods	18a	Plans for assessment and collection of outcome, baseline, and other trial data, including any related processes to promote data quality (eg, duplicate measurements, training of assessors) and a description of study instruments (eg, questionnaires, laboratory tests) along with their reliability and validity, if known. Reference to where data collection forms can be found, if not in the protocol	-	
	18a.1		Describe what is known about the responsiveness of the study instruments in a population similar to the study sample	
	18a.2		Describe who will assess the outcome (eg, nurse, parent)	
	18b	Plans to promote participant retention and complete follow-up, including list of any outcome data to be collected for participants who discontinue or deviate from intervention protocols	-	

For peer review only - http://bmjopen.bmj.com/site/about/guidelines.xhtml

SPIRIT | CONSORT OUTCOMES Reporting Guidelines for Trial Outcomes

Section	Item No.	SPIRIT 2013 Item	SPIRIT-Outcomes 2022 item	Location Reported[b]
Data management	19	Plans for data entry, coding, security, and storage, including any related processes to promote data quality (eg, double data entry; range checks for data values). Reference to where details of data management procedures can be found, if not in the protocol	-	
Statistical methods	20a	Statistical methods for analysing primary and secondary outcomes. Reference to where other details of the statistical analysis plan can be found, if not in the protocol	-	
	20a.1		Describe any planned methods to account for multiplicity in the analysis or interpretation of the primary and secondary outcomes (eg, coprimary outcomes, same outcome assessed at multiple time points, or subgroup analyses of an outcome)	
	20b	Methods for any additional analyses (eg, subgroup and adjusted analyses)	-	
	20c	Definition of analysis population relating to protocol non-adherence (eg, as randomised analysis), and any statistical methods to handle missing data (eg, multiple imputation)	-	
**Methods: Monitoring**				
Data monitoring	21a	Composition of data monitoring committee (DMC); summary of its role and reporting structure; statement of whether it is independent from the sponsor and competing interests; and reference to where further details about its charter can be found, if not in the protocol. Alternatively, an explanation of why a DMC is not needed	-	
	21b	Description of any interim analyses and stopping guidelines, including who will have access to these interim results and make the final decision to terminate the trial	-	
Harms	22	Plans for collecting, assessing, reporting, and managing solicited and spontaneously reported adverse events and other unintended effects of trial interventions or trial conduct	-	

For peer review only

Section	Item No.	SPIRIT 2013 Item	SPIRIT-Outcomes 2022 item	Location Reported[b]
Auditing	23	Frequency and procedures for auditing trial conduct, if any, and whether the process will be independent from investigators and the sponsor	-	
**Ethics and dissemination**				
Research ethics approval	24	Plans for seeking research ethics committee/institutional review board (REC/IRB) approval	-	
Protocol amendments	25	Plans for communicating important protocol modifications (eg, changes to eligibility criteria, outcomes, analyses) to relevant parties (eg, investigators, REC/IRBs, trial participants, trial registries, journals, regulators)	-	
Consent or assent	26a	Who will obtain informed consent or assent from potential trial participants or authorised surrogates, and how (see Item 32)	-	
	26b	Additional consent provisions for collection and use of participant data and biological specimens in ancillary studies, if applicable	-	
Confidentiality	27	How personal information about potential and enrolled participants will be collected, shared, and maintained in order to protect confidentiality before, during, and after the trial	-	
Declaration of interests	28	Financial and other competing interests for principal investigators for the overall trial and each study site	-	
Access to data	29	Statement of who will have access to the final trial dataset, and disclosure of contractual agreements that limit such access for investigators	-	
Ancillary and post-trial care	30	Provisions, if any, for ancillary and post-trial care, and for compensation to those who suffer harm from trial participation	-	
Dissemination policy	31a	Plans for investigators and sponsor to communicate trial results to participants, healthcare professionals, the public, and other relevant groups (eg, via publication, reporting in results databases, or other data sharing arrangements), including any publication restrictions	-	
	31b	Authorship eligibility guidelines and any intended use of professional writers	-	

For peer review only - http://bmjopen.bmj.com/site/about/guidelines.xhtml

SPIRIT | CONSORT OUTCOMES
Reporting Guidelines for Trial Outcomes

Section	Item No.	SPIRIT 2013 Item	SPIRIT-Outcomes 2022 item	Location Reported[b]
	31c	Plans, if any, for granting public access to the full protocol, participant-level dataset, and statistical code	-	
**Appendices**				
Informed consent materials	32	Model consent form and other related documentation given to participants and authorised surrogates	-	
Biological specimens	33	Plans for collection, laboratory evaluation, and storage of biological specimens for genetic or molecular analysis in the current trial and for future use in ancillary studies, if applicable	-	

[a]It is strongly recommended that this checklist be read in conjunction with the SPIRIT (Standard Protocol Items: Recommendations for Interventional Trials) Statement paper for important clarification on the items. Amendments to the protocol should be tracked and dated. The SPIRIT checklist is copyrighted by the SPIRIT Group under the Creative Commons "Attribution-NonCommercial-NoDerivs 3.0 Unported" license and is reproduced with permission.
[b]Indicates page numbers and/or manuscript location: to be completed by authors.

For peer review only

BMJ Open

Pre-conceptual administration of doxycycline in women with recurrent miscarriage and chronic endometritis: protocol for the Chronic Endometritis and Recurrent Miscarriage (CERM) trial, a multicentre, double-blind, placebo-controlled, adaptive randomised trial with an embedded translational sub-study

Journal:	*BMJ Open*
Manuscript ID	bmjopen-2023-081470.R1
Article Type:	Protocol
Date Submitted by the Author:	15-Nov-2023
Complete List of Authors:	Odendaal, Joshua; University of Warwick; University Hospitals Coventry and Warwickshire NHS Trust<br>Black, Naomi; University of Warwick; University Hospitals Coventry and Warwickshire NHS Trust,<br>Bouliotis, Georgios; University of Warwick<br>Guck, Jonathan; University of Warwick<br>Underwood, Martin; University of Warwick, Warwick Medical School<br>Fisher, Joanne; University of Warwick<br>Quenby, Siobhan; University of Warwick, Division of Reproductive Health, Warwick Medical School; University Hospitals Coventry and Warwickshire NHS Trust
**Primary Subject Heading**:	Obstetrics and gynaecology
Secondary Subject Heading:	Evidence based practice
Keywords:	Reproductive medicine < GYNAECOLOGY, Randomized Controlled Trial, Clinical Trial

SCHOLARONE™
Manuscripts

Pre-conceptual administration of doxycycline in women with recurrent miscarriage and chronic endometritis: protocol for the Chronic Endometritis and Recurrent Miscarriage (CERM) trial, a multicentre, double-blind, placebo-controlled, adaptive randomised trial with an embedded translational sub-study

Joshua Odendaal MRCOG PhD [1,2], Naomi Black MBBS MSc[1,2], Georgios Bouliotis PhD[3], Jonathan Guck MSc[3], Martin Underwood FRCGP MD[2,3], Joanne Fisher PhD[3], Siobhan Quenby FRCOG MD[1,2]

[1]Division of Biomedical Sciences, Clinical Sciences Research Laboratories, Warwick Medical School, University of Warwick, Coventry CV2 2DX, United Kingdom

[2]University Hospitals Coventry & Warwickshire, Coventry CV2 2DX, United Kingdom

[3]Warwick Clinical Trials Unit, Warwick Medical School, University of Warwick, Gibbett Hill, Coventry, CV4 7AL, United Kingdom

Correspondence to:

Joshua Odendaal

Division of Biomedical Sciences, Clinical Sciences Research Laboratories, Warwick Medical School, University of Warwick, Coventry CV2 2DX, United Kingdom.

Email: joshua.odendaal@warwick.ac.uk

ORCID ID: 0000-0001-7829-3729

Word Count: 3,989 words

For peer review only - http://bmjopen.bmj.com/site/about/guidelines.xhtml

Abstract

Introduction Recurrent miscarriage is a common condition with a substantial associated morbidity. A hypothesised cause of recurrent miscarriage is chronic endometritis (CE). The aetiology of CE remains uncertain. An association between CE and recurrent miscarriage has been shown. This study will aim to determine if pre-conceptual administration of doxycycline, in women with recurrent miscarriages, and chronic endometritis, reduces first trimester miscarriages, increasing live births.

Methods and analysis CERM is a multicentre, double-blind adaptive trial with an embedded translational sub-study. Women with a history of two or more consecutive first trimester losses with evidence of CE on endometrial biopsy (defined as ≥5 CD138 positive cells per $10mm^2$) will be randomised to oral doxycycline or placebo for 14-days. A subset will be recruited to a mechanistic sub-study in which microbial swabs and pre-/post-intervention endometrial samples will be collected. Up to 3062 women recruited from 29 NHS hospital sites across the United Kingdom are expected to be screened with up to 1500 women randomised in a 1:1 ratio. Women with a negative endometrial biopsy (defined as <5 CD138 positive cells per $10mm^2$) will also be followed up to test validity of the tool. The primary outcome is live births plus pregnancies ≥24 + 0 weeks gestation at the end of the trial, in the first or subsequent pregnancy. Secondary clinical outcomes will also be assessed. Exploratory outcomes will assess the effect of doxycycline treatment on the endometrial microbiota, the differentiation capacity of the endometrium and the senescent profile of the endometrium with CE.

Ethics and dissemination Ethical approval has been obtained from the NHS Research Ethics Committee Northwest- Haydock (19/NW/0462). Written informed consent will be gained from all participants. The results will be published in an open-access peer-reviewed journal and reported in the NIHR journals library.

Trial registration: ISRCTN23947730.

For peer review only - http://bmjopen.bmj.com/site/about/guidelines.xhtml

Strengths and limitations of this study

- This will be the largest randomised controlled trial assessing the role of antibiotics in improving livebirth rates in women with a history of recurrent miscarriage.
- An integrated mechanistic design allows elucidation of both clinical and mechanistic outcomes.
- Generation of a bank of data-linked samples for further chronic endometritis (CE) research.
- Recruitment may be difficult given the use of CE testing, relative low cost of doxycycline and treatment in the private sector and the limited equipoise within the specialist population.
- A large number of secondary outcomes with reduced power for detection in this model, but balanced by the increased benefits of an integrated design.

For peer review only

For peer review only - http://bmjopen.bmj.com/site/about/guidelines.xhtml

INTRODUCTION

Recurrent miscarriage is an important cause of reproductive associated morbidity. Defined as the loss of two or more pregnancies it has a population prevalence of up to 3% (1). It is research priority within reproductive health (2, 3). A *Lancet* review series highlighted the ongoing uncertainty around the aetiology of recurrent miscarriage (1). It is hypothesised that chronic endometritis (CE) is a cause of recurrent miscarriage (4). In chronic endometritis, there is asymptomatic inflammation of the endometrium mediated by infiltration of the endometrial stroma by plasma cells(5). CE remains of uncertain aetiology being differentially attributed to dysbiosis of the endometrial microbiota and the presence of pathological species within the endometrium (6-8). There is not consensus around the diagnosis of CE (9). Traditionally CE has been diagnosed on histology through the identification of infiltrating endometrial plasma cells (10). Recently this has been augmented using CD138 immunohistochemistry (11). Whilst this has resulted in improved sensitivity, the diagnostic thresholds remain variable and the impact on therapeutic outcome varies dependent on the CD138 threshold set (9). Liu et al., evaluated different diagnostic methodologies demonstrating a technique based on whole section density to have the lowest intra- and inter-observer variability (11). The study also identified a density threshold based on the 95% confidence interval with a differential prevalence of 22.2% in those with reproductive failure in comparison to 5% in fertile controls ($p<0.01$). McQueen et al., assessed the effect of antibiotic treatment in a cohort of women presenting with recurrent miscarriage or loss of a single fetus >10 weeks gestation (12). Following treatment with antibiotics, a similar cumulative livebirth rate (LBR) was seen in the CE group in comparison to controls (88% treated CE vs. 74% without CE; $p=0.215$). A significant increase in per pregnancy livebirth rate pre- and post- treatment for CE was also seen (7% pre-treatment LBR vs. 56% post treatment LBR; $p<0.001$). Similarly, a retrospective cohort study assessing for the presence of CE demonstrated a marked improvement in live birth rate in those with CE resolution following antibiotic therapy (78.4% LBR in resolved CE vs. 17.5% LBR persistent CE; $p<0.001$) (13). Doxycycline is the most commonly reported antibiotic therapy used in the treatment of CE. A single course has previously been reported to achieve histological resolution in 92% of cases (14). These findings, however, remain limited by sample size and methodological concerns including lack of randomisation and lack of untreated controls. Despite this however, commercial testing is now available within the private sector (15). Given the combined limitations in pathophysiological and clinical understanding of the condition a novel translation approach is required. In view of this, the Chronic Endometritis and Recurrent Miscarriage (CERM) trial was developed as a novel translation approach to better determine the role of CE in recurrent miscarriage and the potential for therapeutic intervention.

For peer review only - http://bmjopen.bmj.com/site/about/guidelines.xhtml

Study objectives

1. To determine if doxycycline administered prior to conception improves pregnancy outcome in women with recurrent miscarriage associated with chronic endometritis,

2. To explore the mechanisms by which it could prevent miscarriage, and

3. To determine if CD138 values predict miscarriage

Primary outcome

The primary outcome is total live births plus ongoing pregnancies ≥24 + 0 weeks gestation at the end of the trial, in the first or subsequent pregnancy. This measure was chosen as the most meaningful outcome to patients.

Secondary outcomes

Given aetiological uncertainty a cluster of both clinical and pathophysiological secondary outcomes have been selected. These include: Ongoing pregnancy at 12 weeks gestation, time to first conception, anticipated time to first livebirth, the proportion with livebirth ≥24 weeks gestation in the first pregnancy, pregnancy complications, type of miscarriage, ongoing pregnancy rates, livebirth rates in those excluded from randomisation as CE negative, the predictive value of CD138+ cell density on pregnancy outcome, the impact of intervention on endometrial $CD138^+$ cell density, and termination for social reasons.

Exploratory outcomes

Given the translational nature of the trial, a cluster of exploratory outcomes will also be assessed. These include an assessment of the effect of doxycycline treatment on the endometrial microbiota, the differentiation capacity of the endometrium and the senescent profile of the endometrium with CE.

METHODS AND ANALYSIS

The protocol (V8.3 22 June 2023) was designed in accordance with Standard Protocol Items: Recommendations for Interventional Trials guidelines (16). The trial has been prospectively registered on the ISRCTN registry (ISRCTN23947730).

Study design

The CERM trial is a multicentre, double blind adaptive trial. It is a multi-phased trial with a pre-eligibility phase, screening phase, randomisation phase and two separate follow-up pathways

For peer review only - http://bmjopen.bmj.com/site/about/guidelines.xhtml

dependent on-screen status. These are summarised in figure 1. This is a trial of adaptive design with three prespecified interim stops to assess for futility at 100, 300 and 900 participants. Based on the unblinding results, the trial may stop after Data Monitoring Committee (DMC) recommendation, if there is low or negligible treatment efficacy. In addition, the use of on-going pregnancy as a surrogate marker will also increase the trial flexibility whilst maintaining evidential rigour. The trial also has a parallel translational sub-study assessing the endometrial and microbial effects of a diagnosis of CE and the impact of treatment on this. Utilising the clinical trial to develop a sample cohort will allow assessment of both the exploratory outcomes and allow further basic science assessment of the impact of CE with the aim of identifying the pathophysiological sequence of the condition alongside the potential mechanism of treatment effect.

The clinical trial is conducted and managed by the Warwick Clinical Trials Unit (WCTU). The trial has been sponsored by University Hospitals Coventry and Warwickshire (UHCW). The trial is funded by the Efficacy and Mechanism Evaluation (EME) Programme, an MRC and NIHR partnership, project number 17/60/22. Standard Protocol Items: Recommendations for Interventional Trials reporting guidelines were used for this protocol (16).

Trial setting

The initial aim of the trial was to recruit up to 3062 women to the screening phase with an expected up to 1500 women randomised from 10 tertiary recurrent miscarriage centres. Given the decentralisation of recurrent miscarriage care however, a decision was taken to widen participation allowing trial set-up across all NHS trusts with a working recurrent miscarriage service. Participants will therefore be recruited from 29 NHS hospital sites located across the United Kingdom including England, Scotland and Wales.

Translational sub-study

Women recruited at the sponsor site will be entered into the optional translational sub-study. These women will undertake a dual consent process for both the clinical trial and for samples and associated clinical data to be banked within a reproductive health biobank allowing both flexibility and regulatory oversight of additional scientific exploratory work that emerges from endometrial assessment.

Women entered into the translational sub-study will undergo sampling of the microbiota in addition to endometrial histological sampling. This will be achieved through the concomitant collection of high vaginal swabs, endocervical swabs and uterine swabs alongside an outpatient endometrial biopsy. Consent to the sub-study will be undertaken by the research team at the sponsor site.

For peer review only - http://bmjopen.bmj.com/site/about/guidelines.xhtml

Environmental and negative controls will be collected across each collection day to adjust for potential contamination effects. Women who are screen positive will be offered repeat endometrial sampling in the intervention cycle to ascertain the endometrial effects of treatment. Overall, up to 200 paired samples will be obtained. A random sample of high vaginal and cervical swabs will be analysed to ascertain risk of contamination, the remainder will be stored. All endometrial swabs will be analysed.

Participants and recruitment

Eligibility for inclusion is dependent on the phase of the trial. The key entry point and assessment of eligibility is within the screening phase. These inclusion and exclusion criteria are as below.

Inclusion criteria

Women aged ≥18 to <42 who have experienced two or more consecutive first trimester miscarriages, with first trimester miscarriage defined as an intrauterine pregnancy loss at ≤14 weeks gestation. Women who have suffered an ectopic pregnancy, a molar pregnancy or 2nd trimester miscarriage between or after their first trimester miscarriages will still be eligible given the differing pathology of loss.

Exclusion criteria

Women will be excluded if they experienced a livebirth, stillbirth or termination of pregnancy between or after their first trimester miscarriage. In addition, women with a known treatable cause of RM will be excluded including antiphospholipid syndrome or uncontrolled thyroid disease. Women with a menstrual cycle <21 or >42 days will be excluded due to difficulties in timing the sampling and/or treatment. Those with systemic inflammatory conditions including Systemic Lupus Erythematous and Myasthenia Gravis will be excluded. Finally, those on long term antibiotics, having undertaken antibiotic therapy in the screening menstrual cycle or with a contraindication to doxycycline will be excluded.

Trial procedures

Pre-eligibility phase

Women eligible as above will be advised to avoid pregnancy and provided with ovulation tests. They will be verbally consented to the study by a clinician, trained research nurse or midwife. In a subsequent menstrual cycle, during which any intercourse involved use of barrier contraception, they will undertake ovulation testing and contact the research unit at the point of ovulation to book the screening biopsy.

For peer review only - http://bmjopen.bmj.com/site/about/guidelines.xhtml

For peer review only

Screening for chronic endometritis

A medical doctor will confirm eligibility and obtain consent. Women will undergo endometrial screening on day 10 ±4 following ovulation. Where ovulation testing is inconclusive, screening will be performed on day 23 ±3. An outpatient endometrial biopsy is undertaken following a negative pregnancy test. Additional samples are undertaken as above in those included within the translational sub-study. Samples will be collected by those who do so as part of their job role. Where insufficient samples are obtained the participant has the option for a repeat attempt at collection in the same or next menstrual cycle. Alternatively, they can be referred to the lead site for sample collection. A maximum of two biopsies are be attempted.

Samples will be processed in a central laboratory. A standard protocol for analysis will be followed. A minimum section size of $2mm^2$ should be present. CD138 immunohistochemistry will be undertaken, and a diagnosis of CE will be made in the presence of ≥5 CD138 positive cells per $10mm^2$ of endometrial section based on the existing literature (11). Samples will be reported as positive, or negative, but additional stratification information will be utilised for analysis. Samples will be stratified into 4 categories to further aid prognostic analysis: negative (<5 CD138+/$10mm^2$), mild (5-20 CD138+/$10mm^2$), moderate (21-200 CD138+/$10mm^2$) and severe (>200 CD138+/$10mm^2$). In addition, the pattern of CD138 staining will be recorded and the presence of morphological appearance of plasma cells. Borderline samples will be assessed by two trained observers.

Additional samples

Microbial samples collected as described previously are immediately placed on ice and transferred to a -80°C freezer. Samples are transferred to a specialist laboratory where ribonucleic acid (RNA) extraction will be performed and 16S sequencing conducted. Sequenced data will be passed down a pre-designed data pipeline to allow profiling of the microbiota. These will be analysed in conjunction with trial data and CE status. Additionally, remnant tissue at the lead site will be split between 2mls RNA later and stored at -80°C for RNA related exploratory studies and 4mls 10% dextran coated charcoal treated foetal bovine serum (DCC-FBS) supplemented with DMEM-F12 for culture studies.

Randomisation

Following a screen positive result for CE, women are eligible for randomisation provided they remain eligible based on the previous entry criteria, have not subsequently received a course of antibiotics, or had a delay of longer than three months since the biopsy was undertaken. Consent is further confirmed by a medical doctor, and randomisation undertaken by a member of the site research team using an online web application hosted by the Warwick Clinical Trials Unit. Block randomisation

For peer review only - http://bmjopen.bmj.com/site/about/guidelines.xhtml

is performed in a 1:1 ratio, with Minimisation by age (<35 vs ≥35 years), number of previous miscarriages (≤3 vs >3) and site.

Intervention

Pre-conceptual doxycycline or placebo over-encapsulated for the same appearance will be administered orally at 100mg twice a day for 14-days. Women are advised to commence the intervention on day 1 of the subsequent menstrual cycle. Those entered into the sub-study will undergo ovulation testing within this cycle with repeat biopsy timed as per the screening phase biopsy. Women will be asked to self-record compliance with the intervention.

Safety mechanisms

Doxycycline is contra-indicated in pregnancy as its use has been reported as teratogenic (17, 18). Nevertheless, a review by Cross et al., reported an absence of teratogenic effects and advised the use of doxycycline where clinical circumstances indicate (19). Safety mechanisms have, however, been built into the trial to mitigate the risk of inadvertent use in pregnancy. These include the use of a pregnancy test prior to commencement of doxycycline, commencement on the first day of the menstrual period hence ensuring course completion prior to ovulation and finally women are advised to use barrier contraception throughout the remainder of the cycle. Any pregnancies occurring within 4-weeks of the intervention will be reported and thorough assessment performed to assess for evidence of congenital abnormalities. Emergency unblinding is possible via a 24-hour code breaking telephone line. This will be performed for clinical indications.

Follow-up

Two separate follow-up pathways exist dependent on CE screen status. Women who screen negative will be followed-up to assess subsequent pregnancy outcome at 3-, 6- and 12-months post-biopsy or to the first complete pregnancy outcome, whichever comes first. Women who screen positive will be followed up at 3-, 6-, 12- and 24-months from the point of intervention to the first pregnancy outcome after 24-weeks gestation, 24-months of follow up or the end of the trial, whichever comes first.

End of trial

Given the adaptive nature of the trial the end can be determined by one of several factors. Firstly, following recruitment and completion of 14-months of subsequent follow-up of 1500 women. Secondly, where interim analysis demonstrates either efficacy or futility. Thirdly where mandated by the ethics committee. Fourthly where recruitment falls substantially below target for a period

For peer review only

including but not limited to ≤68 women randomised in the first 6-months. Finally, where trial funding ceases.

Data collection

Pseudo-anonymised data attributed to trial ID will be collected and inputted at sites into a dedicated secure trial database. Warwick Clinical Trials Unit and UHCW (sponsor) act as joint data controllers for this trial.

Data collected at baseline includes date of birth, ethnicity, body mass index, smoking history, medical history, obstetric/pregnancy history, concomitant medication, pregnancy status, a contraception review, and a review of the participant's menstrual cycle. Further information on the timing of the biopsy and any use of antibiotics in the menstrual cycle will be collected at the screening visit. At the randomisation visit, information on concomitant medication, pregnancy status, use of contraception and adverse events will be collected. After the anticipated date of treatment completion, data will be collected on treatment compliance, ongoing contraceptive use, and any adverse events. Follow up will occur at 3-, 6-, 12- and 24-months after randomisation to ascertain pregnancy status. For women who are pregnant, further follow-up will be timed to key pregnancy stages. Pregnancy dating will be confirmed at the booking ultrasound through crown-rump length, viability assessment, concomitant medication review, and any developed pregnancy complications will be noted. Similar information will be captured at ongoing pregnancy reviews. Post-pregnancy, details of the pregnancy outcome, placental histology if available, cytogenetics of products of conception if applicable, pregnancy complications, congenital abnormalities, immediate postpartum maternal and infant infections and maternal infections up to 8-weeks post-delivery, will be collected. All trial participants are free to withdraw from the trial at any time point. Unless a participant explicitly withdraws consent, they and their infant will be followed up where possible. On withdrawal, no further data will be collected and, if requested, all participant data will be removed.

Sample size

The total number of women entered for screening will be determined by the CE positivity ratio on screening. A 50% positivity rate has been assumed with a total screen population of 3062 accounting for participant drop out of 2%. This will give a maximum sample size of 1500 randomised with 750 in each arm. The study is therefore powered to detect an 8% between group difference this has been pragmatically selected due to limited previous high-quality work in the area. There are three planned interim analyses after 100, 300 and 900 women have reached 28 weeks post-

For peer review only - http://bmjopen.bmj.com/site/about/guidelines.xhtml

randomisation. These analyses will assess for futility and be reviewed by the DMC. Prespecified stopping rules as documented in the statistical analysis plan will be applied.

Data analysis

Data will be analysed by intention to treat at randomisation. The treatment difference for the primary outcome will be assessed by deriving the posterior distribution of a Bayesian logistic regression model with a weakly informative prior distribution for all parameters. Although unlikely, in the event of missing data for primary outcomes of >10% imputation techniques will be used. The result for the primary outcome will be reported as the median value of the simulated odds ratios with the corresponding credible intervals. Secondary outcomes as previously outlined will be similarly assessed. The time to event (first pregnancy) analysis will be assessed using semiparametric proportional hazard regression. The following pre-defined subgroup analyses of the primary outcomes will also be assessed by age (<35 vs ≥35), number of previous miscarriages (≤3 vs >3), severity of CD138 expression and pattern of CD138 expression.

Adverse event management

Adverse events (AE) will be collected to 30 days post-trial intervention, this may be either screening biopsy, treatment completion or secondary biopsy whichever comes later dependent on the participants trial arm. Adverse events captured elsewhere in the trial data do not require reporting, including those related to pregnancy outcome. In addition, common pregnancy related serious adverse events as seen in box 1 are exempt from SAE reporting but should be reported as adverse events. A causality assessment will be performed by a doctor for all SAEs and AEs. These will be assessed by the trial management group and the DMC. SAEs and AEs thought to be related or unexpected will be reported to the Medicines and Healthcare Products Regulatory Agency) and Research Ethics Committee, the sponsor and the chairs of the Trial Steering Committee and DMC.

**Box 1.** Adverse events exempt from SAE reporting but recorded as AEs
**Admission to hospital for nausea and vomiting**
**Admission to hospital for headaches**
**Admission to hospital for raised blood pressure or pregnancy induced hypertension**
**Admission to hospital for vaginal bleeding**

Monitoring

All clinicians involved in consent should have up to date Good Clinical Practice training. Samples may be collected by those who fulfil this role in a clinical capacity. All involved will undergo a relevant

For peer review only - http://bmjopen.bmj.com/site/about/guidelines.xhtml

programme of training. Site visits will be conducted where concerns arise. Data will be monitored for completeness and quality, where queries arise these will be escalated to the site involved.

A Trial Management Group (TMG) comprising co-investigators, allied experts, a patient public involvement advisor and the trial management staff will meet monthly to bimonthly to review the day-to-day running of the trial.

A Trial Steering Committee (TSC) has been established comprising a group of independent experts covering all trial areas and lay members. The TSC will be responsible for protocol approval and review of any protocol changes, advising on aspects of trial conduct, monitoring trial progress and consideration of any recommendations from the DMC. They will meet not less than once a year. Any protocol modifications as agreed by the TSC will be submitted to relevant parties.

A DMC has been established comprising independent experts. They will ensure monitoring of outcomes and safety aspects during the trial.

An authorised representative of the Sponsor has approved the final version of the protocol with respect to the trial design, conduct, data analysis and interpretation and plans for publication and dissemination of results.

Integrated trial design

This trial utilises an integrated adaptive approach. This allows facilitation of both assessing the core clinical outcomes but also the development of a concurrent sample and dataset for mechanistic evaluation should an effect be seen. It also allows better understanding of the aetiology of the condition including through the follow-up of those with a negative result. This integrated mechanistic clinical approach forms a model for a more economical approach to trial design in poorly understood conditions. Through more efficient use of resources, it allows the development of a holistic approach to disorder determination. A key hallmark of this design is the flexibility provided by the adaptive design and integration of sample banking. This ensures that a rigorous methodological approach is taken whilst still facilitating exploratory research. In fields such as this, which face the difficult challenges of a limited evidence base and disorder heterogeneity such approaches are likely to prove increasingly necessary.

Patient and public involvement

The trial has been designed with the involvement of the Lily Mae Foundation, a charity supporting women who have lost a baby through miscarriage, stillbirth, neonatal death or medical termination.

For peer review only - http://bmjopen.bmj.com/site/about/guidelines.xhtml

Amy Jackson, co-founder of the charity, sits on the trial management group. She has additionally led on the development of patient-facing materials.

Alterations to study design since commencement

Initial establishment of the trial was on the basis of co-primary outcomes: ongoing pregnancy at 12 weeks at the end of the trial and livebirth ≥24 + 0 weeks gestation at the end of the trial, in the first or subsequent pregnancy. Like other clinical trials, the timeframe for recruitment has meant the trial has been heavily impacted by the COVID-19 pandemic. This has resulted in under-recruitment, with a lower-than-expected trial enrolment size falling short of the targeted 3,062 screened and 1500 randomised. In view of this, the Trial Steering Committee unanimously agreed on 22nd November 2022 that, given a likely smaller than anticipated participant population, reduction to a single primary outcome of livebirth ≥24 + 0 weeks gestation at the end of the trial, in the first or subsequent pregnancy, was necessary to ensure statistical robustness. A formal protocol and REC amendment was made, and the change was approved by both the TSC and chair of the DMC.

Also because of the COVID-19 pandemic, a decision was made to allow consent to randomisation to be undertaken over the telephone (this previously required written consent). This was enacted to reduce hospital site visits and a REC amendment made.

ETHICS AND DISSEMINATION

The trial will be conducted in full conformance with the principles of the Declaration of Helsinki, ICH Good Clinical Practice (GCP) guidelines and the UK Statutory Instrument Number 1031 that implements the Medicine for Human Use (Clinical Trials) Directive 2004 and subsequent amendments. Ethical approval for the trial has been obtained from the NHS Research Ethics Committee (REC) Northwest- Haydock (REC reference: 19/NW/0462, REC approval date: 20/08/2019). The key ethical consideration of the trial revolves around the paradox of asking women who are eager to achieve successful pregnancy to avoid pregnancy for three months whilst undergoing trial procedures. To mitigate this, trial procedures will be kept to as short a period as possible. It is hoped the potential for gain minimises this paradox. All participants will provide written informed consent including for the biobanking of samples at the point of the screening biopsy. Telephone consent will subsequently be taken at the point of randomisation. The consent form is included within the supplementary material.

The trial will be reported in accordance with the Consolidated Standards of Reporting Trials (CONSORT) guidelines (20). The trial will be published in an open-access peer reviewed journal with authorship agreed as per ICMJE requirements (21). The full protocol and trial findings will also be

For peer review only - http://bmjopen.bmj.com/site/about/guidelines.xhtml

reported in the NIHR journals library. In addition, dissemination at national and international specialist conferences will be undertaken. Exploratory outcomes will be disseminated separately in open-access peer reviewed journals to accommodate their more specialist nature.

Trial progress to date

This trial opened for recruitment on 19 December 2019 and closed to new registrations on 30 September 2022. The trial is currently in the follow-up period, and the study is expected to be completed by 30 April 2024.

For peer review only

Contributors: SQ, JF, & MU were applicants on the original grant application, SQ is the chief investigator. JO wrote first draft of this paper, NB, GB, and JG commented on successive drafts of the paper. All authors read and approved the manuscript.

Funding: This project is funded by the Efficacy and Mechanism Evaluation (EME) Programme, an MRC and NIHR partnership (Funder reference: 17/60/22). The views expressed in this publication are those of the authors and do not necessarily reflect those of the MRC, NIHR or Department of Health and Social Care.

Competing interests: None declared.

Patient and public involvement: Patients and/or the public were involved in the design, or conduct, or reporting, or dissemination plans for this research. These involvements are detailed in the Methods and analysis section.

Data availability statement: Following completion of the study, all data requests should be submitted to the Chief Investigator via WCTUDataAccess@Warwick.ac.uk for consideration. Access to anonymised data may be granted following review, and subject to a signed data sharing agreement.

Additional study details:

Trial sponsor

University Hospitals Coventry and Warwickshire NHS Trust (Sponsor reference: SQ411218). Sponsor contact: Ceri Jones (ceri.jones@uhcw.nhs.uk).

Roles and responsibilities

Trial Steering committee (TSC): Role – Approval protocol, advise on aspects of trial conduct, monitor trial progress, consider recommendations from DMC and if necessary agreeing changes to the protocol. TSC members - Andrew Horne (Chair), Jim Thornton (Clinical Expert), Sally Kerry (Medical Statistician), Rachel Roberts (Public Member), Ceri Jones (Sponsor observer), George Bouliotis (Medical Statistician), Siobhan Quenby (Chief Investigator).

Data Monitoring Committee (DMC): Role – Ensure monitoring of outcomes and safety aspects during the trial. Members - Alex McConnachie (Chair/Statistician), Sharon Cameron (Clinical expert member), Mary Stephenson (Clinical expert member), George Bouliotis (Trial statistician). The DMC is an independent committee from the trial sponsor.

Supplementary material:

For peer review only - http://bmjopen.bmj.com/site/about/guidelines.xhtml

1) **Participant information leaflet**
2) **Participant consent form**
3) **Participant instruction leaflet**

For peer review only

For peer review only - http://bmjopen.bmj.com/site/about/guidelines.xhtml

References

1. Quenby S, Gallos ID, Dhillon-Smith RK, Podesek M, Stephenson MD, Fisher J, et al. Miscarriage matters: the epidemiological, physical, psychological, and economic costs of early pregnancy loss. The Lancet. 2021 2021-05-01;397(10285):1658-67.

2. Prior M, Bagness C, Brewin J, Coomarasamy A, Easthope L, Hepworth-Jones B, et al. Priorities for research in miscarriage: a priority setting partnership between people affected by miscarriage and professionals following the James Lind Alliance methodology. BMJ Open. 2017 Aug 23;7(8):e016571.

3. Department of Health & Social Care. Policy Paper: Women's Health Strategy for England. In: Department of Health & Social Care, editor. 2022.

4. Odendaal J, Quenby S, Sammaritano L, Macklon N, Branch DW, Rosenwaks Z. Immunologic and rheumatologic causes and treatment of recurrent pregnancy loss: what is the evidence? Fertil Steril. 2019 Dec;112(6):1002-12.

5. McQueen DB, Perfetto CO, Hazard FK, Lathi RB. Pregnancy outcomes in women with chronic endometritis and recurrent pregnancy loss. Fertil Steril. 2015 Oct;104(4):927-31.

6. Moreno I, Codoner FM, Vilella F, Valbuena D, Martinez-Blanch JF, Jimenez-Almazan J, et al. Evidence that the endometrial microbiota has an effect on implantation success or failure. Am J Obstet Gynecol. 2016 Dec;215(6):684-703.

7. Moreno I, Cicinelli E, Garcia-Grau I, Gonzalez-Monfort M, Bau D, Vilella F, et al. The diagnosis of chronic endometritis in infertile asymptomatic women: a comparative study of histology, microbial cultures, hysteroscopy, and molecular microbiology. American Journal of Obstetrics and Gynecology. 2018 2018/06/01/;218(6):602.e1-.e16.

8. Cicinelli E, De Ziegler D, Nicoletti R, Colafiglio G, Saliani N, Resta L, et al. Chronic endometritis: correlation among hysteroscopic, histologic, and bacteriologic findings in a prospective trial with 2190 consecutive office hysteroscopies. Fertil Steril. 2008 Mar;89(3):677-84.

9. Huang W, Liu B, He Y, Xie Y, Liang T, Bi Y, et al. Variation of diagnostic criteria in women with chronic endometritis and its effect on reproductive outcomes: A systematic review and meta-analysis. J Reprod Immunol. 2020 Aug;140:103146.

10. Puente E, Alonso L, Laganà AS, Ghezzi F, Casarin J, Carugno J. Chronic Endometritis: Old Problem, Novel Insights and Future Challenges. Int J Fertil Steril. 2020 Jan;13(4):250-6.

11. Liu Y, Chen X, Huang J, Wang CC, Yu MY, Laird S, et al. Comparison of the prevalence of chronic endometritis as determined by means of different diagnostic methods in women with and without reproductive failure. Fertil Steril. 2018 May;109(5):832-9.

For peer review only - http://bmjopen.bmj.com/site/about/guidelines.xhtml

12. McQueen DB, Bernardi LA, Stephenson MD. Chronic endometritis in women with recurrent early pregnancy loss and/or fetal demise. Fertil Steril. 2014 Apr;101(4):1026-30.

13. Cicinelli E, Matteo M, Tinelli R, Pinto V, Marinaccio M, Indraccolo U, et al. Chronic endometritis due to common bacteria is prevalent in women with recurrent miscarriage as confirmed by improved pregnancy outcome after antibiotic treatment. Reprod Sci. 2014 May;21(5):640-7.

14. Kitaya K, Matsubayashi H, Takaya Y, Nishiyama R, Yamaguchi K, Takeuchi T, et al. Live birth rate following oral antibiotic treatment for chronic endometritis in infertile women with repeated implantation failure. Am J Reprod Immunol. 2017 Nov;78(5).

15. Clain E, Devine K. Endometrial receptivity, to test or not to test: the evidence on contemporary assays. F&S Reviews. 2023 2023/01/01/;4(1):50-65.

16. Chan AW, Tetzlaff JM, Altman DG, Laupacis A, Gøtzsche PC, Krleža-Jerić K, et al. SPIRIT 2013 statement: defining standard protocol items for clinical trials. Ann Intern Med. 2013 Feb 5;158(3):200-7.

17. Muanda FT, Sheehy O, Bérard A. Use of antibiotics during pregnancy and the risk of major congenital malformations: a population based cohort study. British Journal of Clinical Pharmacology. 2017 2017/11/01;83(11):2557-71.

18. Abd-Allah ER, Abd El-Rahman HA. Influence of doxycycline administration on rat embryonic development during organogenesis. Journal of Biochemical and Molecular Toxicology. 2021 2021/01/01;35(1):e22613.

19. Cross R, Ling C, Day NP, McGready R, Paris DH. Revisiting doxycycline in pregnancy and early childhood--time to rebuild its reputation? Expert Opin Drug Saf. 2016;15(3):367-82.

20. Schulz KF, Altman DG, Moher D, the CG. CONSORT 2010 Statement: updated guidelines for reporting parallel group randomised trials. BMC Medicine. 2010 2010/03/24;8(1):18.

21. Editors ICoMJ. Defining the Role of Authors and Contributors 2023 [cited 2023 24 August]. Available from: https://www.icmje.org/recommendations/browse/roles-and-responsibilities/defining-the-role-of-authors-and-contributors.html.

For peer review only

Figure title

Figure 1. Study participant flowchart

For peer review only

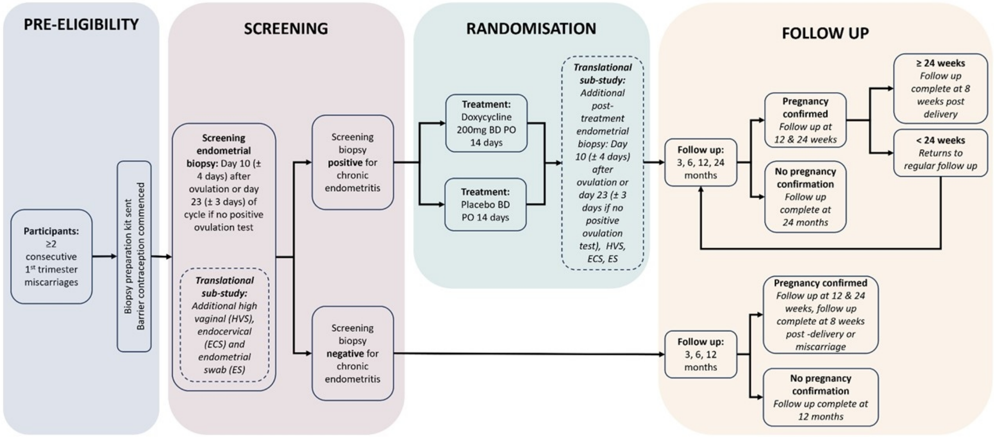

Figure 1: Study participant flowsheet.

338x190mm (96 x 96 DPI)

CERM Chronic Endometritis and Recurrent Miscarriage

[Print on Trust Headed Paper]

Crystal Mark 23135 Clarity approved by Plain English Campaign

Participant information sheet (CERM A)

Information for women taking part in the chronic endometritis and recurrent miscarriage (CERM) trial

Trial title

Chronic endometritis and recurrent miscarriage – the CERM trial

This information sheet is available in **large print** from the university trial team (email: cerm@warwick.ac.uk).

Introduction

You are invited to take part in a research trial. Please read this information sheet carefully before deciding whether to take part. It explains why this research trial is being done and what it means for you if you decide to take part. You will have the opportunity to talk to a member of the hospital research team about this trial and they will be happy to answer any questions you have. You can also discuss the trial with your GP or the obstetrician or gynaecologist caring for you.

This leaflet is divided into two parts. **Part 1** tells you the purpose of the trial and what will happen to you if you take part. **Part 2** gives you more detailed information about how the trial will be carried out.

Part 1

What is the purpose of the research trial?

The aim of this research trial is to find out if antibiotics can reduce miscarriage. In some women the lining of the womb (the endometrium) is inflamed. Researchers have found a link between this and miscarriage. A healthy endometrium is important for the embryo to be able to attach to the womb. It is thought that endometritis disrupts this process, and can lead to a miscarriage. Treating endometritis with antibiotics may reduce the inflammation and the likelihood of a miscarriage. This has not been tested. This research trial will test this theory by comparing a 14-day course of an antibiotic (doxycycline) against a placebo (a 'dummy treatment' which will look exactly the same as the antibiotic but contains no active ingredients) to find out if taking antibiotics reduces miscarriages. The trial will be 'double blind'. This means that the women and the trial researchers will not know who is taking the antibiotic capsules and who is taking the placebo. It will also be a randomised controlled trial. This means, if you decide to take part, which capsules you receive (the antibiotic or the placebo) will be decided by chance. The trial will take place in NHS hospitals in the United Kingdom and will involve over 3,000 women who have recurrent miscarriage.

> Inflammation of the lining of the womb is a condition called **endometritis.**

> **Recurrent miscarriage** means two or more miscarriages in a row.

For peer review only - http://bmjopen.bmj.com/site/about/guidelines.xhtml

CERM Chronic Endometritis and Recurrent Miscarriage

[Print on Trust Headed Paper]

Not everyone who has recurrent miscarriage has endometritis, so before you can take part in the randomised controlled trial the hospital trial team will need to take an endometrial biopsy (a small sample of tissue from your endometrium) and examine it under a microscope to find out if you do. We (the university research team) estimate that half the women tested will have endometritis and so be able to take part in the randomised controlled trial.

Researchers also suspect that inflammation of the endometrium may be caused by an imbalance of the microbiome that lives in the reproductive tract (the vagina, cervix, womb, fallopian tubes and ovaries). Another part of the trial is to look at the endometrium and microbiome to see how antibiotics affect these. A diagram of this research trial is shown in figure 1.

The **microbiome** is the name given to all the microbes that live in and on our bodies. It is mostly made up of bacteria, and everyone's microbiome is unique.

For peer review only

For peer review only - http://bmjopen.bmj.com/site/about/guidelines.xhtml

CERM Chronic Endometritis and Recurrent Miscarriage

[Print on Trust Headed Paper]

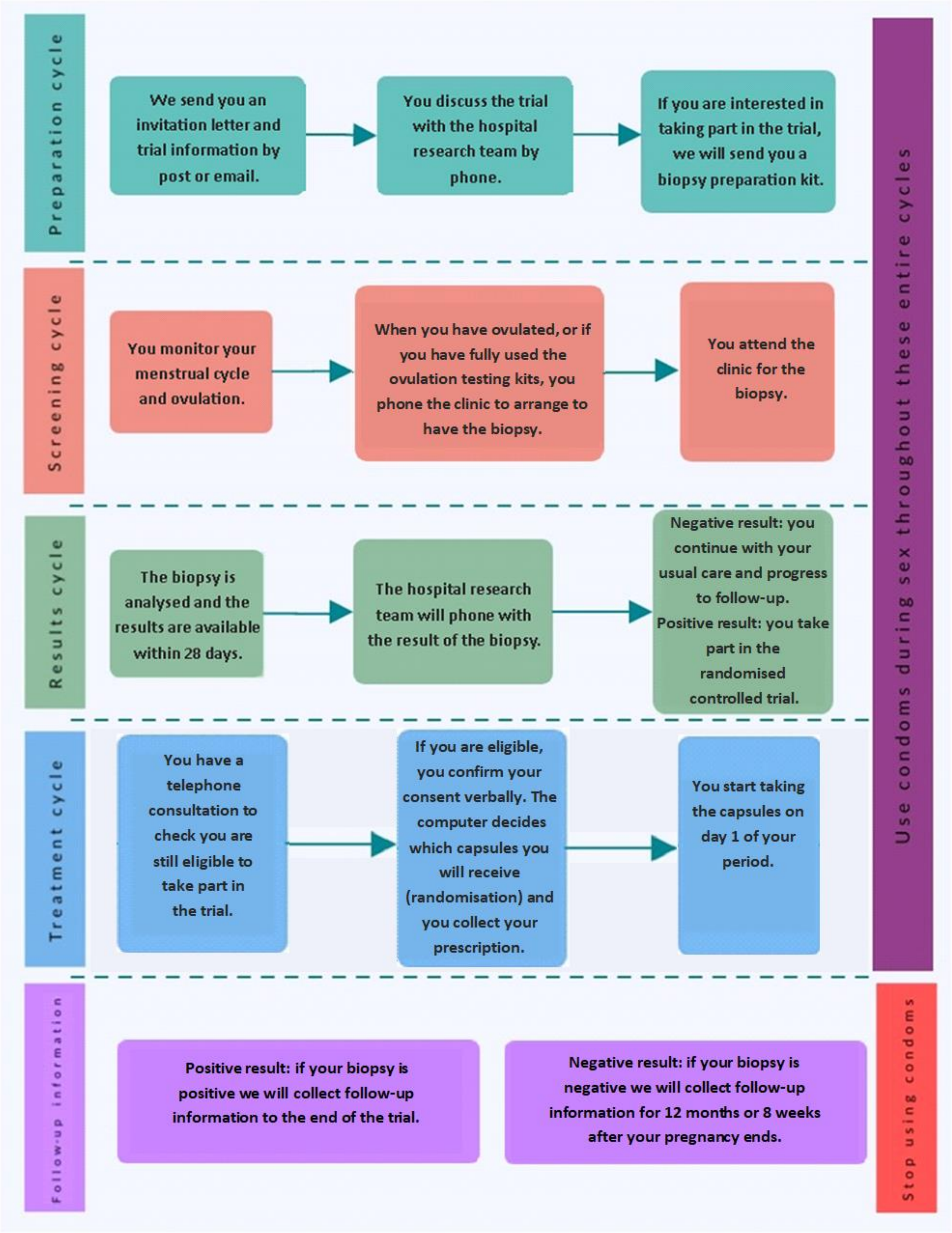

Figure 1 – an overview of the CERM trial

For peer review only - http://bmjopen.bmj.com/site/about/guidelines.xhtml

[Print on Trust Headed Paper]

Screening trial

Why have I been invited?

You have been invited to take part in this research trial because you have had two or more miscarriages (recurrent miscarriage).

Do I have to take part?

Taking part in the research trial is entirely voluntary. It is up to you whether or not you take part. You do not have to take part, and there will be no difference in the care you receive if you choose not to take part. If you want to take part, you will be asked to sign a consent form to confirm you have agreed to take part. You will keep a signed copy of the form. Even after signing the consent form, you can withdraw from the trial at any time if you change your mind, without having to give a reason. This will not affect the care you receive.

This information sheet will now explain what will happen if you decide to take part in this trial.

What will happen next?

A member of the hospital research team will talk to you over the phone (or speak to you face-to-face if you prefer this and if it is possible) to explain what the trial is about and what taking part would mean for you. You can ask the hospital research team any questions you have.

What will happen if I agree to take part?

1. **We will check to see if you are eligible –** You must be aged 18 to 41 years old. The hospital research team will ask about your obstetric and medical history to see if you are eligible to take part in the research trial. If you have not had any investigations to find out the cause of your recurrent miscarriage, these will be done when you attend the clinic. If a cause for your miscarriages is found, you will not be eligible for the trial. An important part of the eligibility check is to find out if you are willing to use condoms whenever you have sex throughout the entire menstrual cycles when you are:
    - preparing for your biopsy;
    - waiting for the result of your biopsy; and
    - taking the capsules.

   Doxycycline, the antibiotic used in the study, can be passed on in breast milk. The effects this could have on babies are not known. For this reason, and to make sure the study is absolutely safe, you will not be able to join the trial if you are breastfeeding.

2. **We will ask for permission to send you a 'biopsy preparation kit' –** If you are eligible to have a biopsy taken, the hospital research team will ask for your verbal consent to post a biopsy preparation kit to you. If you agree, this will be recorded in your hospital case notes and you will be given a trial screening number. The kit contains instructions, a period tracker, condoms and an ovulation testing kit. If you are prescribed a course of antibiotics between giving verbal consent and receiving the biopsy preparation kit, or

For peer review only - http://bmjopen.bmj.com/site/about/guidelines.xhtml

[Print on Trust Headed Paper]

while you are using the biopsy preparation kit, please contact a member of the hospital research team (see the section: How can I contact the hospital trial team?). The biopsy may need to be delayed and the hospital team may need to send you another biopsy preparation kit once you have finished your course of antibiotics.

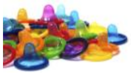

3. **Preparing for the biopsy –** It is very important that you **do not** try to conceive and that you use condoms whenever you have sex throughout this entire menstrual cycle. Condoms are provided in the kit and you can get more by contacting the hospital research team or family-planning services.

   Starting on the first day of your next menstrual cycle after receiving the kit, you should use the period tracker to record:
   - the days of your period; and
   - the results of the ovulation testing.

   The biopsy needs to be taken a few days after you have ovulated. Ovulation usually happens around day 12 to 16, depending on the length of your cycle. We provide a period tracker and ovulation testing kit to help you work out the best time to have the biopsy taken. When the ovulation test shows you have ovulated, or if you have used all of the kit and it does not show you have ovulated, you will need to contact the hospital research team to arrange an appointment to come in for your biopsy.

4. **Having the biopsy –** Please bring your period tracker with you when you come to the clinic for your biopsy. In the clinic a doctor will explain what will happen and check you are eligible to take part in the trial. You will have the opportunity to ask them any questions you might have. You are welcome to bring your partner, a family member, or a friend to this appointment, if the local rules on visitors to the clinic allow this. If you are eligible and would like to take part, you will be asked to sign a consent form. You will also be asked to take a pregnancy test before the biopsy. A healthcare professional will ask you for a sample of urine and they will test it. If the test shows you are pregnant, the biopsy will be cancelled because it could put the pregnancy at risk, and you will be referred to your GP who will arrange for your care. If the pregnancy test shows you are not pregnant, you will register for the trial and be given a trial identification (ID) number. A doctor will then explain the endometrial biopsy procedure.

   Before the procedure you will have a vaginal examination to find out the position of your womb so the healthcare professional can take the biopsy. The biopsy usually takes a couple of minutes. The healthcare professional will take the biopsy by passing a thin plastic tube through your cervix and into your womb. We will take a small sample of the lining of your womb. We will send part of this sample to the laboratory at the University Hospitals Coventry and Warwickshire NHS Trust (UHCW) to be analysed under the microscope to find out whether or not you have endometritis. With your permission, any tissue that is left over following analysis, and clinical data that does not identify you will be stored in Tommy's National Reproductive Health Biobank and used for future ethically approved research. This will not affect the sample sent to UHCW for analysis. The sample

For peer review only

For peer review only - http://bmjopen.bmj.com/site/about/guidelines.xhtml

CERM Chronic Endometritis and Recurrent Miscarriage

[Print on Trust Headed Paper]

sent to UHCW for analysis will be labelled with your trial ID number and initials, not your name.

The healthcare professional will do all they can to make sure they collect enough endometrial tissue to be analysed, but in a few cases this may not be possible. If the healthcare professional is not able to collect a sample or cannot collect enough tissue to be analysed, depending on the reason for this, the health professional carrying out the biopsy will discuss the following options with you.

- The same healthcare professional will repeat the biopsy during the same appointment
- A different healthcare professional will take the biopsy during the same appointment
- You come back to the clinic for another biopsy during your next menstrual cycle
- You have a biopsy taken at UHCW if you are happy and willing to travel to this site
- You choose not to repeat the biopsy and withdraw from the trial

After the biopsy you will be given a pregnancy test kit to take home.

5. **You get your biopsy results and the next steps** – While you are waiting for your biopsy results, it is important that you continue to use condoms whenever you have sex. We will phone you when your biopsy results are available. This will usually be within four weeks.

   If your results show that you do not have endometritis, you will continue with your usual care.
   - We will phone you at three, six and 12 months to see if you are still trying to get pregnant, if you are pregnant or if you have had any miscarriages or terminations. If you get pregnant between our phone calls, please let the hospital research team know (see the section: How can I contact the hospital trial team?).
   - If you get pregnant, we will collect follow-up information from the scans you have during your pregnancy.
   - If you have a baby, we will phone you eight weeks after the birth to ask about your health and your baby's health. The details we ask for will include the date of the birth, how many weeks pregnant you were at the time of the birth, how your baby was delivered, and your baby's weight and sex.
   - We will ask for details of any complications and abnormalities (for both you and your baby).

If your results show that you have endometritis, you may be eligible to take part in the randomised controlled trial. The following section explains what will happen during this stage. It is very important that you use condoms whenever you have sex during this entire menstrual cycle, and not just when you are taking the capsules.

For peer review only - http://bmjopen.bmj.com/site/about/guidelines.xhtml

CERM
Chronic Endometritis and Recurrent Miscarriage

[Print on Trust Headed Paper]

Randomised controlled trial

A member of the hospital research team will phone you to explain that your results show you have endometritis and you may be eligible to take part in the randomised controlled trial. They will explain what this part of the trial is about and what taking part would mean for you. You can ask the hospital research team any questions you have.

What will happen if I agree to take part?

1. **We will check to see if you are still eligible.** We will make an appointment for you to have a consultation with a member of the hospital research team. The consultation may take place face-to-face or by phone or video call. You can ask any questions you have and you can ask your partner, a family member or a friend to join the consultation.

   During your consultation a doctor will ask you about your medical and obstetric history to check you are eligible to take part in the randomised controlled trial. If investigations have revealed a treatable cause for your recurrent miscarriage since you had the endometrial biopsy, you will not be eligible for the trial and a doctor will discuss your treatment options with you.

   An important part of the eligibility check is to find out if you are still willing to use condoms whenever you have sex throughout your entire menstrual cycle when you are taking the capsules.

   You will be asked to do a pregnancy test (using the pregnancy test kit provided at your biopsy visit) **and** call the hospital research team with the result. If the test shows you are pregnant, you will not be eligible for the trial and you will be referred to your GP who will arrange your care.

   Following the eligibility check, the doctor will ask you to confirm that you are happy to take part in the randomised controlled trial. You are free to withdraw from the trial at this stage or at any point, for any reason.

2. **Randomisation –** Whether you will receive doxycycline or the placebo is decided by chance. You and the trial researchers will not know who is taking the doxycycline and who is taking the placebo, but it will be possible to find this out if it becomes necessary for your clinical care.

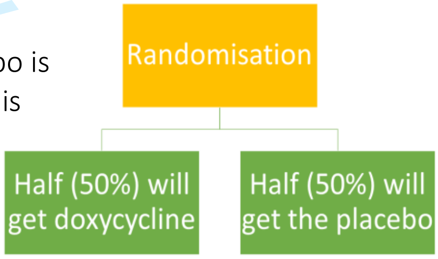

3. **Collecting your prescription and taking the capsules –** You can collect the capsules from a member of the hospital research team or from the hospital pharmacy (after you have collected your prescription from the hospital research team) at an agreed time that is convenient to you.

For peer review only - http://bmjopen.bmj.com/site/about/guidelines.xhtml

CERM Chronic Endometritis and Recurrent Miscarriage

[Print on Trust Headed Paper]

You will be given an information booklet with the capsules. The booklet gives full instructions on how to take the capsules and you should read it carefully. It has guidance on what to do if something happens during the trial, and what precautions you should take while you are taking the capsules.

You can start taking the capsules on the first day of your next menstrual cycle. The dose is one capsule twice a day, 12 hours apart, for 14 days. Please fill in the treatment diary and mark a cross in the diary every time you take a capsule.

These capsules contain gelatine and small amounts of lactose.

4. You will be given a CERM trial participant card. It is very important that you carry your trial participant card with you at all times while you are taking the capsules, and show it to anyone you are receiving healthcare from. Throughout this entire menstrual cycle, it is important that you use condoms whenever you have sex.

5. If you miss your next period or suspect you are pregnant - Take a home pregnancy test. Follow the instructions that come with the test. Contact the hospital research team if the pregnancy test is positive or if you are not sure of the result.

Follow-up information

We will only collect information about you and your baby that is relevant to taking part in the CERM research trial. We will ask you for this information, but if you are not sure of, or don't know, the details we need we will refer to your hospital records (at whichever hospital you receive care from during the trial) or GP records (or both).

- We will phone you after you have taken all of your capsules to see if you have taken all of them as prescribed and if you have had any side effects. Please refer to your treatment diary for this information.
- We will phone you at three, six, 12 and 24 months plus at the end of the trial to see if you are still trying to get pregnant, if you are pregnant or if you have had any miscarriages or terminations. If you get pregnant between our phone calls, please let the hospital research team know (see the section: How can I contact the hospital trial team?).
- If you get pregnant, we will collect follow-up information from the scans you have during your pregnancy.
- If you have a baby, we will phone you eight weeks after the birth to ask about your health and your baby's health. The details we ask for will include the date of the birth, how many weeks pregnant you were at the time of the birth, how your baby was delivered, and your baby's weight and sex.
- We will ask for details of any complications, abnormalities, infections, investigations and results, treatment and hospital stays (for both you and your baby).

Expenses and payments

For peer review only - http://bmjopen.bmj.com/site/about/guidelines.xhtml

[Print on Trust Headed Paper]

There are no payments for taking part in this research trial. We will refund any hospital parking charges you have to pay for hospital appointments that relate to this research.

What are the clinical alternatives?

Currently there are no clinical alternatives, as few treatments have been shown to prevent miscarriage.

What are the possible disadvantages and risks of taking part?

You and your partner will need to delay trying to conceive because it is important that you use condoms whenever you have sex throughout your entire menstrual cycles when you are:

- preparing for your biopsy;
- waiting for the result of your biopsy; and
- taking the capsules.

We have streamlined the trial processes to make sure the time you need to delay trying to conceive is as short as possible.

Some women find the endometrial biopsy is painful and may get cramping at the time of the biopsy. Taking paracetamol and ibuprofen an hour before the biopsy can help with this. If you need it, gas and air (Entonox) will be available while you are having the biopsy. Some women may have some vaginal bleeding (spotting) after the biopsy is taken. This will stop quickly on its own.

Please see the 'Taking my capsules on the chronic endometritis and recurrent miscarriage (CERM) trial' leaflet for details of any possible side effects while you are taking the capsules, and what to do if something happens when you are taking them.

What are the possible benefits of taking part?

We do not know if taking part in the research trial would benefit you personally. Taking part will show whether you have chronic endometritis. The results will help us provide advice on treatment options for women who have recurrent miscarriage.

What happens when the research trial stops?

The research trial is planned to take up to 48 months to complete.

Whenever the research trial stops, the university trial team will analyse the information collected to decide if antibiotics should be prescribed to women with chronic endometritis. In the future, these results will help women who are experiencing recurrent miscarriage.

This is the end of Part 1.

If you are interested in taking part in the trial, please read the important information in Part 2 before making your decision.

For peer review only - http://bmjopen.bmj.com/site/about/guidelines.xhtml

[Print on Trust Headed Paper]

Part 2

What if there is a problem?

If you have any concerns about any aspect of this study, you should ask to speak to the researchers who will do their best to answer your questions. If you are still not happy and want to make a complaint, you can do this through the NHS complaints procedure.

Please write to:

Complaints Manager
University Hospitals of Coventry and Warwickshire NHS Trust
Clifford Bridge Road
CV2 2DX

Or phone: 02476 965 198

In the unlikely event that you are harmed by taking part in this study, compensation may be available. If you suspect that the harm is the result of someone's negligence, you may be able to take legal action, but you may have to pay any costs involved and you should get legal advice about this.

For independent advice on research, you can contact PALS (Patient Advice and Liaison Service) on Freephone 0800 028 4203, or you can email them at feedback@uhcw.nhs.uk.

What Covid-19 precautions should I take when coming to hospital for the trial?

Your local NHS trust may have introduced measures to reduce your risk of being exposed to Covid-19. Please see the relevant hospital's website or ask the hospital trial team for more information.

Should I have the Covid-19 vaccine while I am taking part in the trial?

The CERM trial team, which includes clinicians and a pharmacist, have confirmed that there are no interactions between the drug used in the trial (doxycycline) and available Covid-19 vaccines. This means there are no safety concerns with you having a Covid-19 vaccine if you are offered one while you are taking part in the trial.

The hospital research team will ask you to let them know if you do have a Covid-19 vaccine so they can record the details alongside any other medications you are taking. If you have any questions, please get in touch with the hospital research team or your GP.

How will my information be used?

University Hospital Coventry and Warwickshire (UHCW) is the sponsor for this trial in the United Kingdom. The trial will be managed by Warwick Clinical Trials Unit at the University of Warwick (UoW). UHCW and the UoW will use information you provide and information from your hospital records and your GP records to carry out this trial, and will act as joint data

For peer review only - http://bmjopen.bmj.com/site/about/guidelines.xhtml

CERM Chronic Endometritis and Recurrent Miscarriage

[Print on Trust Headed Paper]

controllers for the trial. This means that, together, they are responsible for looking after your information and using it properly.

We will only collect information about you and your baby that is relevant to taking part in the CERM research trial. We will ask you for this information, but if you are not sure of, or don't know, the details we need we will refer to your hospital records (at whichever hospital you receive care from during the trial) or GP records (or both). The information we will collect is listed below.

- The name of any investigations you have had
- The results of any investigations you have had
- The name of any illnesses, conditions or dependencies you have or have had
- The name of any prescription medications you are taking, the dose or units, and how long you have been taking them
- Details of any pregnancies, miscarriages or terminations
- The results of any pregnancy scans
- Details of any births, including the date, how many weeks pregnant you were at the time of the birth, how your baby was delivered, whether your baby was born alive or was stillborn, and your baby's weight and sex
- Details of any complications, abnormalities, infections, investigations and results, treatment and hospital stays (for both you and your baby)
- The results from the routine genetic analysis of any miscarriage tissue and tissue from the placenta (we will only collect this from you if you choose to take part in the randomised controlled trial)

Your hospital will collect information from you and your medical records and will pass this to us for this research trial in line with our instructions. This information will include your initials and date of birth. We would also like to collect details of your ethnic background and race, but you can choose not to provide this information if you prefer. We will ask for your permission to tell your GP that you are taking part in the research trial. If you do not want us to tell your GP, you will not be able to take part.

Your hospital will keep your name, NHS number and contact details confidential and will not pass this information to UHCW or the UoW. Your hospital will use this information, as needed, to contact you about the research trial, to make sure that relevant information about the trial is recorded for your care, and to oversee the quality of the trial. Certain people from UHCW, the UoW and regulatory organisations may look at your medical and research records to check that the research trial is accurate. UHCW and the UoW will only receive information that does not contain any details that directly identify you. The people who analyse the information will not be able to identify you and will not be able to find out your name, NHS number or contact details. UHCW and the UoW will keep identifiable information about you for 10 years after the trial has finished (if the results of your biopsy show that you **do not** have endometritis) or for 25 years after the trial has finished (if the results of your biopsy show that you **do** have endometritis).

For peer review only - http://bmjopen.bmj.com/site/about/guidelines.xhtml

CERM Chronic Endometritis and Recurrent Miscarriage

[Print on Trust Headed Paper]

When you agree to take part in a research trial, the information about your health and care may be provided to researchers running other research studies in this organisation and in other organisations. These organisations may be universities, NHS organisations or companies involved in health and care research in this country or abroad. Organisations and researchers will only use your information to carry out research in line with the UK Policy Framework for Health and Social Care Research. This information will not identify you and will not be combined with other information in a way that could identify you. The information will only be used for the purpose of health and care research. It will not affect your care, and organisations and researchers cannot use it to contact you. Your information will not be used to make decisions about future services that are available to you, such as insurance.

Your rights to see, change or move your information are limited, as your information is managed in specific ways to make sure the research is reliable and accurate. If you withdraw from the trial, the information that has already been collected about you will be kept. To protect your rights, as few details as possible that could identify you will be collected.

To find out more about how your information is handled, you can visit the privacy notices of the data controllers.
www.uhcw.nhs.uk/privacy/
www.warwick.ac.uk/services/idc/dataprotection/privacynotices/researchprivacynotice

Will my information be kept confidential?

Yes. All information collected about you is strictly confidential. Any research information UHCW and the UoW collect will refer to you only by a unique trial ID number and your initials, so the risk of you being identified is very low. All your information will be stored securely and held at the Warwick Clinical Trials Unit, in line with all relevant UK laws.

What if new information becomes available?

If any new information about your treatment becomes available during the research trial, your doctor will discuss this with you.

What will happen if I don't want to carry on with the research trial?

If you do not want to continue in the research trial, you can withdraw at any time without giving a reason. This will not affect your care in any way. If you decide to withdraw from the research trial, you can choose to have no further contact from us.

What will happen to the results of this research trial?

At the end of the research trial, the university trial team will prepare and publish a report. The results of the research trial will be publicly available on the CERM trial website at www.warwick.ac.uk/cerm. The results will be available to the hospitals that took part in the research trial.

The results of the research trial may be presented at scientific meetings and published in scientific journals. The university trial team will also share the results of the research trial with the Royal College of Obstetricians and Gynaecologists, who publish guidance on the best care

For peer review only - http://bmjopen.bmj.com/site/about/guidelines.xhtml

[**Print on Trust Headed Paper**]

for women across the world. You will not be identified in any reports or publications and none of the information will be able to be traced to you personally.

Who is organising and funding this research trial?

This research trial is funded by the National Institute for Health Research (NIHR) in partnership with the Medical Research Council (MRC) under the Efficacy and Mechanism Evaluation Programme 17/60/22. The Government set up the NIHR in 2006 to provide organised funding for research within the NHS.

University Hospital Coventry and Warwickshire is sponsoring the research trial. This covers the insurance costs that apply to research trials. Professor Siobhan Quenby (from University Hospital Coventry, Warwickshire NHS Trust and The University of Warwick) is the Chief Investigator and has overall responsibility for the research trial. The University of Warwick Clinical Trials Unit is organising the administration of the research trial.

Who has reviewed this research trial?

1. Reviewed and commissioned by the National Institute for Health Research - Efficacy and Mechanism Evaluation Programme.
2. Reviewed and approved by North West-Haydock Research Ethics Committee (the REC) on 20/08/2019. The REC are an independent group of people who review all research carried out in the NHS to protect your safety, rights, well-being and dignity.
3. Reviewed and authorised by the Medicines and Healthcare products Regulatory Agency (MHRA) on 20/08/2019. The MHRA is the government body that authorises clinical trials of medicines.
4. Reviewed and approved by the Quality Assurance team at Warwick Clinical Trials Unit.
5. Reviewed and approved by the Research and Development Office at your local NHS trust.
6. Reviewed by the patient and public involvement (PPI) representative on this trial.

How can I contact the hospital trial team?

If you have any questions about the research trial or your involvement in it, either now or in the future, you can contact the hospital trial team.

Hospital trial team
Email: [insert number]
Phone: [insert number]
Write to: [insert number]

Information about the CERM trial, and other useful information, is available from the CERM trial website at www.warwick.ac.uk/cerm.

Thank you for taking the time to read this information sheet.

For peer review only - http://bmjopen.bmj.com/site/about/guidelines.xhtml

[Print on Trust Headed Paper]

DoH Disclaimer and **Funding acknowledgment** - This project (project reference 17/60/22) is funded by the Efficacy and Mechanism Evaluation (EME) Programme, an MRC and NIHR partnership. The views expressed in this publication are those of the authors and not necessarily those of the MRC, NIHR or the Department of Health and Social Care.

For peer review only - http://bmjopen.bmj.com/site/about/guidelines.xhtml

CERM Chronic Endometritis and Recurrent Miscarriage

[Print on Trust Headed Paper]

Crystal Mark 23136 Clarity approved by Plain English Campaign

Participant information sheet (CERM B)

Information for women taking part in the chronic endometritis and recurrent miscarriage (CERM) trial

Trial title

Chronic endometritis and recurrent miscarriage – the CERM trial

This information sheet is available in **large print** from the university trial team (email: cerm@warwick.ac.uk).

Introduction

You are invited to take part in a research trial. Please read this information sheet carefully before deciding whether to take part. It explains why this research trial is being done and what it means for you if you decide to take part. You will have the opportunity to talk to a member of the hospital research team about this trial and they will be happy to answer any questions you have. You can also discuss the trial with your GP or the obstetrician or gynaecologist caring for you.

This leaflet is divided into two parts. **Part 1** tells you the purpose of the trial and what will happen to you if you take part. **Part 2** gives you more detailed information about how the trial will be carried out.

Part 1

What is the purpose of the research trial?

The aim of this research trial is to find out if antibiotics can reduce miscarriage. In some women the lining of the womb (the endometrium) is inflamed. Researchers have found a link between this and miscarriage. A healthy endometrium is important for the embryo to be able to attach to the womb. It is thought that endometritis disrupts this process, and can lead to a miscarriage. Treating endometritis with antibiotics may reduce the inflammation and the likelihood of a miscarriage. This has not been tested. This research trial will test this theory by comparing a 14-day course of an antibiotic (doxycycline) against a placebo (a 'dummy treatment' which will look exactly the same as the antibiotic but contains no active ingredients) to find out if taking antibiotics reduces miscarriages. The trial will be 'double blind'. This means that the women and the trial researchers will not know who is taking the antibiotic capsules and who is taking the placebo. It will also be a randomised controlled trial. This means, if you decide to take part, which capsules you receive (the antibiotic or the placebo) will be decided by chance. The trial will take place in NHS hospitals in the United Kingdom and will involve over 3,000 women who have recurrent miscarriage.

> Inflammation of the lining of the womb is a condition called **endometritis.**

> **Recurrent miscarriage** means two or more miscarriages in a row.

For peer review only - http://bmjopen.bmj.com/site/about/guidelines.xhtml

For peer review only

[Print on Trust Headed Paper]

Not everyone who has recurrent miscarriage has endometritis, so before you can take part in the randomised controlled trial the hospital trial team will need to take an endometrial biopsy (a small sample of tissue from your endometrium) and examine it under a microscope to find out if you do. We (the university research team) estimate that half the women tested will have endometritis and so be able to take part in the randomised controlled trial.

Researchers also suspect that inflammation of the endometrium may be caused by an imbalance of the microbiome that lives in the reproductive tract (the vagina, cervix, womb, fallopian tubes and ovaries). Another part of the trial is to look at the endometrium and microbiome to see how antibiotics affect these. A diagram of this research trial is shown in figure 1.

The **microbiome** is the name given to all the microbes that live in and on our bodies. It is mostly made up of bacteria, and everyone's microbiome is unique.

For peer review only

For peer review only - http://bmjopen.bmj.com/site/about/guidelines.xhtml

CERM
Chronic Endometritis
and Recurrent Miscarriage

[Print on Trust Headed Paper]

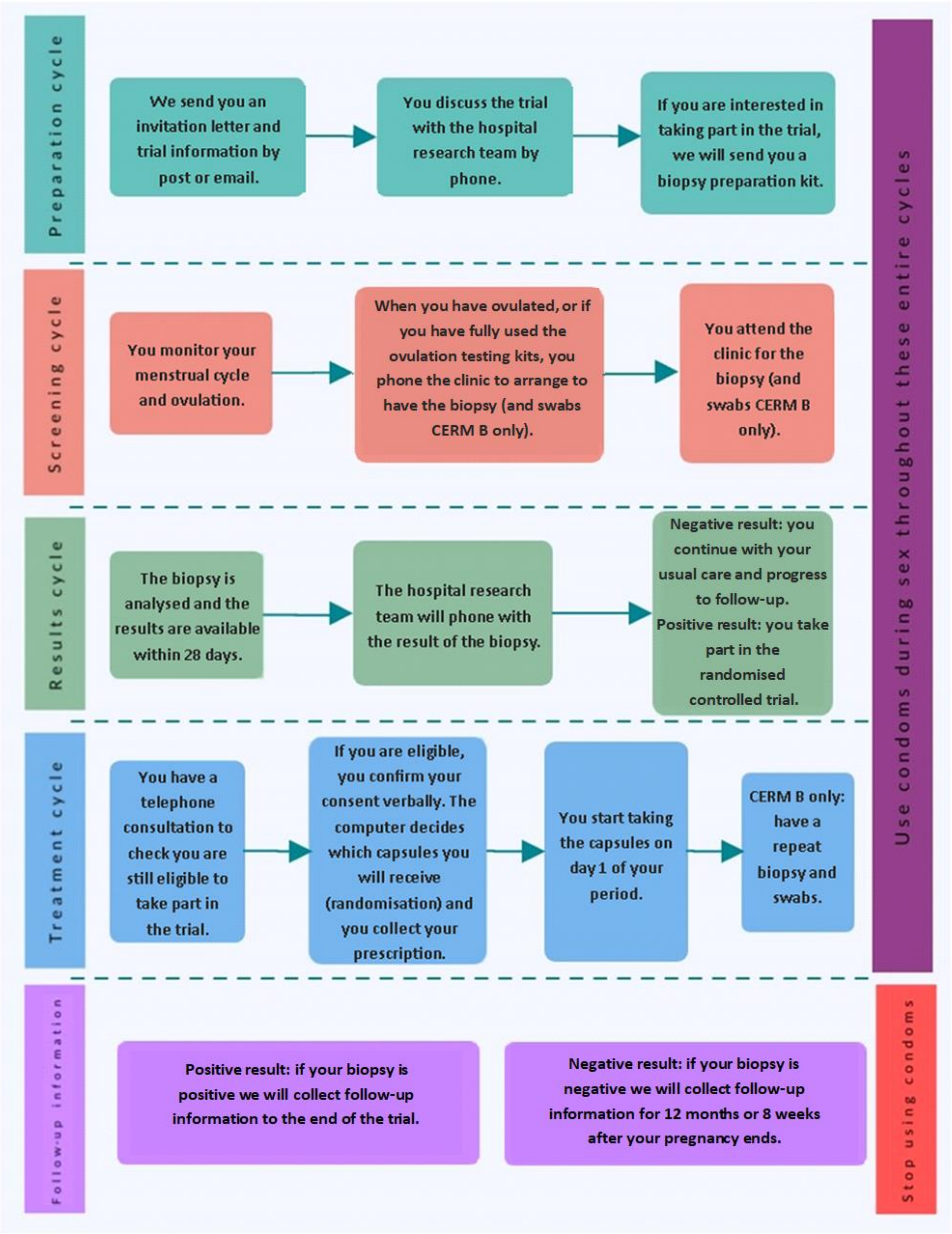

Figure 1 – an overview of the CERM trial

For peer review only - http://bmjopen.bmj.com/site/about/guidelines.xhtml

[Print on Trust Headed Paper]

Screening trial

Why have I been invited?

You have been invited to take part in this research trial because you have had two or more miscarriages (recurrent miscarriage).

Do I have to take part?

Taking part in the research trial is entirely voluntary. It is up to you whether or not you take part. You do not have to take part, and there will be no difference in the care you receive if you choose not to take part. If you want to take part, you will be asked to sign a consent form to confirm you have agreed to take part. You will keep a signed copy of the form. Even after signing the consent form, you can withdraw from the trial at any time if you change your mind, without having to give a reason. This will not affect the care you receive.

This information sheet will now explain what will happen if you decide to take part in this trial and have an endometrial biopsy and vaginal, cervical and endometrial swabs taken.

What will happen next?

A member of the hospital research team will talk to you over the phone (or speak to you face-to-face if you prefer this and if it is possible) to explain what the trial is about and what taking part would mean for you. You can ask the hospital research team any questions you have.

What will happen if I agree to take part?

1. **We will check to see if you are eligible –** You must be aged 18 to 41 years old. The hospital research team will ask about your obstetric and medical history to see if you are eligible to take part in the research trial. If you have not had any investigations to find out the cause of your recurrent miscarriage, these will be done when you attend the clinic. If a cause for your miscarriages is found, you will not be eligible for the trial. An important part of the eligibility check is to find out if you are willing to use condoms whenever you have sex throughout the entire menstrual cycles when you are:
    - preparing for your biopsy and swabs;
    - waiting for the result of your biopsy; and
    - taking the capsules and having your repeat biopsy and swabs.

   Doxycycline, the antibiotic used in the study, can be passed on in breast milk. The effects this could have on babies are not known. For this reason, and to make sure the study is absolutely safe, you will not be able to join the trial if you are breastfeeding.

2. **We will ask for permission to send you a 'biopsy preparation kit' –** If you are eligible to have a biopsy taken, the hospital research team will ask for your verbal consent to post a biopsy preparation kit to you. If you agree, this will be recorded in your hospital case notes and you will be given a trial screening number. The kit contains instructions, a period tracker, condoms and an ovulation testing kit. If you are prescribed a course of antibiotics between giving verbal consent and receiving the biopsy preparation kit, or

For peer review only - http://bmjopen.bmj.com/site/about/guidelines.xhtml

while you are using the biopsy preparation kit, please contact a member of the hospital research team (see the section: How can I contact the hospital trial team?). The biopsy may need to be delayed and the hospital team may need to send you another biopsy preparation kit once you have finished your course of antibiotics.

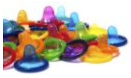

3. **Preparing for the biopsy and swabs –** It is very important that you **do not** try to conceive and that you use condoms whenever you have sex throughout this entire menstrual cycle. Condoms are provided in the kit and you can get more by contacting the hospital research team or family-planning services.

Starting on the first day of your next menstrual cycle after receiving the kit, you should use the period tracker to record:
- the days of your period; and
- the results of the ovulation testing.

The biopsy and swabs need to be taken a few days after you have ovulated. Ovulation usually happens around day 12 to 16, depending on the length of your cycle. We provide a period tracker and ovulation testing kit to help you work out the best time to have the biopsy and swabs taken. When the ovulation test shows you have ovulated, or if you have used all of the kit and it does not show you have ovulated, you will need to contact the hospital research team to arrange an appointment to come in for your biopsy and swabs.

4. **Having the biopsy and swabs –** Please bring your period tracker with you when you come to the clinic for your biopsy and swabs. In the clinic a doctor will explain what will happen and check you are eligible to take part in the trial. You will have the opportunity to ask them any questions you might have. You are welcome to bring your partner, a family member or a friend to this appointment, if the local rules on visitors to the clinic allow this. If you are eligible and would like to take part, you will be asked to sign a consent form. You will also be asked to take a pregnancy test before the biopsy. A healthcare professional will ask you for a sample of urine and they will test it. If the test shows you are pregnant, the biopsy and swabs will be cancelled, because it could put the pregnancy at risk, and you will be referred to your GP who will arrange for your care. If the pregnancy test shows you are not pregnant, you will register for the trial and be given a trial identification (ID) number. A doctor will then explain the endometrial biopsy procedure.

Before the procedure you will have a vaginal examination to find out the position of your womb so the healthcare professional can take the biopsy and swabs. The biopsy and swabs usually take a couple of minutes. First the healthcare professional will take swabs from your vagina, cervix and endometrium. They will send the swabs, and clinical data that does not identify you to the laboratory at Imperial College London, who will look at the microbiome to see what microbes are present. With your permission any material that is left over from the swabs, and clinical data that does not identify you, will be stored in the Tommy's National Reproductive Health Biobank. As this part of the trial is to look at how chronic endometritis may cause miscarriage, you will not get any results from these swabs.

For peer review only

For peer review only - http://bmjopen.bmj.com/site/about/guidelines.xhtml

CERM Chronic Endometritis and Recurrent Miscarriage

[Print on Trust Headed Paper]

The healthcare professional will then take the biopsy by passing a thin plastic tube through your cervix and into your womb. We will take a small sample of the lining of your womb. We will send part of this sample to the laboratory at the University Hospitals Coventry and Warwickshire NHS Trust (UHCW) to be analysed under the microscope to find out whether or not you have endometritis. The sample sent to UHCW for analysis will be labelled with your trial ID number and initials, not your name. With your permission, any tissue that is left over following the analysis, and clinical data that does not identify you, will be stored in the Tommy's National Reproductive Health Biobank and used in future ethically approved research.

The healthcare professional will do all they can to make sure they collect enough endometrial tissue to be analysed, but in a few cases this may not be possible. If the healthcare professional is not able to collect a sample or cannot collect enough tissue to be analysed, depending on the reason for this, the health professional carrying out the biopsy will discuss the following options with you.

- The same healthcare professional will repeat the biopsy during the same appointment
- A different healthcare professional will take the biopsy during the same appointment
- You come back to the clinic for another biopsy during your next menstrual cycle
- You choose not to repeat the biopsy and withdraw from the trial

After the biopsy you will be given a pregnancy test kit to take home.

5. **You get your biopsy results and the next steps** – While you are waiting for your biopsy results, it is important that you continue to use condoms whenever you have sex. We will phone you when your biopsy results are available. This will usually be within four weeks.

   If your results show that you do not have endometritis, you will continue with your usual care.
   - We will phone you at three, six and 12 months to see if you are still trying to get pregnant, if you are pregnant or if you have had any miscarriages or terminations. If you get pregnant between our phone calls, please let the hospital research team know (see the section: How can I contact the hospital trial team?).
   - If you get pregnant, we will collect follow-up information from the scans you have during your pregnancy.
   - If you have a baby, we will phone you eight weeks after the birth to ask about your health and your baby's health. The details we ask for will include the date of the birth, how many weeks pregnant you were at the time of the birth, how your baby was delivered, and your baby's weight and sex.
   - We will ask for details of any complications and abnormalities (for both you and your baby).

If your results show that you have endometritis, you may be eligible to take part in the randomised controlled trial. The following section explains what will happen during this stage.

For peer review only - http://bmjopen.bmj.com/site/about/guidelines.xhtml

[Print on Trust Headed Paper]

It is very important that you use condoms whenever you have sex during this entire menstrual cycle, and not just when you are taking the capsules.

Randomised controlled trial

A member of the hospital research team will phone you to explain that your results show you have endometritis and you may be eligible to take part in the randomised controlled trial. They will explain what this part of the trial is about and what taking part would mean for you. You can ask the hospital research team any questions you have.

For peer review only

What will happen if I agree to take part?

1. **We will check to see if you are still eligible.** We will make an appointment for you to have a consultation with a member of the hospital research team. The consultation may take place face-to-face or by phone or video call. You can ask any questions you have and you can ask your partner, a family member or a friend to join the consultation.

   During your consultation a doctor will ask you about your medical and obstetric history to check you are eligible to take part in the randomised controlled trial. If investigations have revealed a treatable cause for your recurrent miscarriage since you had the endometrial biopsy, you will not be eligible for the trial and a doctor will discuss your treatment options with you.

   An important part of the eligibility check is to find out if you are still willing to use condoms whenever you have sex throughout your entire menstrual cycle when you are taking the capsules.

   You will be asked to do a pregnancy test (using the pregnancy test kit provided at your biopsy visit) **and** call the hospital research team with the result. If the test shows you are pregnant, you will not be eligible for the trial and you will be referred to your GP who will arrange your care.

   Following the eligibility check, the doctor will ask you to confirm that you are happy to take part in the randomised controlled trial. You are free to withdraw from the trial at this stage or any point, for any reason.

2. **Randomisation –** Whether you will receive doxycycline or the placebo is decided by chance. You and the trial researchers will not know who is taking the doxycycline and who is taking the placebo, but it will be possible to find this out if it becomes necessary for your clinical care.

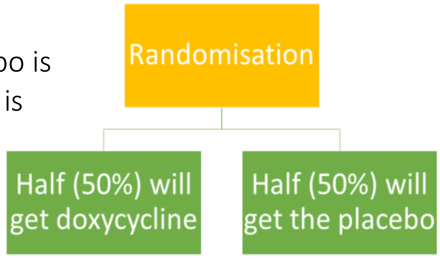

For peer review only - http://bmjopen.bmj.com/site/about/guidelines.xhtml

CERM Chronic Endometritis and Recurrent Miscarriage

[Print on Trust Headed Paper]

3. **Collecting your prescription and taking the capsules –** You can collect the capsules from a member of the hospital research team or from the hospital pharmacy (after you have collected your prescription from the hospital research team) at an agreed time that is convenient to you.

   You will be given an information booklet with the capsules. The booklet gives full instructions on how to take the capsules and you should read it carefully. It has guidance on what to do if something happens during the trial, and what precautions you should take while you are taking the capsules.

   You can start taking the capsules on the first day of your next menstrual cycle. The dose is one capsule twice a day, 12 hours apart, for 14 days. Please fill in the treatment diary and mark a cross in the diary every time you take a capsule.

   These capsules contain gelatine and small amounts of lactose.

   You will be given a CERM trial participant card. It is very important that you carry your trial participant card with you at all times while you are taking the capsules, and show it to anyone you are receiving healthcare from. Throughout this entire menstrual cycle, it is important that you use condoms whenever you have sex.

4. **Preparing for the repeat biopsy and swabs –** The procedure is identical to the first biopsy and swabs you had taken, and the instructions are repeated for you below. You can choose not to come for the second biopsy for any reason.

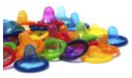

It is very important that you **do not** try to conceive and you use condoms whenever you have sex throughout the entire menstrual cycle while you are taking the capsules and preparing for your repeat biopsy and swabs. Condoms were provided in the biopsy preparation kit and you can get more by contacting the hospital research team or family-planning services.

Starting on the first day of your next menstrual cycle you should use the period tracker to record:

   a. the days of your period; and
   b. the results of the ovulation testing.

The repeat biopsy and swabs need to be taken a few days after you have ovulated. Ovulation usually happens around day 12 to 16, depending on the length of your cycle. We provide a period tracker and ovulation testing kit to help you work out the best time to have the biopsy and swabs taken. When the ovulation test shows you have ovulated, or if you have used all of the kit and it does not show you have ovulated, you will need to contact the hospital research team to arrange an appointment to come in for your biopsy and swabs.

For peer review only - http://bmjopen.bmj.com/site/about/guidelines.xhtml

[Print on Trust Headed Paper]

5. **Having the repeat biopsy and swabs –** Please bring your period tracker with you when you come to the clinic for your repeat biopsy and swabs. In the clinic a member of the hospital research team will explain what will happen. You will have the opportunity to ask them any questions you might have. You are welcome to bring your partner, a family member or a friend to this appointment, if the local rules on visitors to the clinic allow this. You will also be asked to take a pregnancy test. If the test shows you are pregnant, the biopsy and swabs will not be taken and details of your pregnancy will be recorded. If the pregnancy test shows you are not pregnant, a doctor will then explain the endometrial biopsy and swab procedure.

   Before the procedure you will have a vaginal examination to find out the position of your womb so the healthcare professional can take the biopsy and swabs. The biopsy and swabs usually take a couple of minutes. First the healthcare professional will take swabs from your vagina, cervix and endometrium. They will send the swabs, and clinical data that does not identify you, to the laboratory at Imperial College London, who will look at the microbiome to see what microbes are present. With your permission any material that is left over from the swabs, and clinical data that does not identify you, will be stored in the Tommy's National Reproductive Health Biobank.

   The healthcare professional will then take the biopsy by passing a thin plastic tube through your cervix and into your womb. We will take a small sample of the lining of your womb. We will send part of this sample to the laboratory at the University Hospitals Coventry and Warwickshire NHS Trust (UHCW) to be analysed under the microscope. The sample will be labelled with your trial ID number and initials, not your name. With your permission, any tissue that is left over following the analysis and clinical data that does not identify you, will be stored in the Tommy's National Reproductive Health Biobank and used in future ethically approved research. You will not receive the results of the biopsy or swabs, but they will allow us to find out the effect of the capsules.

   The healthcare professional will do all they can to make sure they collect enough endometrial tissue to be analysed, but in a few cases this may not be possible. If the healthcare professional is not able to collect a sample or cannot collect enough tissue to be analysed, the biopsy will not be performed again.

   When you attend for your repeat biopsy and swabs we will ask you if you have taken all of your capsules as prescribed and if you have had any side effects. Please bring your treatment diary and any leftover capsules to this visit.

6. **If you miss your next period or suspect you are pregnant** - Take a home pregnancy test. Follow the instructions that come with the test. Contact the hospital research team if the pregnancy test is positive or if you are unsure of the result.

Follow-up information

We will only collect information about you and your baby that is relevant to taking part in the CERM research trial. We will ask you for this information, but if you are not sure of, or don't

For peer review only - http://bmjopen.bmj.com/site/about/guidelines.xhtml

CERM Chronic Endometritis and Recurrent Miscarriage

[Print on Trust Headed Paper]

know, the details we need we will refer to your hospital records (at whichever hospital you receive care from during the trial) or GP records (or both).

- We will phone you after you have taken all of your capsules to see if you have taken all of them as prescribed and if you have had any side effects. Please refer to your treatment diary for this information.
- We will phone you at three, six, 12 and 24 months plus at the end of the trial to see if you are still trying to get pregnant, if you are pregnant or if you have had any miscarriages or terminations. If you get pregnant between our phone calls, please let the hospital research team know (see the section: How can I contact the hospital trial team?).
- If you get pregnant, we will collect follow-up information from the scans you have during your pregnancy.
- If you have a baby, we will phone you eight weeks after the birth to ask about your health and your baby's health. The details we ask for will include the date of the birth, how many weeks pregnant you were at the time of the birth, how your baby was delivered, and your baby's weight and sex.
- We will ask for details of any complications, abnormalities, infections, investigations and results, treatment and hospital stays (for both you and your baby).

Expenses and payments

There are no payments for taking part in this research trial. We will refund any hospital parking charges you have to pay for hospital appointments that relate to this research.

What are the clinical alternatives?

Currently there are no clinical alternatives, as few treatments have been shown to prevent miscarriage.

What are the possible disadvantages and risks of taking part?

You and your partner will need to delay trying to conceive because it is important that you use condoms whenever you have sex throughout your entire menstrual cycles when you are:

- preparing for your biopsy and swabs
- waiting for the result of your biopsy
- taking the capsules; and
- preparing for your repeat biopsy and swabs.

We have streamlined the trial processes to make sure the time you need to delay trying to conceive is as short as possible.

Some women find the endometrial biopsy is painful and may get cramping at the time of the biopsy. Taking paracetamol and ibuprofen an hour before the biopsy can help with this. If you need it, gas and air (Entonox) will be available while you are having the biopsy. Some women

For peer review only - http://bmjopen.bmj.com/site/about/guidelines.xhtml

CERM Chronic Endometritis and Recurrent Miscarriage

[Print on Trust Headed Paper]

may have some vaginal bleeding (spotting) after the biopsy is taken. This will stop quickly on its own.

Please see the 'Taking my capsules on the chronic endometritis and recurrent miscarriage (CERM) trial' leaflet for details of any possible side effects while you are taking the capsules, and what to do if something happens when you are taking them.

What are the possible benefits of taking part?

We do not know if taking part in the research trial would benefit you personally. Taking part will show whether you have chronic endometritis. The results will help us provide advice on treatment options for women who have recurrent miscarriage.

What happens when the research trial stops?

The research trial is planned to take up to 48 months to complete.

Whenever the research trial stops, the university trial team will analyse the information collected to decide if antibiotics should be prescribed to women with chronic endometritis. In the future, these results will help women who are experiencing recurrent miscarriage.

This is the end of Part 1.

If you are interested in taking part in the trial, please read the important information in Part 2 before making your decision.

Part 2

What if there is a problem?

If you have any concerns about any aspect of this study, you should ask to speak to the researchers who will do their best to answer your questions. If you are still not happy and want to make a complaint, you can do this through the NHS complaints procedure.

Please write to:

Complaints Manager
University Hospitals of Coventry and Warwickshire NHS Trust
Clifford Bridge Road
CV2 2DX

Or phone: 02476 965 198

In the unlikely event that you are harmed by taking part in this study, compensation may be available. If you suspect that the harm is the result of someone's negligence, you may be able to take legal action, but you may have to pay any costs involved and you should get legal advice about this.

For peer review only - http://bmjopen.bmj.com/site/about/guidelines.xhtml

[Print on Trust Headed Paper]

For independent advice on research, you can contact PALS (Patient Advice and Liaison Service) on Freephone 0800 028 4203, or you can email them at feedback@uhcw.nhs.uk.

What Covid-19 precautions should I take when coming to hospital for the trial?
Your local NHS trust may have introduced measures to reduce your risk of being exposed to Covid-19. Please see the relevant hospital's website or ask the hospital trial team for more information.

Should I have the Covid-19 vaccine while I am taking part in the trial?
The CERM trial team, which includes clinicians and a pharmacist, have confirmed that there are no interactions between the drug used in the trial (doxycycline) and available Covid-19 vaccines. This means there are no safety concerns with you having a Covid-19 vaccine if you are offered one while you are taking part in the trial.

The hospital research team will ask you to let them know if you do have a Covid-19 vaccine so they can record the details alongside any other medications you are taking. If you have any questions, please get in touch with the hospital research team or your GP.

How will my information be used?
University Hospitals Coventry and Warwickshire NHS Trust (UHCW) is the sponsor for this trial in the United Kingdom. The trial will be managed by Warwick Clinical Trials Unit at the University of Warwick (UoW). UHCW and the UoW will use information you provide and information from your hospital records and your GP records to carry out this trial, and will act as joint data controllers for the trial. This means that, together, they are responsible for looking after your information and using it properly.

We will only collect information about you and your baby that is relevant to taking part in the CERM research trial. We will ask you for this information, but if you are not sure of, or don't know, the details we need we will refer to your hospital records (at whichever hospital you receive care from during the trial) or GP records (or both). The information we will collect is listed below.

- The name of any investigations you have had
- The results of any investigations you have had
- The name of any illnesses, conditions or dependencies you have or have had
- The name of any prescription medications you are taking, the dose or units, and how long you have been taking them
- Details of any pregnancies, miscarriages or terminations
- The results of any pregnancy scans
- Details of any births, including the date, how many weeks pregnant you were at the time of the birth, how your baby was delivered, whether your baby was born alive or was stillborn, and your baby's weight and sex
- Details of any complications, abnormalities, infections, investigations and results, treatment and hospital stays (for both you and your baby)

For peer review only - http://bmjopen.bmj.com/site/about/guidelines.xhtml

CERM Chronic Endometritis and Recurrent Miscarriage

[Print on Trust Headed Paper]

- The results from the routine genetic analysis of any miscarriage tissue and tissue from the placenta (we will only record these if you choose to take part in the randomised controlled trial)

Your hospital will collect information from you and your medical records and will pass this to us for this research trial in line with our instructions. This information will include your initials and date of birth. We would also like to collect details of your ethnic background and race, but you can choose not to provide this information if you prefer. We will ask for your permission to tell your GP that you are taking part in the research trial. If you do not want us to tell your GP, you will not be able to take part.

Your hospital will keep your name, NHS number and contact details confidential and will not pass this information to UHCW or the UoW. Your hospital will use this information, as needed, to contact you about the research trial, to make sure that relevant information about the trial is recorded for your care, and to oversee the quality of the trial. Certain people from UHCW, the UoW and regulatory organisations may look at your medical and research records to check that the research trial is accurate. UHCW and the UoW will only receive information that does not contain any details that directly identify you. The people who analyse the information will not be able to identify you and will not be able to find out your name, NHS number or contact details. UHCW and the UoW will keep identifiable information about you for 10 years after the trial has finished (if the results of your biopsy show that you **do not** have endometritis) or for 25 years after the trial has finished (if the results of your biopsy show that you **do** have endometritis).

When you agree to take part in a research trial, the information about your health and care may be provided to researchers running other research studies in this organisation and in other organisations. These organisations may be universities, NHS organisations or companies involved in health and care research in this country or abroad. Organisations and researchers will only use your information to carry out research in line with the UK Policy Framework for Health and Social Care Research. This information will not identify you and will not be combined with other information in a way that could identify you. The information will only be used for the purpose of health and care research. It will not affect your care, and organisations and researchers cannot use it to contact you. Your information will not be used to make decisions about future services that are available to you, such as insurance.

Your rights to see, change or move your information are limited, as your information is managed in specific ways to make sure the research is reliable and accurate. If you withdraw from the trial, the information that has already been collected about you will be kept. To protect your rights, as few details as possible that could identify you will be collected.

To find out more about how your information is handled, you can visit the privacy notices of the data controllers.
www.uhcw.nhs.uk/privacy/
www.warwick.ac.uk/services/idc/dataprotection/privacynotices/researchprivacynotice

Will my information be kept confidential?

For peer review only - http://bmjopen.bmj.com/site/about/guidelines.xhtml

CERM
Chronic Endometritis and Recurrent Miscarriage

[Print on Trust Headed Paper]

Yes. All information collected about you is strictly confidential. Any research information UHCW and the UoW collect will refer to you only by a unique trial ID number and your initials, so the risk of you being identified is very low. All your information will be stored securely and held at the Warwick Clinical Trials Unit, in line with all relevant UK laws.

What if new information becomes available?

If any new information about your treatment becomes available during the research trial, your doctor will discuss this with you.

What will happen if I don't want to carry on with the research trial?

If you do not want to continue in the research trial, you can withdraw at any time without giving a reason. This will not affect your care in any way. If you decide to withdraw from the research trial, you can choose to have no further contact from us.

What will happen to the results of this research trial?

At the end of the research trial, the university trial team will prepare and publish a report. The results of the research trial will be publicly available on the CERM trial website at www.warwick.ac.uk/cerm. The results will be available to the hospitals that took part in the research trial.

The results of the research trial may be presented at scientific meetings and published in scientific journals. The university trial team will also share the results of the research trial with the Royal College of Obstetricians and Gynaecologists, who publish guidance on the best care for women across the world. You will not be identified in any reports or publications and none of the information will be able to be traced to you personally.

Who is organising and funding this research trial?

This research trial is funded by the National Institute for Health Research (NIHR) in partnership with the Medical Research Council (MRC) under the Efficacy and Mechanism Evaluation Programme 17/60/22. The Government set up the NIHR in 2006 to provide organised funding for research within the NHS.

University Hospital Coventry and Warwickshire is sponsoring the research trial. This covers the insurance costs that apply to research trials. Professor Siobhan Quenby (from University Hospital Coventry, Warwickshire NHS Trust and The University of Warwick) is the Chief Investigator and has overall responsibility for the research trial. The University of Warwick Clinical Trials Unit is organising the administration of the research trial.

Who has reviewed this research trial?

1. Reviewed and commissioned by the National Institute for Health Research - Efficacy and Mechanism Evaluation Programme.
2. Reviewed and approved by North West-Haydock Research Ethics Committee (the REC) on 20/08/2019. The REC are an independent group of people who review all research carried out in the NHS to protect your safety, rights, well-being and dignity.

For peer review only - http://bmjopen.bmj.com/site/about/guidelines.xhtml

CERM Chronic Endometritis and Recurrent Miscarriage

[Print on Trust Headed Paper]

3. Reviewed and authorised by the Medicines and Healthcare products Regulatory Agency (MHRA) on 20/08/2019. The MHRA is the government body that authorises clinical trials of medicines.
4. Reviewed and approved by the Quality Assurance team at Warwick Clinical Trials Unit.
5. Reviewed and approved by the Research and Development Office at your local NHS trust.
6. Reviewed by the patient and public involvement (PPI) representative on this trial.

How can I contact the hospital trial teams?

If you have any questions about the research trial or your involvement in it, either now or in the future, you can contact the hospital trial team.

Hospital trial team
Email: [insert number]
Phone: [insert number]
Write to: [insert number]

Information about the CERM trial, and other useful information, is available from the CERM trial website at www.warwick.ac.uk/cerm.

Thank you for taking the time to read this information sheet.

DoH Disclaimer and **Funding acknowledgment** - This project (project reference 17/60/22) is funded by the Efficacy and Mechanism Evaluation (EME) Programme, an MRC and NIHR partnership. The views expressed in this publication are those of the authors and not necessarily those of the MRC, NIHR or the Department of Health and Social Care.

JOINTLY FUNDED BY UKRI Medical Research Council NIHR National Institute for Health and Care Research

WARWICK CLINICAL TRIALS UNIT | The Lily Mae Foundation | Imperial College London | UNIVERSITY OF BIRMINGHAM | Tommy's | NHS University Hospitals Coventry and Warwickshire NHS Trust

For peer review only - http://bmjopen.bmj.com/site/about/guidelines.xhtml

CERM Chronic Endometritis and Recurrent Miscarriage

Print on Trust headed paper

Participant consent form (CERM A)

Permission to take part in the chronic endometritis and recurrent miscarriage (CERM) trial

This form is available in **large print** from the trial team. Email **cerm@warwick.ac.uk**

Make three copies: Keep the original in the CERM trial investigator site file, give one copy to the participant, and put one in the participant's hospital medical notes.

Trial ID number					Principal investigator		Site	

**1.** I have read and understood the CERM A participant information sheet version [insert number] dated [dd/mm/yyyy].	Please initial
**2.** I have had time to think about the information I have been given about the CERM trial and ask questions, and I am satisfied with the answers I have been given.	Please initial
**3.** I understand that I do not have to take part and that I can withdraw from the CERM trial at any time, without giving a reason and without my medical care or legal rights being affected.	Please initial
**4.** I understand that the hospital research team will ask me for information about my past and current health that is relevant to me taking part in the CERM research trial. I give the hospital research team permission to collect and record this information.	Please initial
**5.** I understand that the hospital research team will collect information from my NHS hospital and GP records if it is relevant to me taking part in the CERM trial. I give the research team permission to access these records to collect and record this information.	Please initial
**6.** I understand that the research team and trial monitors from Warwick Clinical Trials Unit, the hospital trusts and regulatory authorities may have access to my medical and trial records to monitor this trial. I give permission for these people to access and record this information.	Please initial
**7.** I confirm that my partner and I have used condoms during this menstrual cycle. I will continue to use condoms whenever I am having sex throughout my entire menstrual cycle while I am waiting for the result of my biopsy and, if I take part in the randomised trial, while I am taking the capsules.	Please initial
**8.** I have seen the result of my pregnancy test taken today and confirm that it showed that I am not pregnant.	Please initial
**9.** I agree to have an endometrial biopsy taken.	Please initial
**10.** I agree to my endometrial biopsy being held in the Tommy's National Reproductive Health Biobank until it is analysed.	Please initial
**11. Optional:** I agree that any tissue that is left over following my endometrial biopsy or after my endometrial biopsy is analysed can be stored in the Tommy's National Reproductive Health Biobank, **with clinical data that does not identify me,** and used in future ethically approved research.	Please initial
**12.** I agree to the hospital trial team storing my phone numbers so they can contact me with the results of my endometrial biopsy and to discuss the next steps.	Please initial
**13.** I agree to the hospital trial team storing my phone numbers and contacting me for follow-up information as explained in the participant information sheet (or sheets).	Please initial
**14.** I agree to the research team telling my GP that I am taking part in the CERM research trial.	Please initial
**15.** I agree to take part in the CERM research trial.	Please initial

Please go to page 2.

Crystal Mark 23120 Clarity approved by Plain English Campaign

For peer review only - http://bmjopen.bmj.com/site/about/guidelines.xhtml

For peer review only

Print on Trust headed paper

We will only use the following consent if you later take part in the randomised controlled trial.

16. I understand that I should not take doxycycline while I am pregnant because it can affect the baby. I understand it is important to use condoms during my entire menstrual cycle when I am taking doxycycline or a placebo (whichever I am given).	Please initial
17. I agree to take doxycycline or a placebo for 14 days and only as instructed.	Please initial
18. I understand that if I give birth at another hospital, the hospital research team will contact the relevant hospital trust to collect medical details from my NHS hospital records (and my baby's) if this is relevant to taking part in the CERM research trial. I give the research team permission to access these records to collect and record this information.	Please initial
19. I understand that the hospital research team will collect information from my baby's NHS hospital and GP records if it is relevant to me taking part in the CERM trial. I give the research team permission to access these records to collect and record this information.	Please initial
20. I understand that the research team and trial monitors from Warwick Clinical Trials Unit, the hospital trusts and regulatory authorities may have access to my baby's medical and trial records to monitor this trial. I give permission for these people to access and record this information.	Please initial
21. I agree to take part in the CERM randomised controlled trial.	Please initial

Your name Please print **Your signature** Please sign **Date** dd/mm/yyyy

Investigator's name Please print **Investigator's signature** Please sign **Date** dd/mm/yyyy

Crystal Mark 23120 Clarity approved by Plain English Campaign

For peer review only - http://bmjopen.bmj.com/site/about/guidelines.xhtml

For peer review only

CERM Chronic Endometritis and Recurrent Miscarriage

Print on Trust headed paper

Participant consent form (CERM B)

Permission to take part in the chronic endometritis and recurrent miscarriage (CERM) trial

This form is available in **large print** from the trial team. Email **cerm@warwick.ac.uk**

Make three copies: Keep the original in the CERM trial investigator site file, give one copy to the participant, and put one in the participant's hospital medical notes.

Trial ID number					Principal investigator		Site	

**1.** I have read and understood the CERM B participant information sheet version [insert number] dated [dd/mm/yyyy].	Please initial
**2.** I have had time to think about the information I have been given about the CERM trial and ask questions, and I am satisfied with the answers I have been given.	Please initial
**3.** I understand that I do not have to take part and that I can withdraw from the CERM trial at any time, without giving a reason and without my medical care or legal rights being affected.	Please initial
**4.** I understand that the hospital research team will ask me for information about my past and current health that is relevant to me taking part in the CERM research trial. I give the hospital research team permission to collect and record this information.	Please initial
**5.** I understand that the hospital research team will collect information from my NHS hospital and GP records if it is relevant to me taking part in the CERM trial. I give the research team permission to access these records to collect and record this information.	Please initial
**6.** I understand that the research team and trial monitors from Warwick Clinical Trials Unit, the hospital trusts and regulatory authorities may have access to my medical and trial records to monitor this trial. I give permission for these people to access and record this information.	Please initial
**7.** I confirm that my partner and I have used condoms during this menstrual cycle. I will continue to use condoms whenever I am having sex throughout my entire menstrual cycle while I am waiting for the result of my biopsy and, if I take part in the randomised trial, while I am taking the capsules and preparing for my repeat biopsy and swabs.	Please initial
**8.** I have seen the result of my pregnancy test taken today and confirm that it showed that I am not pregnant.	Please initial
**9.** I agree to have an endometrial biopsy taken.	Please initial
**10.** I agree to my endometrial biopsy being held in the Tommy's National Reproductive Health Biobank until it is analysed.	Please initial
**11. Optional:** I agree that any tissue that is left over following my endometrial biopsy or after my endometrial biopsy is analysed can be stored in the Tommy's National Reproductive Health Biobank, with clinical data that does not identify me,- and used in future ethically approved research.	Please initial
**12.** I agree to have cervical, vaginal and endometrial swabs taken.	Please initial
**13.** I agree to my cervical, vaginal and endometrial swabs being stored in the Tommy's National Reproductive Health Biobank until they are analysed.	Please initial
**14. Optional:** I agree that any material that is left over after my cervical, vaginal and endometrial swabs are analysed can be stored in the Tommy's National Reproductive Health Biobank, with clinical data that does not identify me, and used in future ethically approved research.	Please initial

Please go to page 2.

Crystal Mark 23121 Clarity approved by Plain English Campaign

For peer review only - http://bmjopen.bmj.com/site/about/guidelines.xhtml

CERM Chronic Endometritis and Recurrent Miscarriage

Print on Trust headed paper

**15.** I agree to the hospital trial team storing my phone numbers so they can contact me with the results of my endometrial biopsy and to discuss the next steps.	Please initial
**16.** I agree to the hospital trial team storing my phone numbers and contacting me for follow-up information as explained in the participant information sheet (or sheets).	Please initial
**17.** I agree to the research team telling my GP that I am taking part in the CERM research trial.	Please initial
**18.** I agree to take part in the CERM research trial.	Please initial

We will only use the following consent if you later take part in the randomised controlled trial.

**19.** I understand that I should not take doxycycline while I am pregnant because it can affect the baby. I understand it is important to use condoms during my entire menstrual cycle when I am taking doxycycline or a placebo (whichever I am given).	Please initial
**20.** I agree to take doxycycline or a placebo for 14 days and only as instructed.	Please initial
**21.** I understand that if I give birth at another hospital, the hospital research team will contact the relevant hospital trust to collect medical details from my NHS hospital records (and my baby's) if this is relevant to taking part in the CERM research trial. I give the research team permission to access these records to collect and record this information.	Please initial
**22.** I understand that the hospital research team will collect information from my baby's NHS hospital and GP records if it is relevant to me taking part in the CERM trial. I give the research team permission to access these records to collect and record this information.	Please initial
**23.** I understand that the research team and trial monitors from Warwick Clinical Trials Unit, the hospital trusts and regulatory authorities may have access to my baby's medical and trial records to monitor this trial. I give permission for these people to access and record this information.	Please initial
**24.** I agree to have a pregnancy test taken when I come to hospital for a repeat endometrial biopsy. I understand I will be shown the result of the test to confirm I am not pregnant.	Please initial
**25.** I agree to have a repeat endometrial biopsy taken.	Please initial
**26.** I agree to my repeat endometrial biopsy being stored in the Tommy's National Reproductive Health Biobank until it is analysed.	Please initial
**27. Optional:** I agree that any tissue that is left over after my repeat endometrial biopsy is analysed can be stored in the Tommy's National Reproductive Health Biobank, **with clinical data that does not identify me,** and used in future ethically approved research.	Please initial
**28.** I agree to have repeat cervical, vaginal and endometrial swabs taken.	Please initial
**29.** I agree to my repeat cervical, vaginal and endometrial swabs being stored in the Tommy's National Reproductive Health Biobank until they are analysed.	Please initial
**30. Optional:** I agree that any material that is left over after my repeat cervical, vaginal and endometrial swabs are analysed can be stored in the Tommy's National Reproductive Health Biobank, **with clinical data that does not identify me,** and used in future ethically approved research.	Please initial
**31.** I agree to take part in the CERM randomised controlled trial.	Please initial

Please go to page 3.

Crystal Mark 23121 Clarity approved by Plain English Campaign

For peer review only - http://bmjopen.bmj.com/site/about/guidelines.xhtml

For peer review only

Print on Trust headed paper

Your name Please print	Your signature Please sign	Date dd/mm/yyyy
Investigator's name Please print	Investigator's signature Please sign	Date dd/mm/yyyy

For peer review only

For peer review only - http://bmjopen.bmj.com/site/about/guidelines.xhtml

Crystal Mark 23121 Clarity approved by Plain English Campaign

Possible side effects

If you get any of the following side effects, stop taking the capsules. Go to an emergency department and take your CERM trial participant card with you.

- Chest pain
- Difficulty breathing
- Swollen eyelids, face or lips
- Very severe headaches
- Severe skin reaction
- Fever or swollen lymph nodes
- Diarrhoea with blood in it

If you get any of the following side effects and you are concerned, call the hospital research team on [insert number].

- Feeling or being sick
- Heartburn
- Indigestion (dyspepsia)
- Gastritis (being sick)
- Vaginal infection

Please note: Very few women will get these side effects.

About the CERM trial

This is a new research trial designed to see if taking antibiotics reduces miscarriage.

If a woman has two or more miscarriages in a row, this is known as recurrent miscarriage. Some women who have recurrent miscarriage have an inflammation of the lining of the womb (chronic endometritis) and it is thought that this might prevent the embryo from attaching to the wall of the womb (implantation).

This trial is designed to find out if a two-week course of antibiotics will treat the inflammation and reduce the likelihood of a miscarriage.

During the trial, you will be given capsules to take each day. These will either contain an antibiotic (doxycycline) or a placebo (a substance with no medicinal effect, used as a control in clinical trials).

How to contact us

Email: <<<Insert site contact email>>>
Phone: <<<insert site number>>>
Address: <<<Insert Site Address>>>
Website: www.warwick.ac.uk/cerm

CERM
Chronic Endometritis and Recurrent Miscarriage

Crystal Mark 23107
Clarity approved by Plain English Campaign

Taking my capsules on the chronic endometritis and recurrent miscarriage (CERM) trial

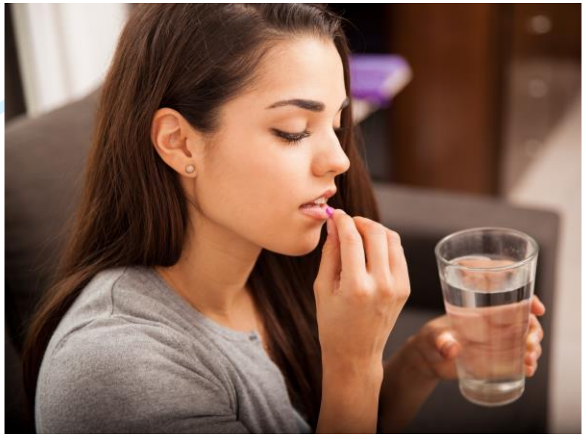

For peer review only - http://bmjopen.bmj.com/site/about/guidelines.xhtml

How to take the capsules

- Start taking the capsules on the first day of your period.
- Take **one** capsule **twice** a day.
- Swallow the capsules with plenty of water and take them with milk or food.
- Space the doses 12 hours apart and do not take them immediately before bedtime or if you are lying down.
- Keep taking this medicine until you finish the course, unless you are told to stop.
- Protect your skin from sunlight by using sunscreen, wearing appropriate clothing and spending time in the shade – even on a bright but cloudy day. Do not use sunbeds.
- **Do not** take indigestion remedies, or medicines which contain iron or zinc, two hours before or after you take this medicine.
- It is important to use condoms and not have unprotected sex during this entire menstrual cycle.
- It is important that you keep your CERM trial participant card with you at all times during the two weeks you are taking the capsules.

What to do if something happens when you are taking the capsules	
What if I forget to take a capsule?	Please do not double your dose. Take a capsule as soon as you remember, within six hours, and then take the next dose as scheduled. (If you don't remember until after six hours have passed, miss that dose – see below.) It is important that you finish the course.
What if I miss a capsule?	Please do not double your dose. Continue to take the capsules as prescribed and take the missed capsule at end of the course. It is important that you finish the course.
What if I miss more than one capsule?	Please do not double your dose. Continue to take the capsules as prescribed, and take the missed capsules at end of the course. It is important that you finish the course.
What if I lose my capsules?	If you lose your capsules we will not be able to replace them.
What if I take too many capsules?	Get medical advice immediately. Show your CERM trial participant card to any staff treating you. Stop taking the capsules. Tell the research team after your medical consultation.
What if I am sick after taking a capsule?	If you are sick within two hours of taking the capsule, you can take another one. If you are sick more than two hours after taking a capsule, take the next dose at the usual time.
What if I develop a rash?	If you develop a rash, stop taking the capsules and get medical advice. Show your CERM trial participant card to any staff treating you. Tell the research team after your medical consultation.
What if I have an allergic reaction?	If you have an allergic reaction, stop taking the capsules and get medical advice. Show your CERM trial participant card to any health staff treating you. Tell the research team after your consultation.
What if I find out I am pregnant when I'm taking the capsules?	If you find out you are pregnant while you are still taking the capsules, please stop taking them immediately and contact the research team. If you find you are pregnant after you have finished taking the capsules, please tell the research team.
Can I take the capsules with other medication?	Not all medications are safe to take with the capsules. If a health professional advises you to take a **new** medication, either prescribed or over the counter, show them your CERM trial participant card.
What if I get an infection and need to take antibiotics?	If you get an infection that needs antibiotics, show the healthcare provider your CERM trial patient card and follow their advice regarding stopping the trial capsules.
Can I drink alcohol when I am taking the capsules?	Do not drink alcohol when you are taking the capsules.
What if anything else happens?	If anything happens that is not listed here, call us for advice on [insert number]

For peer review only

SPIRIT | CONSORT OUTCOMES Reporting Guidelines for Trial Outcomes

SPIRIT-Outcomes 2022 Checklist (for combined completion of SPIRIT 2013 and SPIRIT-Outcomes 2022 items)[a]

Section	Item No.	SPIRIT 2013 Item	SPIRIT-Outcomes 2022 item	Location Reported[b]
**Administrative information**				
Title	1	Descriptive title identifying the study design, population, interventions, and, if applicable, trial acronym	-	
Trial registration	2a	Trial identifier and registry name. If not yet registered, name of intended registry	-	
	2b	All items from the World Health Organization Trial Registration Data Set	-	
Protocol version	3	Date and version identifier	-	
Funding	4	Sources and types of financial, material, and other support	-	
Roles and responsibilities	5a	Names, affiliations, and roles of protocol contributors	-	
	5b	Name and contact information for the trial sponsor	-	
	5c	Role of study sponsor and funders, if any, in study design; collection, management, analysis, and interpretation of data; writing of the report; and the decision to submit the report for publication, including whether they will have ultimate authority over any of these activities	-	
	5d	Composition, roles, and responsibilities of the coordinating centre, steering committee, endpoint adjudication committee, data management team, and other individuals or groups overseeing the trial, if applicable (see Item 21a for data monitoring committee)	-	
**Introduction**				
Background and rationale	6a	Description of research question and justification for undertaking the trial, including summary of relevant studies (published and unpublished) examining benefits and harms for each intervention	-	
	6b	Explanation for choice of comparators	-	
Objectives	7	Specific objectives or hypotheses	-	

For peer review only

SPIRIT | CONSORT OUTCOMES
Reporting Guidelines for Trial Outcomes

Section	Item No.	SPIRIT 2013 Item	SPIRIT-Outcomes 2022 item	Location Reported[b]
Trial design	8	Description of trial design including type of trial (eg, parallel group, crossover, factorial, single group), allocation ratio, and framework (eg, superiority, equivalence, noninferiority, exploratory)	-	
**Methods: Participants, interventions, and outcomes**				
Study setting	9	Description of study settings (eg, community clinic, academic hospital) and list of countries where data will be collected. Reference to where list of study sites can be obtained	-	
Eligibility criteria	10	Inclusion and exclusion criteria for participants. If applicable, eligibility criteria for study centres and individuals who will perform the interventions (eg, surgeons, psychotherapists)	-	
Interventions	11a	Interventions for each group with sufficient detail to allow replication, including how and when they will be administered (for specific guidance see TIDieR checklist and guide)	-	
	11b	Criteria for discontinuing or modifying allocated interventions for a given trial participant (eg, drug dose change in response to harms, participant request, or improving/worsening disease)	-	
	11c	Strategies to improve adherence to intervention protocols, and any procedures for monitoring adherence (eg, drug tablet return, laboratory tests)	-	
	11d	Relevant concomitant care and interventions that are permitted or prohibited during the trial	-	
Outcomes	12	Primary, secondary, and other outcomes, including the specific measurement variable (eg, systolic blood pressure), analysis metric (eg, change from baseline, final value, time to event), method of aggregation (eg, median, proportion), and time point for each outcome. Explanation of the clinical relevance of chosen efficacy and harm outcomes is strongly recommended	-	

For peer review only - http://bmjopen.bmj.com/site/about/guidelines.xhtml

Section	Item No.	SPIRIT 2013 Item	SPIRIT-Outcomes 2022 item	Location Reported[b]
	12.1		Provide a rationale for the selection of the domain for the trial's primary outcome	
	12.2		If the analysis metric for the primary outcome represents within-participant change, define and justify the minimal important change in individuals	
	12.3		If the outcome data collected are continuous but will be analyzed as categorical (method of aggregation), specify the cutoff values to be used	
	12.4		If outcome assessments will be performed at several time points after randomization, state the time points that will be used for analysis	
	12.5		If a composite outcome is used, define all individual components of the composite outcome	
Participant timeline	13	Time schedule of enrolment, interventions (including any run-ins and washouts), assessments, and visits for participants. A schematic diagram is highly recommended (see Figure)	-	
Sample size	14	Estimated number of participants needed to achieve study objectives and how it was determined, including clinical and statistical assumptions supporting any sample size calculations	-	
	14.1		Define and justify the target difference between treatment groups (eg, the minimal important difference)	
Recruitment	15	Strategies for achieving adequate participant enrolment to reach target sample size	-	
**Methods: Assignment of interventions (for controlled trials)**				
Allocation:				
Sequence generation	16a	Method of generating the allocation sequence (eg, computer-generated random numbers), and list of any factors for stratification. To reduce predictability of a random sequence, details of any planned restriction (eg, blocking) should be provided in a separate document that is unavailable to those who enrol participants or assign interventions	-	

For peer review only - http://bmjopen.bmj.com/site/about/guidelines.xhtml

Section	Item No.	SPIRIT 2013 Item	SPIRIT-Outcomes 2022 item	Location Reported[b]
Allocation concealment mechanism	16b	Mechanism of implementing the allocation sequence (eg, central telephone; sequentially numbered, opaque, sealed envelopes), describing any steps to conceal the sequence until interventions are assigned	-	
Implementation	16c	Who will generate the allocation sequence, who will enrol participants, and who will assign participants to interventions	-	
Blinding (masking)	17a	Who will be blinded after assignment to interventions (eg, trial participants, care providers, outcome assessors, data analysts), and how	-	
	17b	If blinded, circumstances under which unblinding is permissible, and procedure for revealing a participant's allocated intervention during the trial	-	
**Methods: Data collection, management, and analysis**				
Data collection methods	18a	Plans for assessment and collection of outcome, baseline, and other trial data, including any related processes to promote data quality (eg, duplicate measurements, training of assessors) and a description of study instruments (eg, questionnaires, laboratory tests) along with their reliability and validity, if known. Reference to where data collection forms can be found, if not in the protocol	-	
	18a.1		Describe what is known about the responsiveness of the study instruments in a population similar to the study sample	
	18a.2		Describe who will assess the outcome (eg, nurse, parent)	
	18b	Plans to promote participant retention and complete follow-up, including list of any outcome data to be collected for participants who discontinue or deviate from intervention protocols	-	

For peer review only - http://bmjopen.bmj.com/site/about/guidelines.xhtml

Section	Item No.	SPIRIT 2013 Item	SPIRIT-Outcomes 2022 item	Location Reported[b]
Data management	19	Plans for data entry, coding, security, and storage, including any related processes to promote data quality (eg, double data entry; range checks for data values). Reference to where details of data management procedures can be found, if not in the protocol	-	
Statistical methods	20a	Statistical methods for analysing primary and secondary outcomes. Reference to where other details of the statistical analysis plan can be found, if not in the protocol	-	
	20a.1		Describe any planned methods to account for multiplicity in the analysis or interpretation of the primary and secondary outcomes (eg, coprimary outcomes, same outcome assessed at multiple time points, or subgroup analyses of an outcome)	
	20b	Methods for any additional analyses (eg, subgroup and adjusted analyses)	-	
	20c	Definition of analysis population relating to protocol non-adherence (eg, as randomised analysis), and any statistical methods to handle missing data (eg, multiple imputation)	-	
**Methods: Monitoring**				
Data monitoring	21a	Composition of data monitoring committee (DMC); summary of its role and reporting structure; statement of whether it is independent from the sponsor and competing interests; and reference to where further details about its charter can be found, if not in the protocol. Alternatively, an explanation of why a DMC is not needed	-	
	21b	Description of any interim analyses and stopping guidelines, including who will have access to these interim results and make the final decision to terminate the trial	-	
Harms	22	Plans for collecting, assessing, reporting, and managing solicited and spontaneously reported adverse events and other unintended effects of trial interventions or trial conduct	-	

For peer review only

SPIRIT | CONSORT OUTCOMES
Reporting Guidelines for Trial Outcomes

Section	Item No.	SPIRIT 2013 Item	SPIRIT-Outcomes 2022 item	Location Reported[b]
Auditing	23	Frequency and procedures for auditing trial conduct, if any, and whether the process will be independent from investigators and the sponsor	-	
**Ethics and dissemination**				
Research ethics approval	24	Plans for seeking research ethics committee/institutional review board (REC/IRB) approval	-	
Protocol amendments	25	Plans for communicating important protocol modifications (eg, changes to eligibility criteria, outcomes, analyses) to relevant parties (eg, investigators, REC/IRBs, trial participants, trial registries, journals, regulators)	-	
Consent or assent	26a	Who will obtain informed consent or assent from potential trial participants or authorised surrogates, and how (see Item 32)	-	
	26b	Additional consent provisions for collection and use of participant data and biological specimens in ancillary studies, if applicable	-	
Confidentiality	27	How personal information about potential and enrolled participants will be collected, shared, and maintained in order to protect confidentiality before, during, and after the trial	-	
Declaration of interests	28	Financial and other competing interests for principal investigators for the overall trial and each study site	-	
Access to data	29	Statement of who will have access to the final trial dataset, and disclosure of contractual agreements that limit such access for investigators	-	
Ancillary and post-trial care	30	Provisions, if any, for ancillary and post-trial care, and for compensation to those who suffer harm from trial participation	-	
Dissemination policy	31a	Plans for investigators and sponsor to communicate trial results to participants, healthcare professionals, the public, and other relevant groups (eg, via publication, reporting in results databases, or other data sharing arrangements), including any publication restrictions	-	
	31b	Authorship eligibility guidelines and any intended use of professional writers	-	

For peer review only

SPIRIT | CONSORT OUTCOMES Reporting Guidelines for Trial Outcomes

Section	Item No.	SPIRIT 2013 Item	SPIRIT-Outcomes 2022 item	Location Reported[b]
	31c	Plans, if any, for granting public access to the full protocol, participant-level dataset, and statistical code	-	
**Appendices**				
Informed consent materials	32	Model consent form and other related documentation given to participants and authorised surrogates	-	
Biological specimens	33	Plans for collection, laboratory evaluation, and storage of biological specimens for genetic or molecular analysis in the current trial and for future use in ancillary studies, if applicable	-	

[a]It is strongly recommended that this checklist be read in conjunction with the SPIRIT (Standard Protocol Items: Recommendations for Interventional Trials) Statement paper for important clarification on the items. Amendments to the protocol should be tracked and dated. The SPIRIT checklist is copyrighted by the SPIRIT Group under the Creative Commons “Attribution-NonCommercial-NoDerivs 3.0 Unported” license and is reproduced with permission.
[b]Indicates page numbers and/or manuscript location: to be completed by authors.

For peer review only

For peer review only - http://bmjopen.bmj.com/site/about/guidelines.xhtml
