# Supplementary material for: Preconceptual administration of doxycycline in women with recurrent miscarriage and chronic endometritis: protocol for the Chronic Endometritis and Recurrent Miscarriage (CERM) trial, a multicentre, double-blind, placebo-controlled, adaptive randomised trial with an embedded translational substudy
Source: BMJ Open. 2023 Dec 1;13(12):e081470. doi: 10.1136/bmjopen-2023-081470 (PMC10693855; doi:10.1136/bmjopen-2023-081470)
Supplement: Reviewer comments [file bmjopen-2023-081470.reviewer_comments.pdf]

PEER REVIEW HISTORY

BMJ Open publishes all reviews undertaken for accepted manuscripts. Reviewers are asked to complete a checklist review form (**http://bmjopen.bmj.com/site/about/resources/checklist.pdf**) and are provided with free text boxes to elaborate on their assessment. These free text comments are reproduced below.

ARTICLE DETAILS

**TITLE (PROVISIONAL)**	Pre-conceptual administration of doxycycline in women with recurrent miscarriage and chronic endometritis: protocol for the Chronic Endometritis and Recurrent Miscarriage (CERM) trial, a multicentre, double-blind, placebo-controlled, adaptive randomised trial with an embedded translational sub-study
**AUTHORS**	Odendaal, Joshua; Black, Naomi; Bouliotis, Georgios; Guck, Jonathan; Underwood, Martin; Fisher, Joanne; Quenby, Siobhan

This article was not externally reviewed at BMJ Open. Protocols that have been independently assessed prior to submission to BMJ Open are usually fast-tracked to publication on the grounds that further substantial changes will not be possible. This independent assessment will usually be external, independent review for both a substantial grant award from a non-commercial or government funder and ethics approval.
